# Do you prefer to collaborate with students pursuing the same goals? – A network analysis of physical education classes

**DOI:** 10.1111/bjep.12757

**Published:** 2025-03-06

**Authors:** Annabell Schüßler, Cornelius Holler, Yannick Hill

**Affiliations:** ^1^ Institute of Sports & Sports Sciences Heidelberg University Heidelberg Germany; ^2^ Department of Human Movement Sciences, Faculty of Behavioral and Movement Sciences Vrije Universiteit Amsterdam, Amsterdam Movement Sciences Amsterdam The Netherlands; ^3^ Institute of Brain and Behaviour Amsterdam Amsterdam The Netherlands; ^4^ Lyda Hill Institute for Human Resilience University of Colorado Colorado Springs Colorado Springs Colorado USA

**Keywords:** achievement goals, ERGM, friendship, homophily, physical education, social networks

## Abstract

**Background:**

At school, students need to learn to collaborate with others to achieve common objectives. However, we are lacking insights into how students determine preferred collaboration partners, while multiple plausible factors, such as similar goal orientations, can be derived from the literature.

**Aims:**

We examined whether students prefer teammates in physical education based on similar achievement goals, stronger degrees of goal orientation, the same gender, and friendship.

**Sample:**

We recruited 364 students aged 10–16, across 16 classrooms in three German secondary schools.

**Methods:**

Social Network Analyses with Exponential Random Graph Models (ERGMs) are applied to identify relevant achievement‐goal dimensions for teammate selection and to assess preferences for collaborating with peers with similar or stronger degrees of goal orientation or with their friends.

**Results:**

Our findings indicate that students prefer to collaborate with peers who display similar levels of achievement‐goal orientations in physical education. Additionally, students prefer collaborating with friends and often select peers of the same gender, with boys being chosen more frequently than girls. When students do not pick their friends, they seek out peers with stronger degrees of goal orientation, specifically for goals aimed at winning.

**Conclusion:**

When collaborating in sports games, peers are faced with the dilemma of choosing between friends and the desire to win. Teachers should supervise the formation of groups and, depending on the aim of a particular lesson, should allocate students on the basis of different characteristics or let students choose their own group members.

## INTRODUCTION

Striving for competence, oftentimes referred to as *achievement‐goal pursuit* in the psychological literature, is a common aspect of our everyday lives and guides the behaviour of students, employees, and athletes (Weissman & Elliot, 2023). While more modern accounts of the goal‐setting literature shifted to predominantly individual‐level success (Daumiller & Hemi, 2025; Elliot, 1999; Hulleman et al., 2010), recent research supports the notion that peer relationships mimic the same superordinate goal structure (Laninga‐Wijnen et al., 2018). Specifically, children who pursue similar achievement goals seem to identify each other as friends. In sports games during physical education, where only one team wins, players create overarching goals subdivided into smaller objectives (van Yperen, 2022). Thus, students aim for various minor goals that lead them to succeed (Mascret et al., 2015). Selecting the right team is vital not just for winning but also for personal well‐being and achieving individual targets. Peers are excellent at noticing how quickly and willingly their classmates take on tasks (Laninga‐Wijnen et al., 2018; Palacios et al., 2024). Leveraging these observations, they employ a spectrum of criteria to select collaborators. Firstly, students may actively choose to collaborate with others who strive for the same goals, thereby pursuing the same superordinate goal (Altermatt & Pomerantz, 2003; Shin & Ryan, 2014). This preference for peers with similar characteristics is driven by the desire to ensure harmonious collaborations and to avoid conflicts caused by differences of opinion and attitudes. Secondly, students select teammates with a very strong motivation to achieve sports‐related goals to form teams that are driven to succeed, even if their motivational levels are not comparably high (Palacios et al., 2024). Thirdly, students may prefer to collaborate with their friends for the advantages of loyalty and trust (e.g., Hartl et al., 2015). Although some selection criteria within peer networks for choosing collaborators have been previously studied, these investigations often overlooked the context of the interactions, specifically within the setting of physical education (Palacios et al., 2024).

Thus, the aim of the current study is to investigate whether students in physical education display a stronger preference for collaborating with others based on their similarity in achievement goals, stronger degrees of goal orientations, or friendships. We adopted a social network approach to analyse relevant achievement‐goal dimensions for choosing teammates and to identify whether students select peers with the same goals, stronger degrees of goal orientation, or friends as teammates.

### Social relations in physical education

Social relations are shaped by mutual expectations, which may vary according to the type of relationship (Fuhse, 2022). For example, friendships typically involve a reciprocal bond characterized by trust and mutual support (Bukowski et al., 2009; Kitts & Leal, 2021). In contrast, the relationship between teammates often centres around a shared commitment to winning a competition or a common objective. Individuals tend to collaborate with teammates who share similar goals or characteristics to feel more comfortable (i.e., homophilic goals, McPherson et al., 2001). In this sense, adolescents adjust their academic ambitions and performance to align with those of their friends, highlighting how mutual social expectations shape individual aspirations (Vit et al., 2024).

However, individual team members who are lacking motivation or enthusiasm among highly motivated peers can disrupt the harmony. For example, a qualitative study conducted by Krieger (2005) involving 117 students revealed that the participation of highly engaged and less engaged students in joint physical activities often leads to the formation of subgroups based on different levels of engagement. This dynamic is associated with increased tensions, instances of discrimination, and a deterioration of the overall classroom environment. Consequently, a mutual disinterest in physical education emerges. To better understand these dynamics, imagine a volleyball game as an illustrative example: Some players may focus on refined play, such as executing two contacts to set up an attack, while others prioritize scoring directly to receive a point. This mismatch in approaches can create frustration among task‐oriented players and might lead to tension, undermining team cohesion.

Collaboration preferences may also extend to teammates with superior skills. This implies that individuals may prefer to be positively connected to the excellence of a person (Snijders & Lomi, 2019). The desire to win can lead players to acknowledge that teaming up with more motivated individuals increases their chances of success. An opposing principle for team formation would be *complementary*, where social relations are more likely to form between individuals with different skills, which may complement each other to maximize the chances for success (Snijders & Lomi, 2019).

At the same time, it is also possible that achievement goals take a secondary role, with peers prioritizing collaboration with friends to feel safe and supported (Hartl et al., 2015), even if they pursue different achievement goals than one does.

However, in friendships among highly motivated peers, the dynamic can be more complex: When friends share similar high achievement goals, they might also perceive each other as competitors, potentially leading to the dissolution of friendships among these highly motivated peers. As very different individuals are a less relevant source for comparison, friendships among dissimilar peers may be less threatening in the longer term (Festinger, 1954). Friendships between similar highly motivated peers might break down due to competition or unfair behaviours fostering trust issues and reducing the closeness of friendship (Garcia et al., 2020; Poortvliet, 2013). Conversely, when striving for high achievement goals, students might lean towards choosing friends who achieve less, as this strategy allows them to enhance their own self‐esteem through positive comparisons (Régner et al., 2007). Accordingly, children may also choose to collaborate during tasks with others outside of their friendship network with whom they have very little in common.

Thus, several competing forces seem to be at work when children choose the classmates they would like to collaborate with on a common task. It has recently been proposed that such choice preference may be analysed with so‐called *social networks* to identify the emerging group structures rather than relying on individual choice data (Steglich et al., 2010).

### Social networks

Social networks are a specific type of network model for social relationships where each node is represented by an individual and these individuals are interlinked through relations, called *ties* (sometimes referred to as *arcs* in graph theory, Fuhse, 2022). These networks offer a fruitful framework for examining the interdependence of social interactions and their social environment (Doehne et al., 2024; Hedström & Bearman, 2009; McFarland et al., 2014). In this context, attributes like goal orientation might be important individual actor attributes for the attainment of common goals in cooperative sports tasks.

#### Tie formation in social networks on a graph theoretical level

**FIGURE 1:**
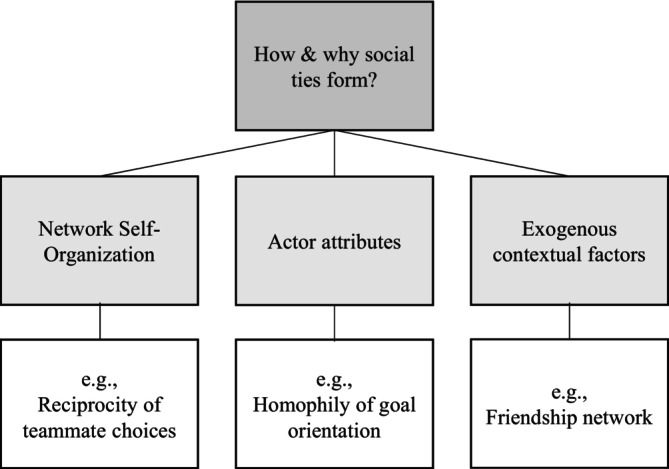
Processes of social tie formations discussed in this paper (following Lusher & Robins, 2013b).

On a technical level, tie formations in social relations can be explained by three main categories (see Figure 1). The first category, referred to as network self‐organization, suggests that ties can organize themselves based on the presence or absence of other ties. These patterns of organization, known as endogenous factors, arise “purely” from the network structure itself, without involving actor attributes or other network relations, such as reciprocity (Lusher & Robins, 2013b; Wang, Liu, et al., 2016). The second category focuses on actor attributes, demonstrating how students with specific binary attributes can influence the network structure. The third category examines whether the presence of a tie in one network can predict corresponding ties in another network of interest.

#### The principle of homophily for tie formation mechanisms

A central feature for understanding tie formation mechanisms is the principle of *homophily*. According to this principle, actors tend to establish connections with others who are similar to them (McPherson et al., 2001). Homophily manifests in various domains, such as academic characteristics (Prinstein & Dodge, 2008), norms and values (Lazarsfeld & Merton, 1954), or inherent characteristics like gender (McPherson et al., 2001). Lazarsfeld and Merton (1954) distinguish between two types of homophily. Status homophily concerns the similarity in social status, which considers sociodemographic dimensions like gender or attained attributes like religion. Value homophily focuses on internal states shaping future behaviour and includes attitudes, values, and beliefs. In school classes, the selection based on gender seems to be particularly prominent as younger children tend to almost exclusively choose other children of the same gender as their friends (Shrum et al., 1988). However, it may be argued that for cooperation within sports games, both status‐ (e.g., gender) and value homophily (e.g., goal orientation) are relevant factors guiding students in their choice of preferred teammates.

#### Dimensions of social networks

Whether or not network ties are homophilic depends on the meaning of these ties (Fuhse & Gondal, 2022). Multiple concepts have been developed to distinguish different meanings of network relations with numerous classifications of ties (see for an overview, Genkin et al., 2022). In the educational context, most of these ties entail the provision of resources like friendship and advice or affective and instrumental support (Lin, 2001; Zander et al., 2014). These relationships can be classified along a continuum ranging from affective (emphasizing emotional connections) to instrumental (focused on achievement specific goals) or from cooperation to competition. However, multiple types of ties tend to appear within the same context (i.e., *multiplexity*, Verbrugge, 1979). Such multiplex relationships extend emotional and instrumental exchanges simultaneously. For example, classmates with whom friendships are also maintained may not only function as cooperative and helpful learning partners but also provide emotional security.

### Achievement goals

Achievement goals are a prominent motivation construct that explores the factors behind an individuals' behaviour and are competence‐based strivings that individuals pursue (Elliot, 1999; Hulleman et al., 2010). Initially, these goals were categorized into two types: mastery goals, where individuals focus on personal improvement and skill development, and performance goals, where the emphasis is on outperforming others (Dweck & Leggett, 1988). However, this framework was later extended by the engagement with the task at hand per se (Mascret et al., 2015; van Yperen, 2022). Thus, three reference points for evaluating success can be distinguished: task‐based, self‐based and other‐based goals. Task‐based goals focus on executing specific behaviours (according to some specified criteria) like executing a free throw in basketball with a specific technique. Self‐based goals reference one's previous level of performance and focus on self‐development. Thus, a child adopting such a goal would strive to improve their performance. Finally, other‐based goals refer to outcomes that compare one's own performance to (specific) others. Within physical education, students may compare their own achievements to those of their classmates to evaluate their performance.

Achievement goals do not only define the frame of reference for competence striving, but they can also be distinguished on a second axis marked by approach and avoidance tendencies, forming the common 3 × 2 achievement goal framework (Elliot et al., 2011). Approach goals focus on approaching competence like winning against others or improving one's own performance, while avoidance goals focus on avoiding incompetence, including not losing or not underperforming. Thus, in physical education, a performance‐approach goal would imply a drive to demonstrate a better performance than other children (i.e., winning) and a mastery‐avoidance goal would be adopted by a child who wants to prevent performing below their previous level.

Goals can also change over time with the dynamics of the environment and the sequence of preceding events (Elliot & Dweck, 2005; Gernigon et al., 2015). In this context, a meta‐analysis by Lochbaum et al. (2023) found significant differences between physical education and the general academic context regarding achievement goals. Individuals tend to endorse all six achievement goals in sports, with large differences for task‐related and other‐approach goals (i.e., ultimately winning, van Yperen, 2022). In sports, completing tasks correctly and outperforming others are key, as winning ultimately depends on outperforming the competition (van Yperen et al., 2014). In generic educational settings, avoidance goals were favoured over approach goals, whereas in sports, approach goals were endorsed more than avoidance goals.

In some cases, an individual's goals are clearly apparent to the observer due to direct communication. More often, though, goals remain implicit and are not openly stated (Aarts et al., 2004). However, goals yield different overt behavioural patterns, such as effort, competitiveness, or task‐focused actions which may influence who a student identifies with the most (Weissman & Elliot, 2023; Shin & Ryan, 2014). Previous studies suggest that people easily infer goals from behaviour and that this process can occur automatically, without conscious awareness (Aarts et al., 2004; Hassin et al., 2005). In particular, peers can accurately assess each other's motivations and needs in group settings (Doeze Jager et al., 2017; Huelsnitz et al., 2020). For instance, students are moderately accurate in perceiving mastery‐based goals because they are expressed through consistent, observable behaviours, such as effort and engagement (Weissman & Elliot, 2023). However, performance‐based goals, which are more context‐specific, are harder to detect (Weissman & Elliot, 2023). This behavioural consistency may explain why children tend to collaborate with peers who pursue similar goals. Shared behaviours enhance team coordination, a critical factor in successful group performance (Silva et al., 2013).

Interestingly, individuals may not only choose to collaborate with others who display the same goal pursuit behaviour, but even minimal exposure to peers can induce changes in one's own attitudes and behaviours (e.g., van den Berg & Cillessen, 2015). In this context, friends play a direct role in shaping students' academic motivation (e.g., Altermatt & Pomerantz, 2003; Reindl, 2020). An explanation for this social phenomenon is goal contagion, where individuals tend to absorb the goals of those around them (Aarts et al., 2004). For example, King and Mendoza's (2020) study revealed that mastery‐approach, performance‐approach, and performance‐avoidance goals were contagious, while mastery‐avoidance goals did not exhibit the same effect. Close friends are particularly adept at predicting each other's motivations (Huelsnitz et al., 2020), and those who see each other as friends are more likely to pursue similar goals (Shin & Ryan, 2014).

However, Laninga‐Wijnen and colleagues (2018) point out that it is unclear whether the direction of the association between goals and friendship networks may be driven by the friendship group rather than by the goal orientation. Specifically, the friend group may begin to pursue similar goals after it has formed, rather than the group being formed as a result of common goals. In other words, frequent interactions between individuals lead to a convergence in their attitudes and behavioural tendencies (Veenstra & Steglich, 2012).

### The current study

The aim of the current study is to provide a deeper understanding of how students choose with whom they prefer to collaborate in sports games in physical education settings. There may be three explanatory mechanisms that could account for the collaboration in sports games. Firstly, individuals are naturally drawn to those who are very similar to them, which could potentially lead students to prefer collaborating with students sharing goals, resulting in a stronger sense of cohesion (Aarts et al., 2004; King & Mendoza, 2020; Shin & Ryan, 2014). Thus, we expect that students who have similar goal orientations in physical education prefer to collaborate in a sports team (Hypothesis 1).

Secondly, the achievement goals pursued by students may also play a vital role in fostering collaboration to ensure the desired outcomes are achieved. This is because students might want to collaborate with peers who possess high abilities, in our case, a strong goal orientation, as they believe that having engaged teammates could increase their chances of winning the game and achieving their objectives (Holler & Schüßler, 2024; Snijders & Lomi, 2019). Accordingly, students who have strong degrees of goal orientations in physical education are preferentially selected in sports games (Hypothesis 2).

Thirdly, previous research suggests that children are inclined to collaborate with their friends (e.g., Hartl et al., 2015). Interacting with friends in instrumental contexts might foster a supportive and safe environment, contributing to their emotional comfort, ultimately enhancing their enjoyment during sports games. Therefore, students primarily select their friends for collaboration in a sports game (Hypothesis 3).

To test these three hypotheses, we conducted cross‐sectional social network analyses for twenty school classes from three different schools to identify whether the overlap of the collaboration partners overlaps with the respective goal orientation and friendship networks. Thereby, we can identify if students prefer to work together with others whom they see as friends, who pursue similar goals, or who have high achievement‐goal orientations. Previous studies in the education context indicate that students prefer to work together with individuals of the same gender (e.g., Shrum et al., 1988). Accordingly, we included gender as a control variable in our model to address its potential impact on the observed collaboration dynamics in sports games.

## METHOD

### Participants and procedure

We collected data from 20 coeducational classes (grades 5 through 9) with a total of 425 students from three different secondary schools in North Rhine‐Westphalia (Germany) in summer 2022.^1^ Our final sample consisted of 364 students (202 girls; 157 boys, 5 non‐binary) representing 16 different classes. On average, 23.3 students per class participated (range = [16;28]) with a mean participation rate of 82.7% (range = [59.3%;100%]).

This study design and procedure were reviewed by an institutional ethics committee, and together with the university's data protection officer, we developed an economic pseudonymization procedure. With this in place, we distributed the questionnaires and pseudonymization list to the respective teachers of each class along with detailed instructions regarding the procedure. The teachers conducted the data collection with their class using either online (via tablet) or paper‐pencil questionnaires. Students were allowed to participate in the study if they had parental consent.

### Measures

To measure goal orientation in physical education, we used the 3 × 2 Achievement Goal Questionnaire (AGQ) for sports (Wang, Liu, et al., 2016), emphasizing task‐, self‐, and other‐based goals with an approach or avoidance valence. It is designed for use in the broader sport domain (Wang, Liu, et al., 2016). The questionnaire includes 18 items, with three items for each of the six subdimensions (Appendix S1). Participants responded on a 7‐point Likert scale ranging from “strongly disagree” to “strongly agree.” For example, within the “task‐approach” subdimension, participants responded to items like, “I aim to execute the skills correctly.”

These items were translated into German by a certified interpreter affiliated with the university, using the back translation method. Besides collecting sociodemographic data like gender, we used the peer nomination procedure (Wasserman & Faust, 1994) to measure different types of peer relations, covering personal relations like friendship as well as collaborative learning activities in physical education like collaborating in a sports team (see Table 1). These questions were introduced by situation descriptions so that students could place themselves in the related context (see Table 1). Although unlimited peer nominations might appear ideal, we used the fixed choice procedure, which allowed students to nominate no more than five other students for sports games and three other students for friendship (Wasserman & Faust, 1994). This approach was aimed at reducing the length of the questionnaire, preventing respondent fatigue from answering too many network‐related questions (Marsden, 2014). In our subsequent analyses, we used model fitting to address the constraints imposed by these two different kinds of nomination limits, ensuring the restricted outdegree distribution was appropriately accounted for in the analysis.

**TABLE 1:** Network name generators.

Social relation	Description for students	Item
Sports game	Imagine now that your sports teacher praises you for your great cooperation in recent lessons. As a reward, you may play a team game in this class. You may choose yourself with which classmates you would like to play on a team game. Answer the following questions: Name at most 5 students (numbers) who, in your opinion, apply to the question. Remember that you can choose girls and boys. You just cannot choose yourself!	Which classmates would you like to play with on a team?
Friendship	Please answer the last question. Name no more than three students (numbers) who, in your opinion, fit the question! You just cannot choose yourself!	Which classmates are among your friends

### Data analysis

Our relational network data do not meet the assumption of independence of observations because dyad‐level data include both senders and receivers of relationships (Dekker et al., 2007). Therefore, we use specific network analytic tools to analyse our dataset, which consists of both dyadic (i.e., networks) and monadic (i.e., attributes) data. Hence, we tested our hypotheses using cross‐sectional binary exponential random graph modelling (ERGM, Lusher & Robins, 2013a). The key principle of an ERGM is that the presence of a relationship tie depends on the occurrence of individual attributes or other relations (Robins et al., 2007). ERGMs allow us to explore the extent to which collaboration nominations and having the same characteristic of goal orientation co‐occur. Furthermore, ERGMs can account for how friendship and gender might influence this co‐occurrence (see Figure 1). On a graph theoretical level, this model takes the network as a graph of nodes and edges while considering the observed network as an example drawn from a probability distribution of all possible networks on a fixed set of nodes (Robins & Lusher, 2013). Different kinds of models are encompassed within ERGMs, each distinguished by the specific dependence assumption they are based on. As we assume a strong dyadic dependence, we applied the Markov chain Monte Carlo maximum likelihood estimation (MCMC MLE) method. This method is designed to generate random graph distributions based on a given set of parameter values (Robins & Lusher, 2013). These parameter values are adjusted by comparing distributions of random and observed graphs, iteratively repeated until they reach stability (Jiao et al., 2017; Robins & Lusher, 2013). In order to use explanatory node attribute covariates for the modelling of network ties, we apply Social Selection Models (SSMs), as suggested by Robins et al. (2001) and Wang, Robins, et al. (2016).

#### Model specification

For our models, we included variables from the three categories (Figure 1), commonly used in ERGMs: *Network self‐organization, actor attributes*, and *exogenous contextual factors*. For the selection of endogenous effects, we followed the example of Lusher and Robins (2013a, 2013c), who have successfully applied these effects to school children. They emphasized key endogenous network effects such as the arc effect, reciprocity, popularity, activity, and triadic closure for the school context (for a description of these effects: Appendix S2). In our analysis, we adopted all these effects, except for activity,^2^ to capture the underlying mechanisms shaping network formation (Table 2).

**TABLE 2:** Examples for common structural parameters of ERGMs (following Jiao et al., 2017 and Lusher & Robins, 2013a).

Network effect	Structure of point	Example	ERGM effect	Variable
Arc	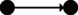	A student nominates another student as a teammate	Edges	Sports game network
Reciprocity	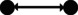	Two students nominate each other as teammates	Mutual	Sports game network
Popularity	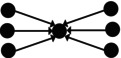	A few students receive a disproportionately high number of nominations for a sports game, while the majority receive only a few nominations	Gwidegree	Sports game network
Twopath	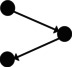	“My teammate's teammate is not my teammate”	Twopath	Sports game network
Triadic Closure	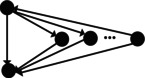	“My teammate's teammate is also my teammate”	Gwesp	Sports game network
Heterophily	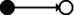	Students are more likely to nominate students who differ more in their goal orientation	Absdiff	Achievement goals
Homophily	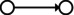	Students nominate those who have the same gender as teammates for a sports game	Nodematch	Gender
Receiver	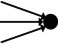	Students with a high goal orientation are more likely to be chosen as teammates	Nodeicov, nodeifactor	Achievement goals, gender
Sender	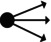	Students with high goal orientations are more likely to send ties than students with lower goal orientations	Nodeocov, nodeofactor	Achievement goals, gender
Other networks	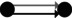	A friendship tie makes a teammate tie more likely	Edgecov	Friendship

At the level of actor attributes, we differentiate between receiver effect, sender effect, homophily, and heterophily (see Table 2). This means that an actor's attribute can influence its tendency to either initiate more ties (i.e., sender effect) or receive more ties (i.e., *receiver effect*, Lusher & Robins, 2013b). In our study, the principle of homophily describes the tendency for students who share similar characteristics to be more likely to form connections. For categorical attributes, like gender, this can be examined through matching similar attributes. Heterophily, the opposite concept, is applied to continuous attributes, such as achievement goals, allowing us to test homophily technically through the absolute difference between the students' attributes. In ERGMs, a positive coefficient for the absolute difference effect shows that larger differences in an attribute increase the likelihood of a connection, which we refer to as a heterophily effect. Theoretically, a smaller coefficient suggests a higher degree of homophily. For clarity, we use “heterophily” in tables and text when discussing absolute differences in ERGM analyses, while “homophily” is used more generally when not directly referencing this specific coefficient, as it is the more familiar term.

On the one hand, homophily, the choice of matching attributes, can be applied for binary or categorical attributes (in our case, gender), while heterophily, its opposite, is used for continuous attributes to test homophily using the absolute difference between students' attributes, which we employed for goal orientation. Following Lusher and Robins (2013a), we extended sender and receiver effects for goal orientations to control these effects for homophily. A significant homophily effect indicates when teammates select other teammates based on shared goal orientation, independent of competing effects. Lastly, we included friendship as an *exogenous contextual factor* into our model, as friendship might play a substantial role in tie formation processes in the school setting.

### Analytic strategy

To examine our first and second hypotheses, we employed our first ERGM. This model investigates the pure effects of the six subdimensions of goal orientation as important actor attributes, coupled with the effects of network self‐organization (Table 2) as control variables on sports game networks. In a second ERGM, we included gender as a standard control variable that is not associated with the criterion variable and the six subdimensions of goal orientation to determine if the effects of goal orientations are still significant when accounting for gender. To test our third hypothesis, we conducted our third ERGM with friendship nominations and gender, designed to assess the propensity of students to preferentially collaborate with friends in sports games. Our fourth ERGM was created to integrate goal orientation, friendship, and gender, with the intent to understand their interactive effects on sports team selections, under the control of the endogenous network self‐organizations. This model is important to examine how friendship forms multiplex relations with the criterion variable, the sports game collaborations. If the second model, which focuses on goal orientation and gender, indicates strong effects, but these effects are then changed in the fourth model when friendship is added, it would suggest that friendship might mediate or even dominate the impacts of goal orientation and gender.

Note that testing all models is essential, given that we lack longitudinal data and must therefore analyse the cross‐sectional relations between friendship, goal orientation, and gender. By incrementally incorporating friendship and gender into the models, we can observe the resulting variations and better understand the relations between these effects.

To interpret the findings across the 16 classrooms, we followed Rambaran and colleagues (2022) to first model ERGMs for each classroom separately. We then synthesized the resulting parameter estimates through meta‐analyses. For this, we computed a random‐effects meta‐analysis with a restricted maximum likelihood estimator (REML) for each parameter using the R package “metafor” (Viechtbauer, 2010). We chose a random‐effects instead of a fixed‐effects meta‐analysis as we expect variations in effects due to differing peer and class cultures (Veenstra & Lodder, 2022). Using a meta‐analytic approach additionally mitigates the risk for type 1 errors, because even if one model identifies a parameter as statistically significant by chance for one specific classroom, it will very likely not retain significance when pooled with all other models in the meta‐analysis. For our ERGMs, we used the R packages “sna” and “ergm” from the statnet suite (Statnet Development Team et al., 2003–2023). The R Code is provided in the E‐Supplement (Appendix S4).

## RESULTS

### Descriptive results

The descriptive analysis reveals differences in the network parameters between sports game and friendship networks (Table 3). The latter exhibit lower levels of density, centralization, and average degrees. The difference in density and average degrees can be attributed to the more restrictive nomination limit of three friends, as opposed to the five allowed in the game network. The combination of lower reciprocity and higher centralization in sports game networks suggests a pattern where connections tend to cluster around peers who might be seen as highly motivated or important players (*in social network terms, this is known as preferential attachment*), unlike in friendship networks.

**TABLE 3:** Descriptive statistics for game networks, friendship networks, and goal orientation.

Measures	Mean (SD)
**Game networks**	
Density	.17 (.03)
Centralization	.18 (.05)
Reciprocity	.49 (.09)
Average degree	3.62 (2.91)
**Friendship networks**	
Density	.11 (.02)
Centralization	.10 (.03)
Reciprocity	.62 (.10)
Average degree	2.48 (1.53)
**Goal orientation**	
Task‐approach	6.09 (.78)
Task‐avoidance	5.84 (.96)
Self‐approach	6.25 (.86)
Self‐avoidance	6.13 (.94)
Other‐approach	4.24 (1.60)
Other‐avoidance	5.14 (1.45)

All achievement goals (except for other‐approach goals) appear to be relatively strong, with average scores above 5 on a 7‐point scale (see Table 3). The subscales of the 3 × 2 AGQ for sports demonstrated internal consistencies ranging from acceptable to very good: Task‐approach (*α* = .76), task‐avoidance (*α* = .81), self‐approach (*α* = .81), self‐avoidance (*α* = .80), other‐approach (*α* = .91), and other‐avoidance (*α* = .80).

### Hypothesis testing

In Tables 4 and 5, we present the meta‐analysis of five ERGMs, combining parameter estimates into a mean value (log‐odds) along with corresponding standard errors and significance levels. The interpretation of ERGMs mimics a logistic regression, which means that the coefficients (log‐odds) indicate how the likelihood of a tie changes in a response to a one‐unit change in the predictor (van der Pol, 2019). Goodness‐of‐fit statistics indicate good model fits for all models (Appendices S3 and S4).

**TABLE 4:** Results from meta‐analysis of class‐wise ERGMs with sports game networks as dependent variable.

Parameter	Model 1: Goals			Model 2: Goals + gender			Model 3: Gender + friendship			Model 4: Goals + gender + friendship		
	*b*	SE	*p*	*b*	SE	*p*	*b*	SE	*p*	*b*	SE	*p*
**Network self‐organization**												
Arc	**−2.685**	.863	.002	**−3.185**	.965	<.001	**−2.484**	.218	<.001	**−4.112**	1.296	.002
Reciprocity	**1.289**	.131	<.001	**1.285**	.135	<.001	.**554**	.144	<.001	.**536**	.159	<.001
Two‐paths	**−.323**	.026	<.001	**−.319**	.028	<.001	**−.206**	.027	<.001	**−.280**	.035	<.001
Popularity	.**800**	.247	.001	.**881**	.272	.001	**−.743**	.222	<.001	.232	.297	.435
Triadic closure	**1.260**	.066	<.001	**1.126**	.068	<.001	**1.064**	.076	<.001	**1.034**	.082	<.001
**Actor attributes**												
Goal orientation												
Task‐approach receiver	−.053	.066	.424	−.041	.077	.596				.070	.100	.481
Task‐approach sender	−.059	.123	.630	−.056	.137	.685				−.201	.213	.346
Task‐approach heterophily	**−.124**	.056	.028	−.132	.071	.064				−.050	.096	.6
Task‐avoidance receiver	.137	.104	.190	.153	.113	.177				.102	.117	.382
Task‐avoidance sender	.101	.113	.375	.128	.112	.254				.204	.168	.225
Task‐avoidance heterophily	−.004	.066	.958	.028	.080	.727				.033	.091	.717
Self‐approach receiver	.086	.067	.202	.135	.092	.140				.227	.170	.183
Self‐approach sender	−.178	.109	.103	−.136	.150	.365				−.016	.231	.944
Self‐approach heterophily	**−.131**	.066	.048	−.150	.079	.058				−.037	.109	.737
Self‐avoidance receiver	−.067	.146	.646	−.064	.154	.677				.032	.187	.863
Self‐avoidance sender	.028	.113	.805	−.066	.126	.603				−.056	.163	.732
Self‐avoidance heterophily	.014	.060	.809	.026	.070	.714				.016	.089	.856
Other‐approach receiver	.**131**	.031	<.001	.**129**	.034	<.001				.**170**	.042	<.001
Other‐approach sender	−.015^a^	.050	.768	−.025^a^	.054	.644				−.011^a^	.068	.87
Other‐approach heterophily	**−.089**	.023	<.001	**−.106**	.027	<.001				−.071	.052	.172
Other‐avoidance receiver	**−.113**	.033	< .001	**−.125**	.038	<.001				**−.191**	.074	.009
Other‐avoidance sender	−.004^a^	.065	.955	.030^a^	.079	.703				−.011^a^	.102	.917
Other‐avoidance heterophily	−.032	.029	.269	−.020	.033	.551				−.069	.054	.207
Gender (1 = girl, 2 = boy, 3 = non‐binary)												
Boy receiver				.**478**	.127	<.001	.**506**	.084	<.001	.**628**	.149	<.001
Non‐binary receiver				.740^b^	.481	.124	.702^c^	.367	.056	.982^b^	.582	.092
Boy sender				**−.402**	.145	.006	−.**362** ^a^	.122	.003	−.378^a^	.204	.064
Non‐binary sender				.080^c^	.783	.919	.140^c^	.615	.819	.014^c^	1.145	.990
Same Gender				.**689**	.058	<.001	.**278**	.080	<.001	.**358**	.090	<.001
**Exogenous contextual factors**												
Friendship networks							**2.945**	.125	<.001	**3.466**	.211	<.001

*Note*: Bold values indicate statistically significant coefficients (*p* < .05.).

^a^ Indicates 15 classes in the meta‐analysis.

^b^ Indicates 4 classes in the meta‐analysis.

^c^ Indicates 3 classes in the meta‐analysis.

**TABLE 5:** Interaction between Other‐approach and Friendship Networks.

Parameter	*b*	SE	*p*
**Network self‐organization**			
Arc	**−2.394**	.429	<.001
Reciprocity	.**532**	.153	<.001
Two‐paths	**−.218**	.029	<.001
Popularity	**−.567**	.254	.026
Triadic closure	**1.069**	.083	<.001
**Actor attributes**			
Goal orientation			
Other‐approach receiver	.**198**	.037	<.001
Other‐approach receiver × friendship	**−.215**	.105	.041
Other‐approach sender	−.004	.055	.940
Other‐approach heterophily	−.060	.041	.142
Other‐approach heterophily × friendship	.**254**	.114	.026
Other‐avoidance receiver	−.117	.065	.071
Other‐avoidance receiver × friendship	.115	.114	.316
Other‐avoidance sender	−.016	.060	.788
Other‐avoidance heterophily	−.024	.050	.628
Other‐avoidance heterophily × friendship	−.054	.115	.638
Gender (1 = girl, 2 = boy, 3 = non‐binary)			
Boy receiver	.**532**	.117	<.001
Non‐binary receiver	.658^a^	.468	.160
Boy sender	**−.429**	.141	.002
Non‐binary sender	.4612^b^	.699	.510
Same gender	.**329**	.087	<.001
**Exogenous contextual factors**			
Friendship	**3.009**	.729	<.001

*Note*: Bold values indicate statistically significant coefficients (*p* < .05.).

^a^ Indicates 4 classes in the meta‐analysis.

^b^ Indicates 3 classes in the meta‐analysis.

In our ERGMs, all network self‐organization parameters, barring popularity in the final model, were significant and confirmed the anticipated effect directions. These findings align with standard network self‐organization dynamics observed in school‐based peer group formations (for a detailed interpretation: Appendix S5).

In our first model (Table 4), we tested the six subdimensions of goal orientations to determine which of these most accurately reflects team selection. In the task‐approach domain, there is a significant negative heterophily effect (*b* = −.12, *p* = .03), suggesting that students want to collaborate with peers who strive for the same task accuracy. However, other aspects of task‐approach and task‐avoidance are not significant. The same trend was observed for self‐approach with a significant negative heterophily effect (*b* = −.13, *p* = .05) indicating that students prefer collaboration with those who share similar learning progress. The remaining elements of self‐approach and self‐avoidance lack significance. In the domain of other approach, a significant negative receiver effect (*b* = .13, *p* < .001) is observed, alongside a significant negative heterophily effect (*b* = −.09, *p* = < .001), while activity is not significant. This not only denotes a tendency for students who display stronger degrees of goal orientations to be preferred as teammates, but also implies a preference for collaboration with peers who share a similar focus on outperforming others. Other‐avoidance showed a significant negative receiver effect (*b* = −.11, *p* = < .001), indicating that students who have a high degree of other‐avoidance are less likely to be chosen as collaborators. Sender and heterophily effects are not significant.

In *Model 2* (Table 4), with gender added as a control variable, task‐ and self‐approach goals lost their significance in comparison to *Model 1*. The receiver effect of other‐approach (*b* = .13, *p* < .001) and the heterophily effect of other‐approach (*b* = −.11, *p* < .001) and other‐avoidance (*b* = −.13, *p* < .001) remained significant. When examining the effects of gender, the analysis revealed that boys are more likely to be picked than girls (receiver effect: *b* = .48, *p* < .001) and that boys are less likely to nominate their peers (sender effect: *b* = −.40, *p* < .001). The parameter *same gender* also yields a significant positive effect (*b* = .69, *p* < .001), indicating a prevalent tendency towards forming teams with members of the same gender.

In *Model 3* (Table 4), which explores the role of friendship and gender for sports collaboration, we found a significant positive effect of friendship networks (*b* = 2.95, *p* < .001). Additionally, the gender‐based analysis yielded the same directional effects as observed in Model 2.

To evaluate the impact of similar goals, higher degrees of goal orientation, or friendship on teammate selection, with gender as a controlling factor, we constructed a fourth model (Table 4). Our results demonstrate no significant trends in task‐ or self‐related approaches and avoidances. However, we observed significant receiver effects in the domain of other‐approach (*b* = .17, *p* < .001) and other‐avoidance (*b* = −.19, *p* < .01), reflecting nuanced preferences in team selections based on stronger degrees of other‐orientations to be preferred as teammates. Moreover, friendship showed a significant positive effect (*b* = 3.47, *p* < .001). Furthermore, compared to the second model, we noted an increase in the receiver effect, particularly when accounting for friendship. This suggests that, even when not considering friendship, students who are motivated to outperform others are more frequently chosen as teammates in sports games. Second, the heterophily effect associated with other‐approach diminishes in the presence of friendship ties. Lastly, the other‐avoidance receiver effect became significant when controlling for friendship networks, which indicates that students who are particularly motivated to not underperform compared to their peers become even more desirable choices as teammates in sports games when controlling for friendship ties.

### Exploratory analysis: The role of friendship

Based on our findings from the initial four ERGMs, we observed a strong relation between friendship ties and goal orientation in predicting collaboration in team settings, indicating that these constructs are intertwined. Therefore, we developed an additional model (*Model 5*) to explore how other‐approach and other‐avoidance goals impact the likelihood of students playing together on a team, contingent on whether a friendship bond exists. Simply put, *Model 5* helps us determine which characteristics of other‐approach and other‐avoidance are relevant when individuals choose friends for sports collaboration, and which goal characteristics matter when selecting peers who are not friends.

Examining the results of *Model 5* (Table 5), we encountered a negative interaction effect for other‐approach (receiver), despite the positive significance of both individual main effects. This suggests that friendship may diminish the role of other‐approach orientations in the formation of collaborative ties within a team. When students do not choose their friends for a sports game, they tend to prefer peers with a high degree of other‐approach orientation.

Additionally, while the heterophily for other‐approach orientation was not significant on its own, there was a notable positive interaction between heterophily in other‐approach orientation and friendship ties. This indicates that friends share similar levels of other‐approach orientation.

## DISCUSSION

At school, children have to learn how to collaborate with others and function in a team. However, we know very little about the criteria they employ to identify suitable collaborators (Palacios et al., 2024). Given that successful task completion is strongly coupled to effective goal setting, students may look for similar superordinate goals to choose their collaborators. However, students may also be likely to prefer collaborating with their friends to maintain harmonious relationships. Therefore, the aim of the current study was to examine the determinants of preferred collaboration partners in physical education. Specifically, we investigated whether students prefer teammates based on similar achievement goals, stronger degrees of goal orientations, or friendship. We applied social network analyses with ERGMs to assess this question and control for endogenous network effects. Our findings indicate that Hypothesis 1, which posits that similar goals play a role in sports teammates' selections, is partially supported. This is evidenced by significant effects observed independently of friendship factors and by a notable interaction between other‐approach homophily and friendship, indicating that students tend to select friends based on their shared desire to win and such friendships, in turn, shape their educational ambitions (Model 5) (Laninga‐Wijnen et al., 2018).

Furthermore, Hypothesis 2, which suggests students with strong degrees of goal orientations in physical education are favoured for sports team selection, is also partially supported. This is limited to the dimensions of other‐orientation, accounting for friendship and gender. If students nominate their friends for sports collaborations, the friends can have a low other approach (Model 5). Friends tend to select their peers based on loyalty, irrespective of their desire to outperform others, which could promote inclusivity. This underscores the protective buffering effect of friendship, which could potentially reduce the number of students isolated due to poor motivational states in sports contexts. Conversely, in the absence of friendship considerations, students with a high other‐approach orientation are preferred, indicating a more instrumental approach (Model 5) (Palacios et al., 2024).

These findings align with and extend the insights of Hemi et al. (2024), who demonstrated that smaller close peer groups are more important for shaping mastery goals, while the broader class environment plays a role for performance goals. For our study, it is possible that, when it comes to other‐approach orientation, students might select teammates from the broader class rather than choosing only from their close friendship group. Similarly, it's plausible in our case that task approach (similar to mastery goals in Hemi's study) becomes less significant once friendship is controlled for. As Hemi found, homophily in task‐related goals (like mastery) tends to occur predominantly within close friendships and is most visible in those relationships. Therefore, when the impact of friendship is accounted for, task‐related goals might no longer have a strong independent effect, since homophily in task approach is more likely to be observed among friends.

Lastly, Hypothesis 3 is supported, suggesting that friendships play a dominant role in team selection for sports games. The fact that friendship is an important criterion for sports games aligns with existing research underscoring the important role of friendship in shaping peer relations within school settings (Hartl et al., 2015).

In sports settings specifically, other‐approach and other‐avoidance might be the most important dimensions of achievement goals (van Yperen, 2022; van Yperen et al., 2014). Ultimately, winning hinges on outperforming the competition. High‐achieving individuals who are closely matched in abilities may view one another as rivals, which could strain friendships (Festinger, 1954). In contrast, relationships between people with differences in abilities are less likely to involve such comparisons, making these friendships more stable over time (Régner et al., 2007).

### Limitations and future directions

A notable limitation of our study is the ceiling effects observed in four out of the six subdimensions of goal orientation. As mentioned in the descriptive statistics, all achievement goals, except for other‐approach goals, show relatively high average scores above 5 on a 7‐point scale (see Table 3), suggesting a potential social desirability bias.

Additionally, some individuals may have difficulty accurately assessing their own goals (e.g., over‐ or under‐estimators). To address this, it would be beneficial to incorporate peer assessments in future research to determine if peers can accurately perceive these goals in this context. Our study found that goal orientations and friendships are intertwined. Similarly, Laninga‐Wijnen and colleagues (2018) highlight that peers with congruent goal orientations might naturally gravitate towards forming friendships or they influence each other in their goal orientation over time (Laninga‐Wijnen et al., 2018). However, due to the cross‐sectional nature of our data, determining whether these patterns are a result of selection or socialization cannot be determined in relation to our study. To understand the evolution of these constructs, subsequent research must employ longitudinal data collection to investigate whether individuals choose friends based on shared goal orientations, or if goal contagion (socialization) occurs over time, resulting in greater similarity. Moreover, future research may consider classroom norms, as characteristics such as high goal orientation serve as criteria for selection and collaboration only when they are recognized as shared norms within the classroom (Veenstra & Lodder, 2022). Our goal was to explore how goal orientation may be relevant for generic sports game collaborations. However, there may be sport‐specific patterns for choosing teammates. Future research should investigate whether perceived achievement goals and preferred team collaborations differ depending on the specific type of sports game being considered.

For practical applications in physical education, intervention studies that explore how different types of team compositions can target various objectives, both based on the curriculum and personal goals, may provide a fruitful avenue. Such research would enable us to offer more concrete pedagogical guidelines, equipping educators with strategies to foster positive collaboration while effectively managing the balance between friendship and competitiveness in group assignments. For instance, an intervention study by Hartl et al. (2015) in an educational context found that grouping students by friendship, rather than by varying levels of performance, leads to greater learning success in computer science classes. In the context of physical education, it would be important for peers to feel comfortable in their learning groups in order to promote lifelong sporting activity. Simultaneously, sports games become truly enjoyable when teams of comparable skill levels compete against one another and thus induce a challenge (Hill et al., 2024). Therefore, in forming teams, both socio‐emotional and performance aspects must be considered in light of both homophily and heterophily factors. A key prerequisite for this is that educators develop a deeper understanding of the significance of peer relationships within the learning context. Consequently, future teacher training programs should be designed to better equip educators with the skills to assess and interpret peer dynamics and student motivation, as well as their influence on academic outcomes. Only when teachers are capable of evaluating these factors can they strategically structure group work and social interactions in the classroom to optimize learning success.

## CONCLUSION

The aim of our study was to identify collaboration preferences of students in physical education, emphasizing the role of their goal orientations, and friendships. Our findings reveal that students preferentially pick their friends, who have similar goal orientations. If they do not pick their friends, they are looking for others with stronger degrees of goal orientation, specifically for goals aimed at winning. This research highlights the complexity of team formation, balancing both personal relations and the drive to win. This can guide future work on how teachers can strategically assemble social relations in physical education to achieve educational objectives in sports.

## AUTHOR CONTRIBUTIONS

**Annabell Schüßler:** Conceptualization; investigation; writing – original draft; methodology; visualization; writing – review and editing; formal analysis; project administration. **Cornelius Holler:** Visualization; software; formal analysis; data curation; methodology; writing – review and editing. **Yannick Hill:** Writing – review and editing; writing – original draft; supervision.

## CONFLICT OF INTEREST STATEMENT

None declared.

## Supporting information


Appendices S1–S5

## Data Availability

The data that support the findings of this study are available from the corresponding author upon reasonable request.

## Footnotes

1 We analysed a portion of this data in relation to a different research question (see Holler & Schüßler, 2024).

^2^
Due to the limited number of outgoing nominations, the outdegree distribution was fairly constrained and could not be modelled by the activity effect (gwodegree).
