# Supplementary material for: Do you prefer to collaborate with students pursuing the same goals? – A network analysis of physical education classes
Source: Br J Educ Psychol. 2025 Mar 6;95(4):1266–85. doi: 10.1111/bjep.12757 (PMC12590936; doi:10.1111/bjep.12757)
Supplement: Supplementary file 1 — Appendices S1–S5 [file BJEP-95-1266-s001.pdf]

Do You Prefer to Collaborate with Students Pursuing the same Goals? – A Network Analysis of Physical Education Classes

Supplementary Material

Contents

Appendix 1: Goal Orientation Scale and Translation **2**

Appendix 2: Description of Network Endogenous Effects **3**

Appendix 3: Interpretation of Goodness of Fit **3**

Appendix 4: Detailed Model Specification and Goodness of Fit **4**

Model 1: Goals 4

R Code 4

Goodness of Fit Plots 6

Model 2: Goals & Gender 12

R Code 12

Goodness of Fit Plots 15

Model 3: Friendship & Gender 21

R Code 21

Goodness of Fit Plots 23

Model 4: Full Model 29

R Code 29

Goodness of Fit Plots 32

Model 5: Interaction Model 38

R Code 38

Goodness of Fit Plots 41

Appendix 5: Interpretation of Network Endogenous Effects **47**

References **48**

Appendix 1: Goal Orientation Scale and Translation

Subscale	English Original	German Translation
Task Approach	I aim to execute the skills correctly	Ich möchte die (Bewegungs-)Aufgaben korrekt ausführen.
	I strive to apply the right tactics and strategies	Ich bemühe mich, die richtigen Taktiken und Strategien anzuwenden.
	I want to execute every technique successfully	Ich möchte jede Technik korrekt ausführen.
Task Avoidance	I avoid making a lot of technical errors	Ich vermeide es, viele technische Fehler zu machen.
	I avoid applying the wrong tactics and strategies	Ich vermeide es, die falschen Taktiken und Strategien anzuwenden.
	I avoid making a lot of mistakes	Ich vermeide es, viele Fehler zu machen.
Self Approach	I want to perform better than previous performances	Ich möchte bessere Leistungen als bei früheren Leistungsüberprüfungen erzielen.
	I aim to do well relative to how well I have done in the past on similar challenges.	Mein Ziel ist es, im Vergleich zu dem, was ich in der Vergangenheit bei ähnlichen Herausforderungen geleistet habe, zu übertreffen.
	My goal is to do better than I normally do	Mein Ziel ist es, besser abzuschneiden, als ich es sonst tue.
Self Avoidance	I avoid performing worse than I normally do	Ich vermeide es, schlechtere Leistungen als sonst zu erbringen.
	I want to avoid performing poorly compared to my typical level of performance	Ich möchte vermeiden, im Vergleich zu meinem typischen Leistungsniveau schlecht abzuschneiden.
	My goal is to avoid doing worse than I have done on previous similar challenges	Mein Ziel ist es, zu vermeiden, schlechter abzuschneiden, als ich es bei früheren ähnlichen Herausforderungen getan habe.
Other Approach	It is important for me to perform better than others	Es ist wichtig für mich, besser als andere zu sein.
	It is important for me to do well compared to others	Es ist wichtig für mich, im Vergleich zu anderen gut abzuschneiden.
	My goal is to do better than most other players	Mein Ziel ist es, besser als die meisten anderen Spieler:innen zu sein.
Other Avoidance	I just want to avoid performing worse than others	Ich möchte nur vermeiden, dass ich schlechter als andere abschneide.
	My goal is to avoid performing worse than everyone else	Mein Ziel ist es, zu vermeiden, schlechter als alle anderen abzuschneiden.
	It is important for me to avoid being one of the worst performers in the group	Es ist wichtig für mich, zu vermeiden, dass ich nicht der/die schlechteste in der Gruppe bin.

Appendix 2: Description of Network Endogenous Effects

The *arc* effect (edges) acts analogously to an intercept in linear regression and represents the fundamental propensity for the occurrence of ties, forming a baseline for the network´s connectivity (Lusher & Robins, 2013a). On a graph theoretical level, *reciprocity* is the likelihood of actors "to send ties to those from whom they receive a tie" (Block, 2015, p. 164). *Popularity* is the tendency for centralization, which means that actors are more likely to send ties to those who receive more nominations from others (Stadtfeld & Amati, 2021). *Activity* is the complementary principle which describes the tendency of some actors to be more likely to send more ties compared to others (Lusher & Robins., 2013a). *Triadic closure* is a graph-theoretical concept which refers to the tendency of an actor to connect with others who are already connected to their existing contacts, creating closed triads (Robins et al., 2009). A *two-path* is a structure where actor A is connected to actor B and B is connected to C, but A and C are not connected, representing correlations between in-and out-degrees (Lusher & Robins, 2013a; Robins et al., 2009).

Appendix 3: Interpretation of Goodness of Fit

Assessing goodness of fit in ERGM is done by comparing the observed network statistics to those from networks simulated based on the fitted model. The analyses show results for 5 metrics: *model statistics*, *outdegree*, *indegree*, *edge-wise shared partners* and *geodesic distance*.

The simulated networks were always able to mimic the *model statistics*. The *outdegree* distribution is modeled fairly well across all classes and models. Even though the maximum number of nominations was limited, controlling for this constraint in the modeling notably improved model fit. The *indegree* and *edge-wise shared partners* distributions were similarly well modeled and showed only minor deviances. Most models were accurate in modeling the *geodesic distance* between nodes, the models of class 7 had some deviances.

Overall, the goodness of fit statistics indicate good model fits for all models, even though models 4 and 5 had the best fits out of all models. The plots detailing every model's goodness of fit can be found in Appendix 3.

Appendix 4: Detailed Model Specification and Goodness of Fit

Model 1: Goals

R Code

```
#### Model 1: Goals ####
ergm_sp_goals_list <- vector(mode = "list", length=length(SP_mn_only_list))
for(i in 1:16){
  # leave out other approach and other avoidance sender effects for class 14
  if(i == 14){
    ergm_sp_goals_list[[i]] <- ergm(SP_mn_only_list[[i]] ~
                                      edges +
                                      mutual +
                                      twopath +
                                      gwidegree(0.5,fixed=T) +
                                      gwesp(0.5,fixed=T) +
                                      nodeicov("tappr") +
                                      nodeocov("tappr") +
                                      absdiff("tappr") +
                                      nodeicov("tavoi") +
                                      nodeocov("tavoi") +
                                      absdiff("tavoi") +
                                      nodeicov("sappr") +
                                      nodeocov("sappr") +
                                      absdiff("sappr") +
                                      nodeicov("savoi") +
                                      nodeocov("savoi") +
                                      absdiff("savoi") +
                                      nodeicov("oappr") +
                                      #nodeocov("oappr") +
                                      absdiff("oappr") +
                                      nodeicov("oavoi") +
                                      #nodeocov("oavoi") +
                                      absdiff("oavoi"),
                                    control = control.ergm(seed = 2),
                                    constraints = ~bd(maxout = 5)
    )
  }else{
    ergm_sp_goals_list[[i]] <- ergm(SP_mn_only_list[[i]] ~
                                      edges +
                                      mutual +
                                      twopath +
                                      gwidegree(0.5,fixed=T) +
                                      gwesp(0.5,fixed=T) +
                                      nodeicov("tappr") +
                                      nodeocov("tappr") +
                                      absdiff("tappr") +
                                      nodeicov("tavoi") +
                                      nodeocov("tavoi") +
                                      absdiff("tavoi") +
                                      nodeicov("sappr") +
                                      nodeocov("sappr") +
                                      absdiff("sappr") +
```

```
                                               nodeicov("savoi") +
                                               nodeocov("savoi") +
                                               absdiff("savoi") +
                                               nodeicov("oappr") +
                                               nodeocov("oappr") +
                                               absdiff("oappr") +
                                               nodeicov("oavoi") +
                                               nodeocov("oavoi") +
                                               absdiff("oavoi"),
                                             control = control.ergm(seed = 1),
                                             constraints = ~bd(maxout = 5)
    )
  }
}

#### Meta-Analysis ####
# create data frame with coefficients and standard errors
ergm_sp_goals_df <- ergm_sp_goals_list %>%
  lapply(broom::tidy) %>%
  purrr::reduce(full_join,by="term") %>%
  .[,which(c(1,rep(c(1,1,0,0,0),times=16))==1)]

# create vector with parameter names
par_sp_goals <- ergm_sp_goals_df$term

# create a list of data frames with coefficients and standard errors for each parameter
sp_goals_pre_meta_list <- vector(mode="list",length=length(par_sp_goals)) %>%
  `names<-`(par_sp_goals)
for(i in 1:length(par_sp_goals)){
  sp_goals_pre_meta_list[[i]] <- ergm_sp_goals_df[i,2:ncol(ergm_sp_goals_df)] %>%
    as.numeric %>%
    matrix(ncol=2,byrow=TRUE) %>%
    as.data.frame %>%
    `colnames<-`(c("coef","se"))
}

# run meta-analysis on each parameter
sp_goals_ma_list <- vector(mode = "list", length=length(par_sp_goals)) %>%
  `names<-`(par_sp_goals)
for(i in 1:length(par_sp_goals)){
  sp_goals_ma_list[[i]] <- rma(yi=coef,sei=se,data=sp_goals_pre_meta_list[[i]],
                               control = list(stepadj=0.5,maxiter=1000))
}

# summary table
lapply(sp_goals_ma_list,broom::tidy) %>%
  purrr::reduce(rbind) %>%
  cbind(par_sp_goals,.) %>%
  mutate(estimate = round(estimate,4),
         std.error = round(std.error,4),
         statistic = round(statistic,4),
         p.value = round(p.value,4)) %>%
  select(c(1,4,5,7))
```

Goodness of Fit Plots

```
## [1] "Class 1"
```

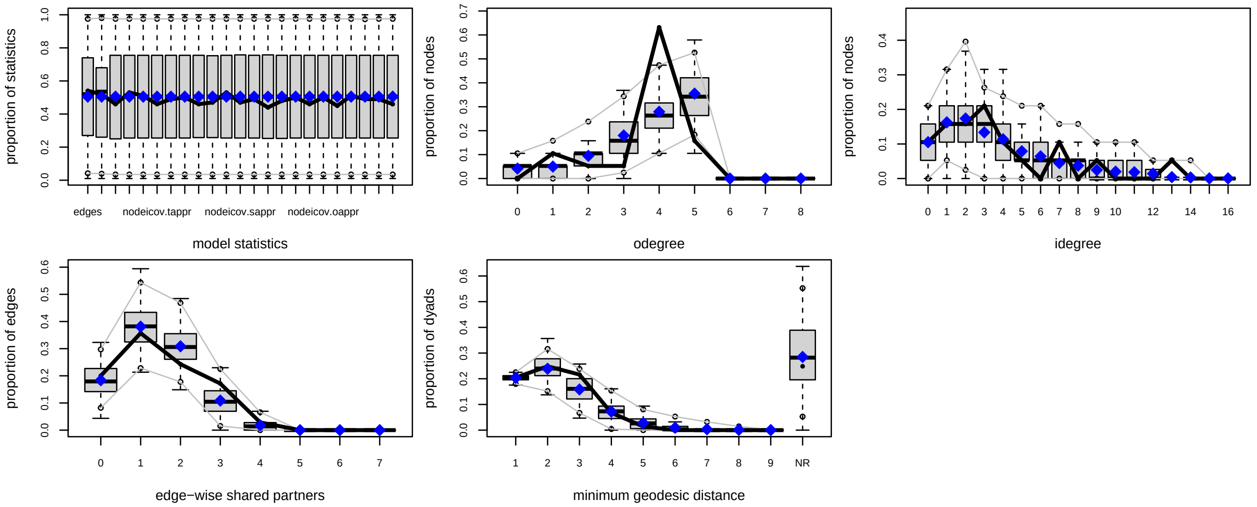


```
## [1] "Class 2"
```

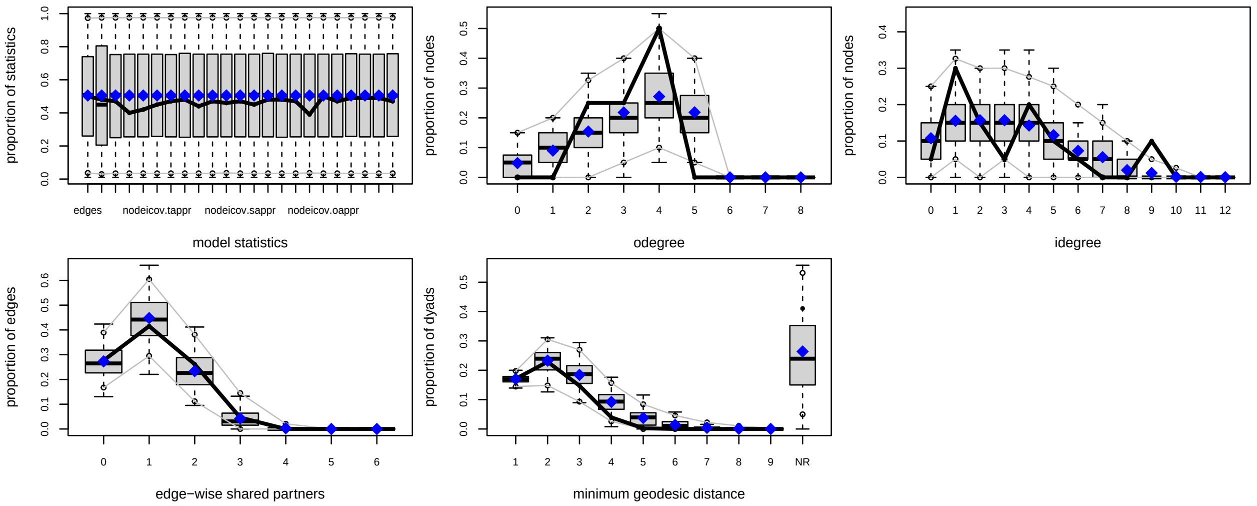


```
## [1] "Class 3"
```

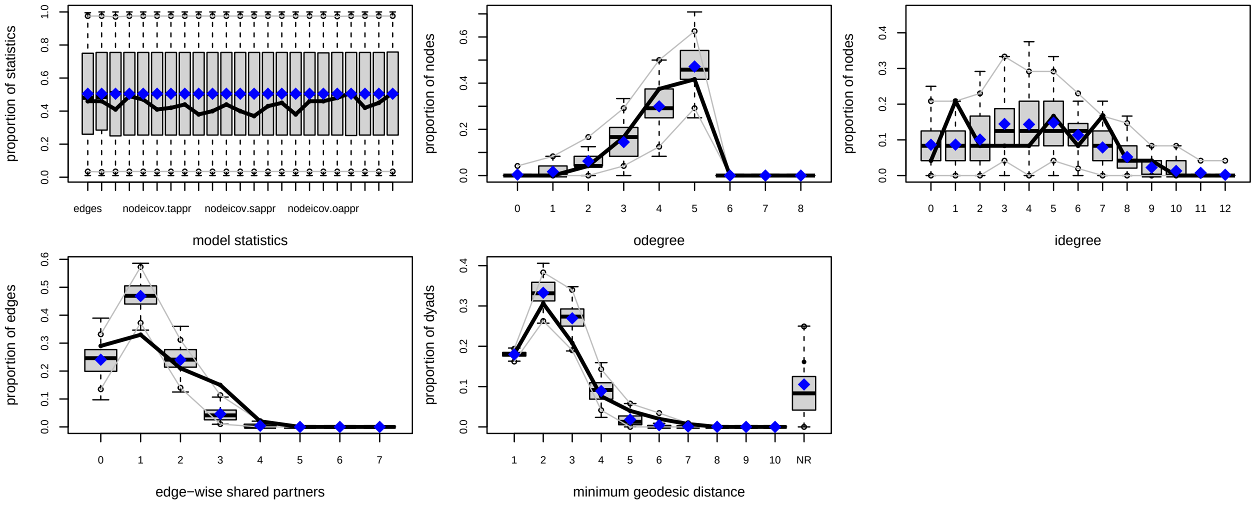

```
## [1] "Class 4"
```

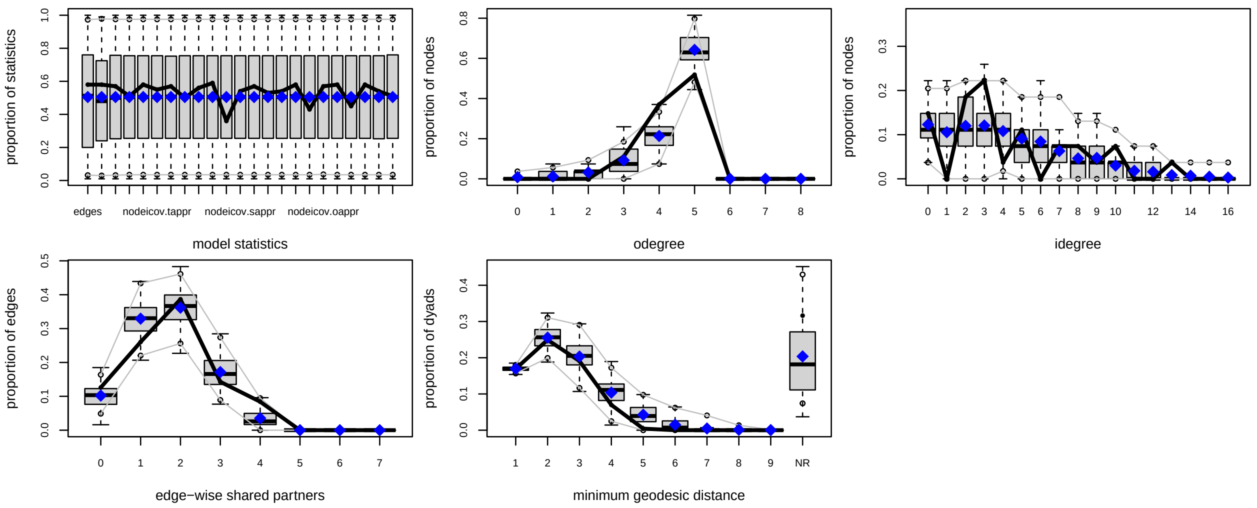


```
## [1] "Class 5"
```

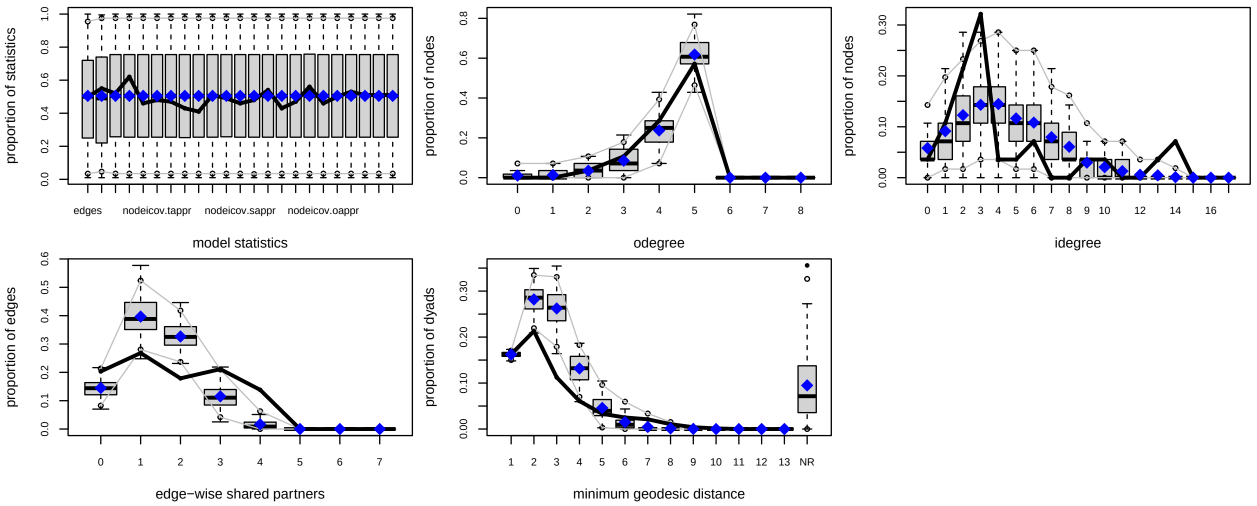


```
## [1] "Class 6"
```

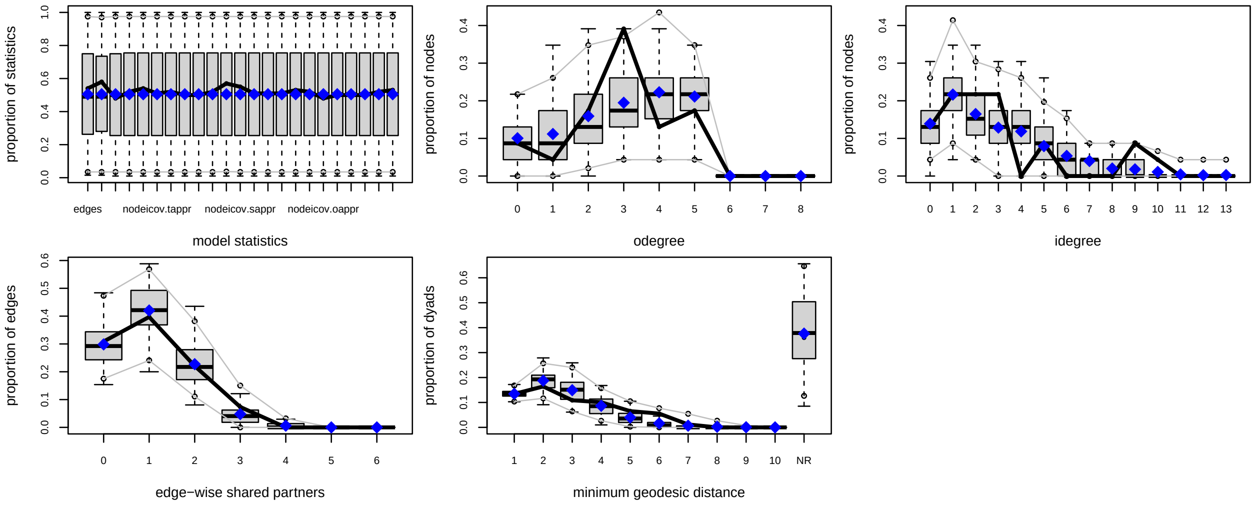

```
## [1] "Class 7"
```

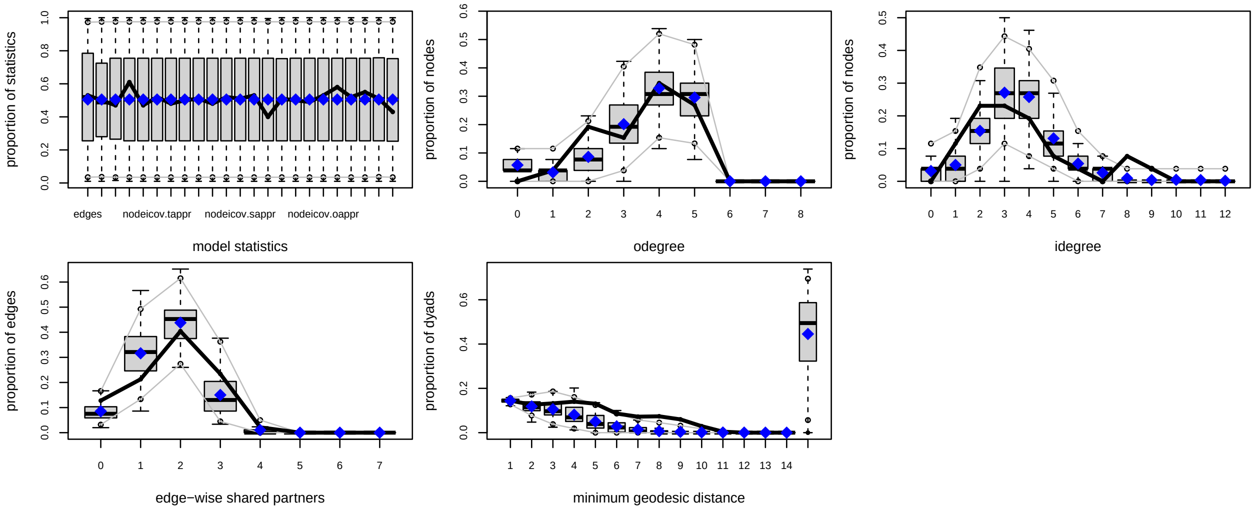


```
## [1] "Class 8"
```

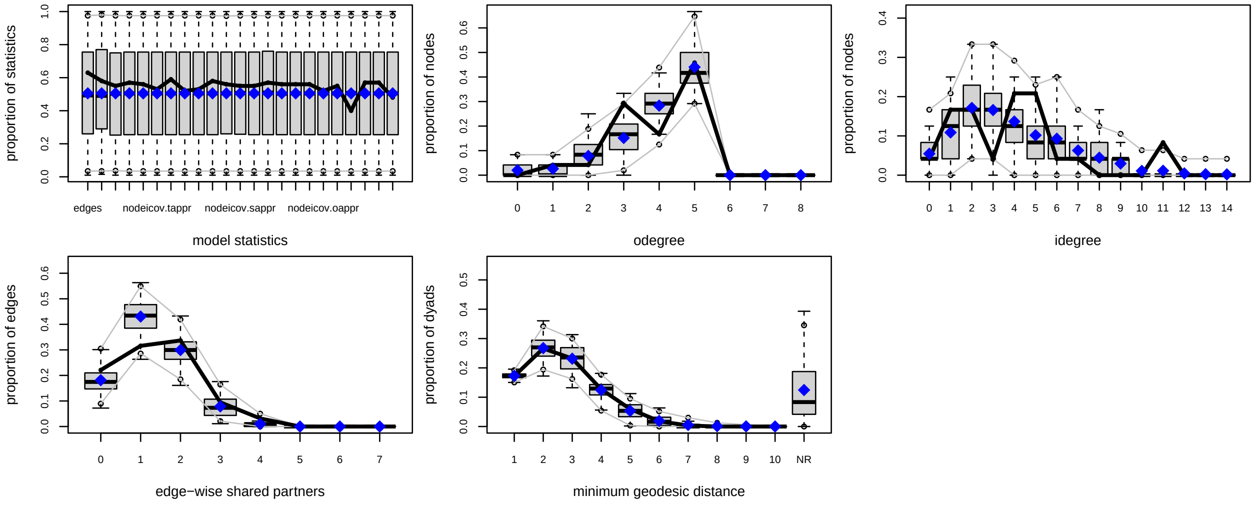


```
## [1] "Class 9"
```

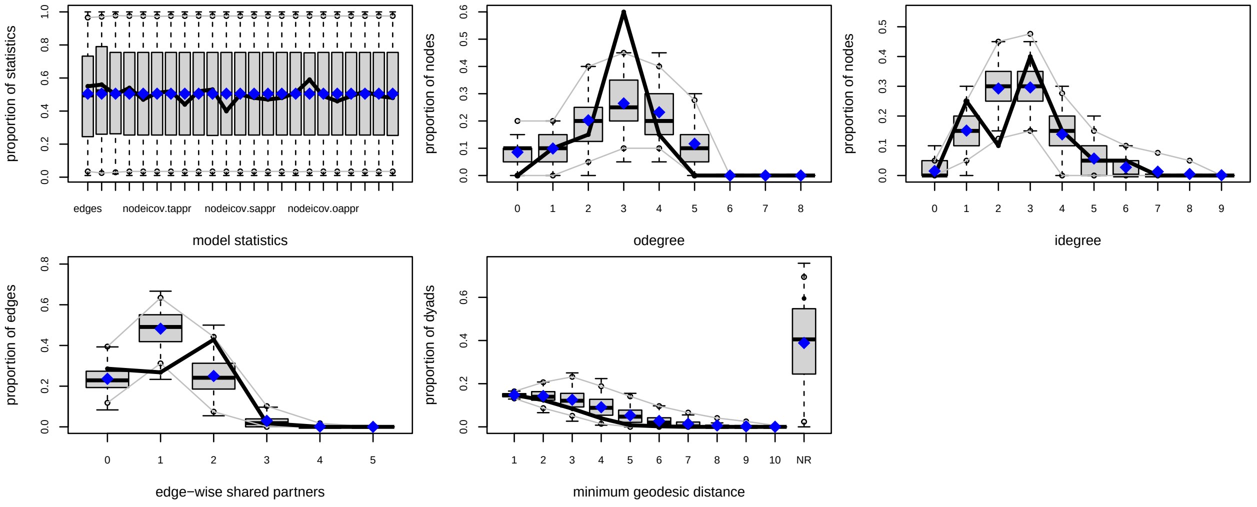

[1] "Class 10"

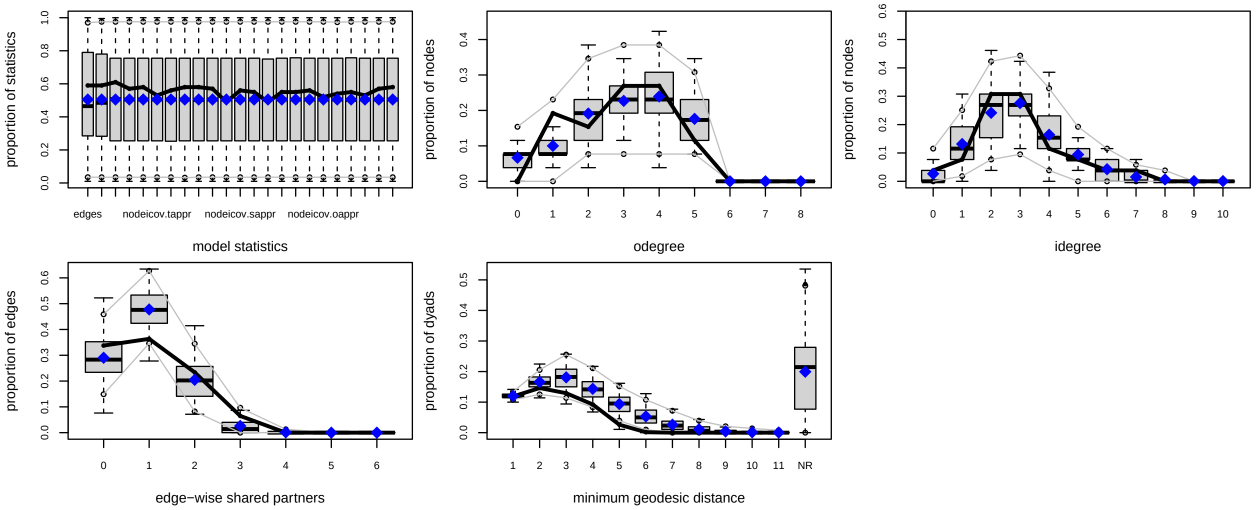

[1] "Class 11"

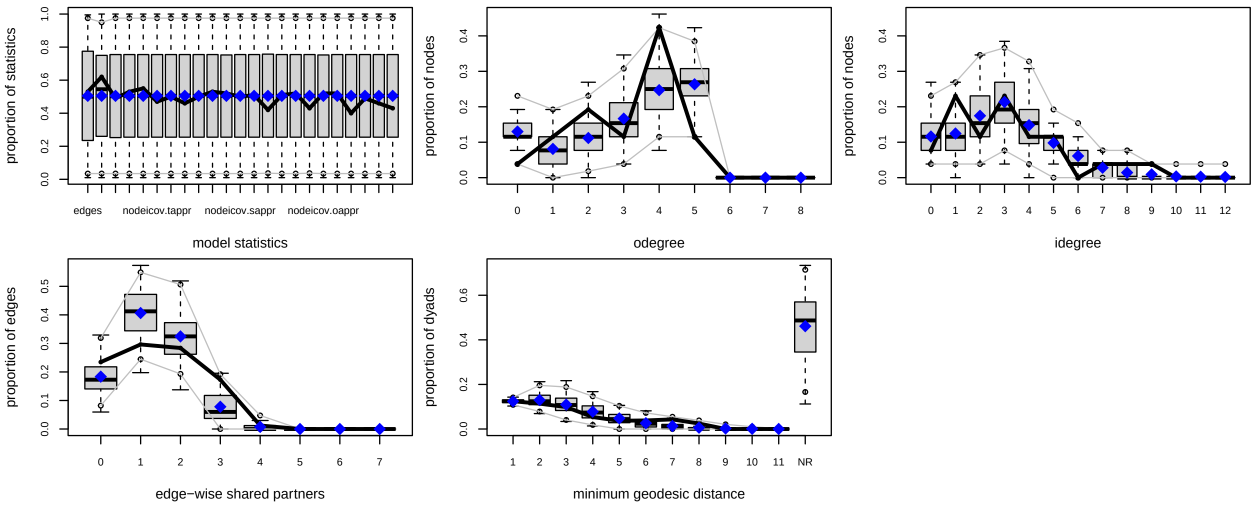

[1] "Class 12"

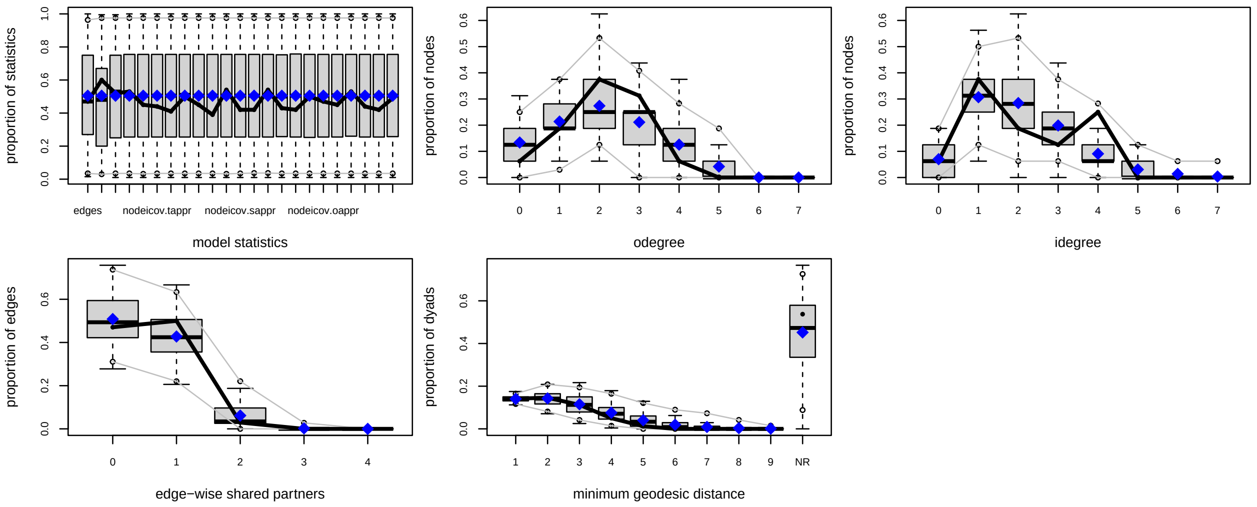

[1] "Class 13"

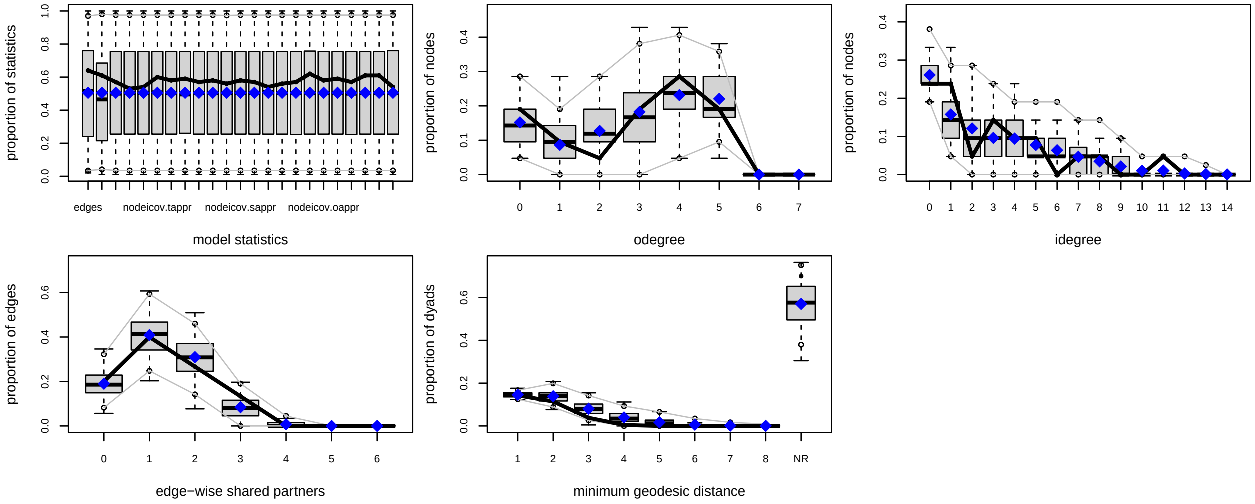

[1] "Class 14"

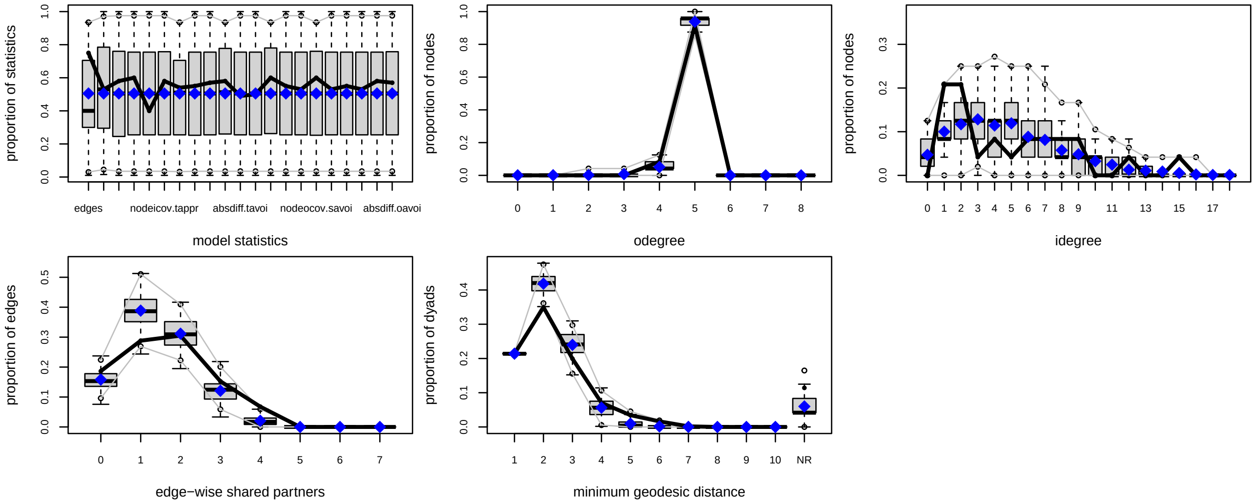

[1] "Class 15"

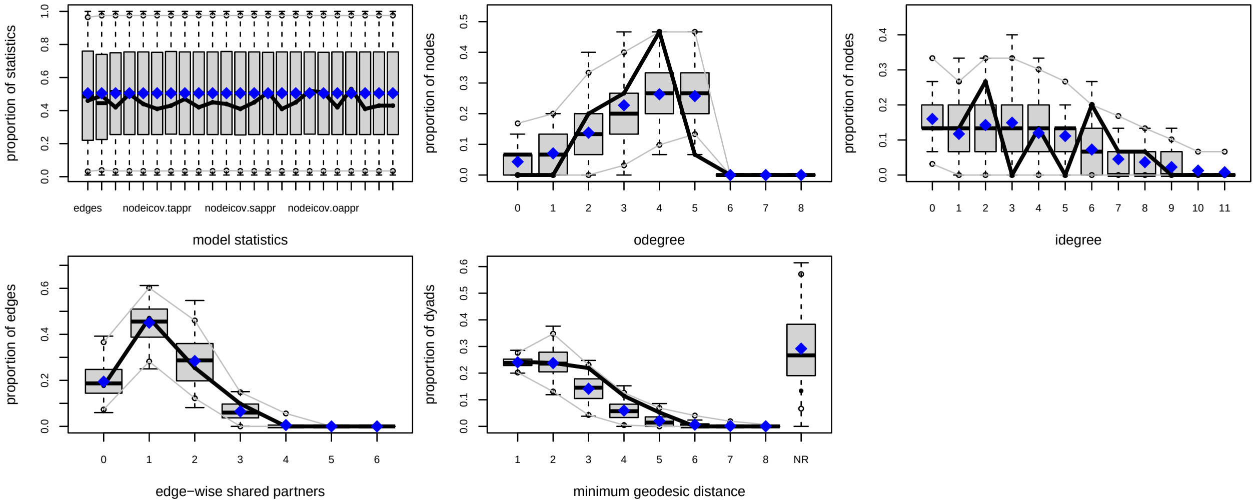

[1] "Class 16"

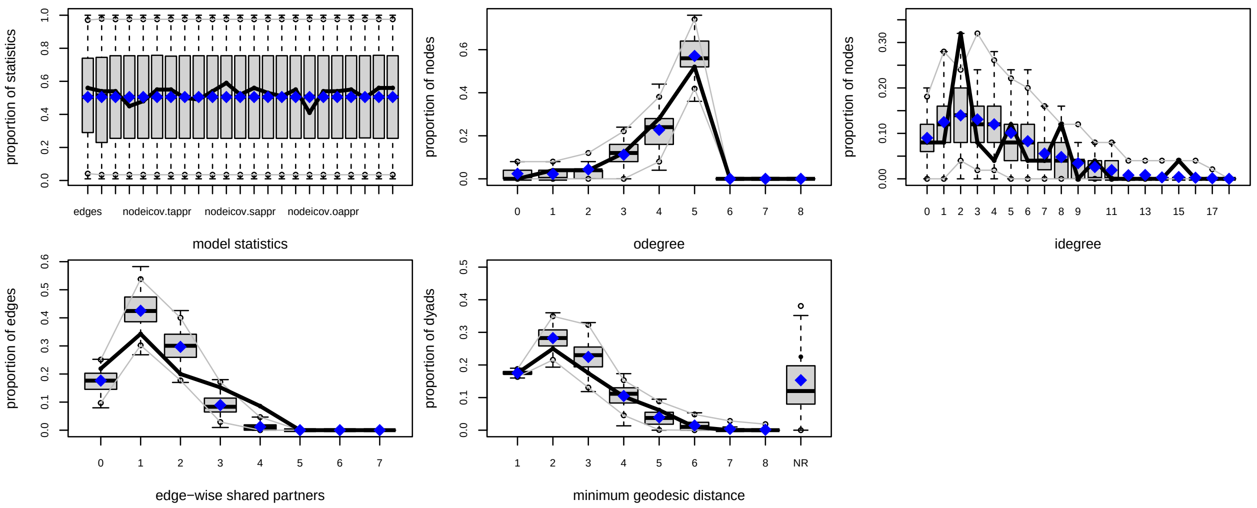

proportion of statistics
edges
nodeicov.tappr
nodeicov.sappr
nodeicov.oappr
model statistics
proportion of nodes
odegree
proportion of nodes
idegree
proportion of edges
edge−wise shared partners
proportion of dyads
minimum geodesic distance
NR

Model 2: Goals & Gender

R Code

```
#### Model 2: Goals and Gender ####
ergm_sp_goals_gender_list <- vector(mode = "list", length=length(SP_mn_only_list))
for(i in 1:16){
  # leave out other approach & avoidance as well as gender sender effect for class 14
  if(i == 14){
    ergm_sp_goals_gender_list[[i]] <- ergm(SP_mn_only_list[[i]] ~
                                              edges +
                                              mutual +
                                              twopath +
                                              gwidegree(0.5,fixed=T) +
                                              gwesp(0.5,fixed=T) +
                                              nodeicov("tappr") +
                                              nodeocov("tappr") +
                                              absdiff("tappr") +
                                              nodeicov("tavoi") +
                                              nodeocov("tavoi") +
                                              absdiff("tavoi") +
                                              nodeicov("sappr") +
                                              nodeocov("sappr") +
                                              absdiff("sappr") +
                                              nodeicov("savoi") +
                                              nodeocov("savoi") +
                                              absdiff("savoi") +
                                              nodeicov("oappr") +
                                              #nodeocov("oappr") +
                                              absdiff("oappr") +
                                              nodeicov("oavoi") +
                                              #nodeocov("oavoi") +
                                              absdiff("oavoi") +
                                              nodeifactor("gender") +
                                              #nodeofactor("gender") +
                                              nodematch("gender"),
                                            control = control.ergm(seed = 2),
                                            constraints = ~bd(maxout = 5)
    )
  }else{
    ergm_sp_goals_gender_list[[i]] <- ergm(SP_mn_only_list[[i]] ~
                                              edges +
                                              mutual +
                                              twopath +
                                              gwidegree(0.5,fixed=T) +
                                              gwesp(0.5,fixed=T) +
                                              nodeicov("tappr") +
                                              nodeocov("tappr") +
                                              absdiff("tappr") +
                                              nodeicov("tavoi") +
                                              nodeocov("tavoi") +
                                              absdiff("tavoi") +
                                              nodeicov("sappr") +
                                              nodeocov("sappr") +
```

```
                                                  absdiff("sappr") +
                                                  nodeicov("savoi") +
                                                  nodeocov("savoi") +
                                                  absdiff("savoi") +
                                                  nodeicov("oappr") +
                                                  nodeocov("oappr") +
                                                  absdiff("oappr") +
                                                  nodeicov("oavoi") +
                                                  nodeocov("oavoi") +
                                                  absdiff("oavoi") +
                                                  nodeifactor("gender") +
                                                  nodeofactor("gender") +
                                                  nodematch("gender"),
                                                control = control.ergm(seed = 1),
                                                constraints = ~bd(maxout = 5)
    )
  }
}

#### Meta-Analysis ####
ergm_sp_only_friendship_df <- ergm_sp_only_friendship_list %>%
  lapply(broom::tidy) %>%
  purrr::reduce(full_join,by="term") %>%
  .[,which(c(1,rep(c(1,1,0,0,0),times=16))==1)]

par_sp_only_friendship <- ergm_sp_only_friendship_df$term

sp_only_friendship_pre_meta_list <- vector(mode="list",
                                          length=length(par_sp_only_friendship)) %>%
  `names<-`(par_sp_only_friendship)
for(i in 1:length(par_sp_only_friendship)){
  sp_only_friendship_pre_meta_list[[i]] <-
    ergm_sp_only_friendship_df[i,2:ncol(ergm_sp_only_friendship_df)] %>%
    as.numeric %>%
    matrix(ncol=2,byrow=TRUE) %>%
    as.data.frame %>%
    `colnames<-`(c("coef","se"))
}

sp_only_friendship_ma_list <- vector(mode = "list",
                                    length=length(par_sp_only_friendship)) %>%
  `names<-`(par_sp_only_friendship)
for(i in 1:length(par_sp_only_friendship)){
  sp_only_friendship_ma_list[[i]] <-
    rma(yi=coef,sei=se,data=sp_only_friendship_pre_meta_list[[i]],
        control = list(stepadj=0.5,maxiter=1000))
}

# summary table
lapply(sp_only_friendship_ma_list,broom::tidy) %>%
  purrr::reduce(rbind) %>%
  cbind(par_sp_only_friendship,.) %>%
  mutate(estimate = round(estimate,4),
```

```
          std.error = round(std.error,4),
          statistic = round(statistic,4),
          p.value = round(p.value,4)) %>%
  select(c(1,4,5,7))
```

Goodness of Fit Plots

```
## [1] "Class 1"
```

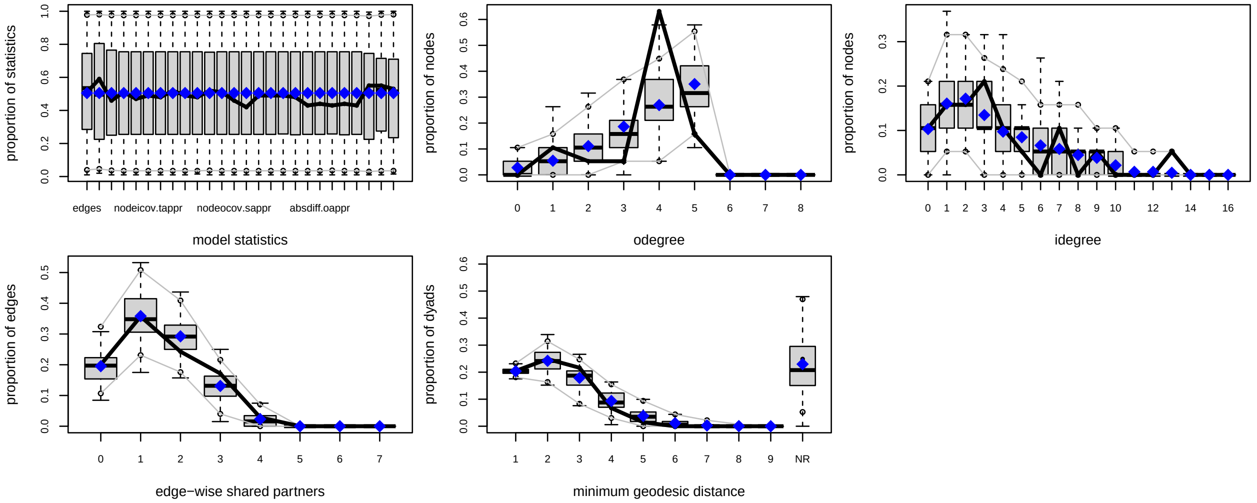


```
## [1] "Class 2"
```

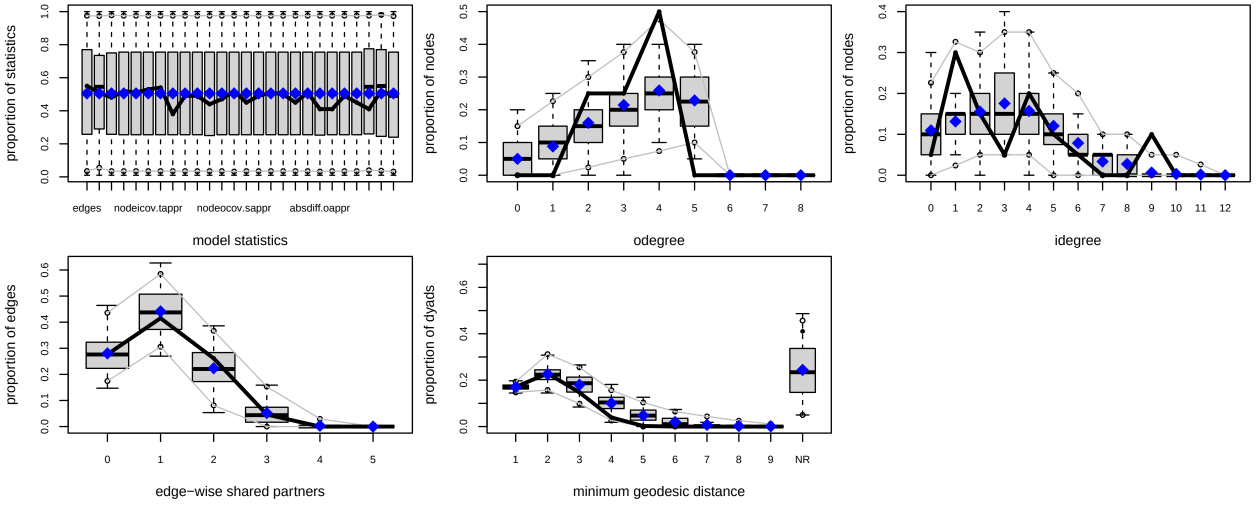


```
## [1] "Class 3"
```

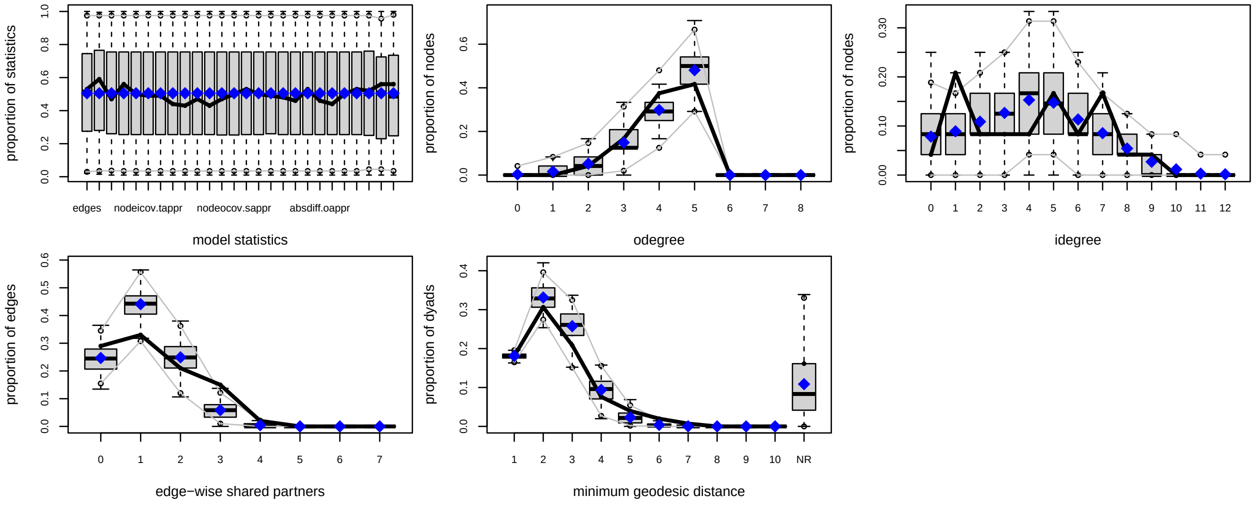

```
## [1] "Class 4"
```

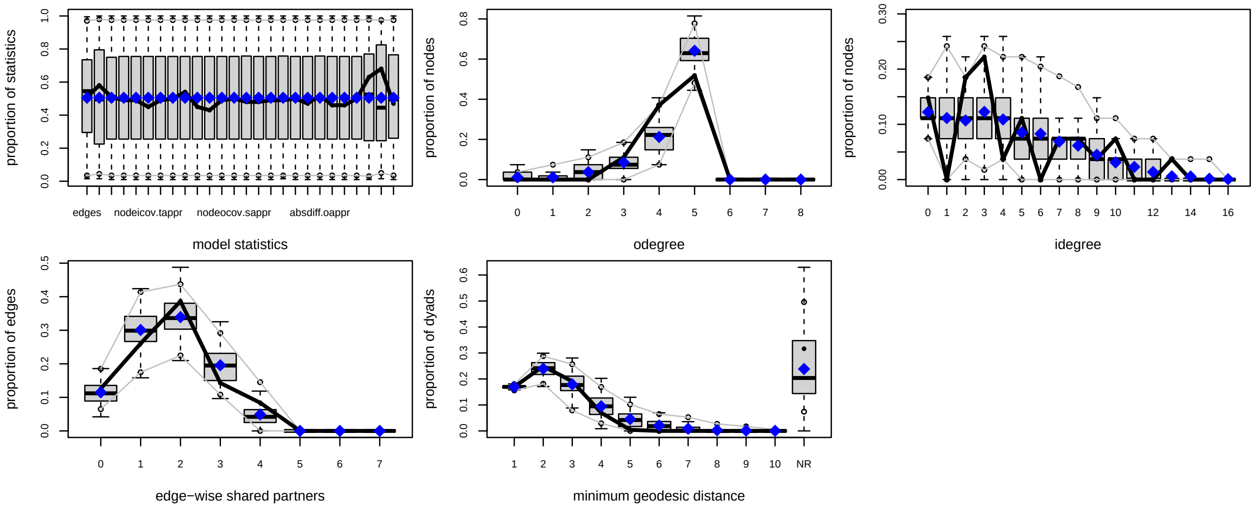


```
## [1] "Class 5"
```

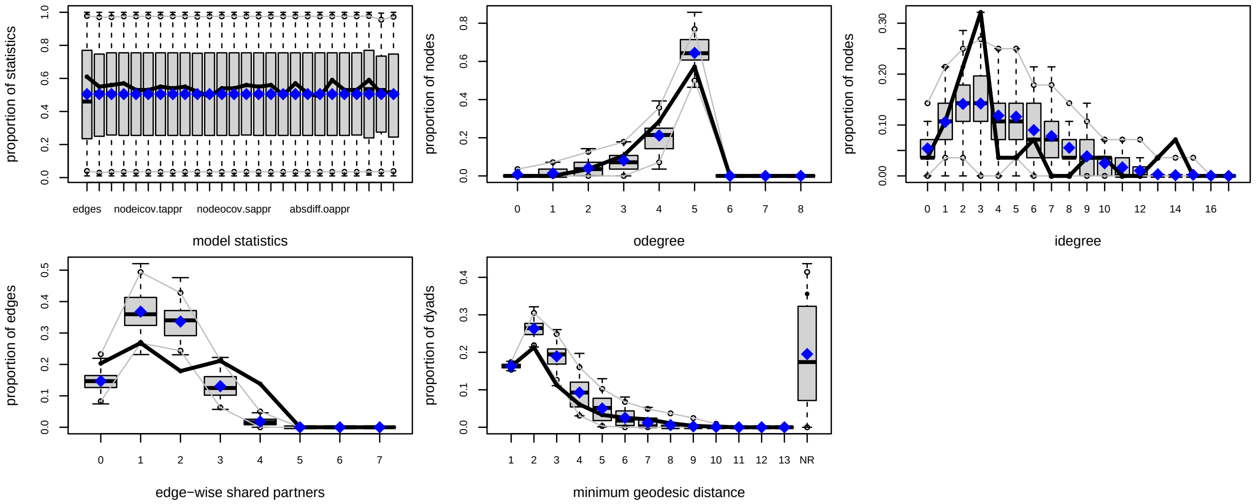


```
## [1] "Class 6"
```

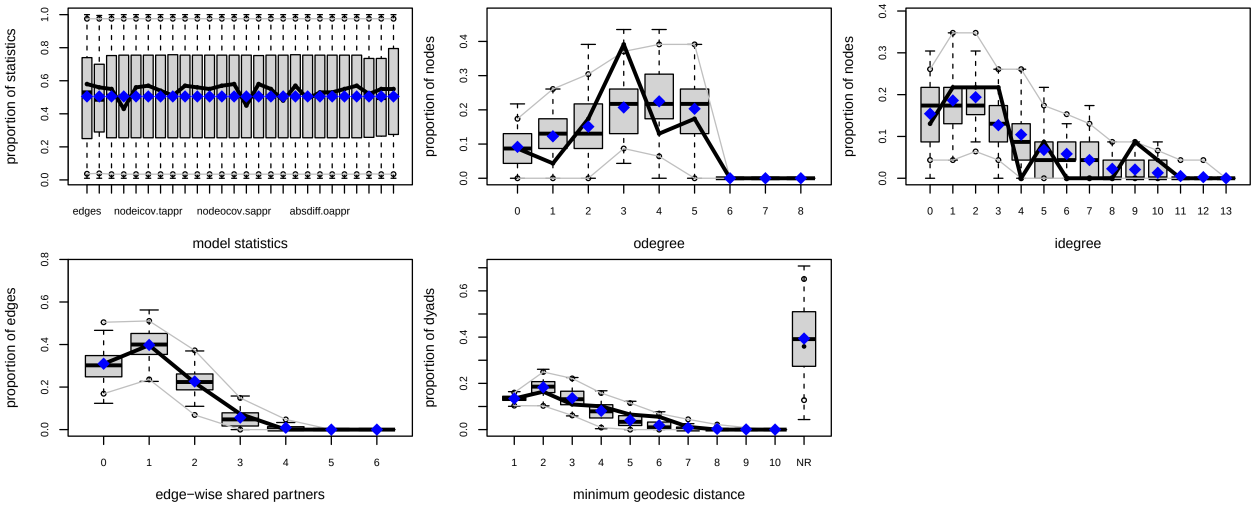

```
## [1] "Class 7"
```

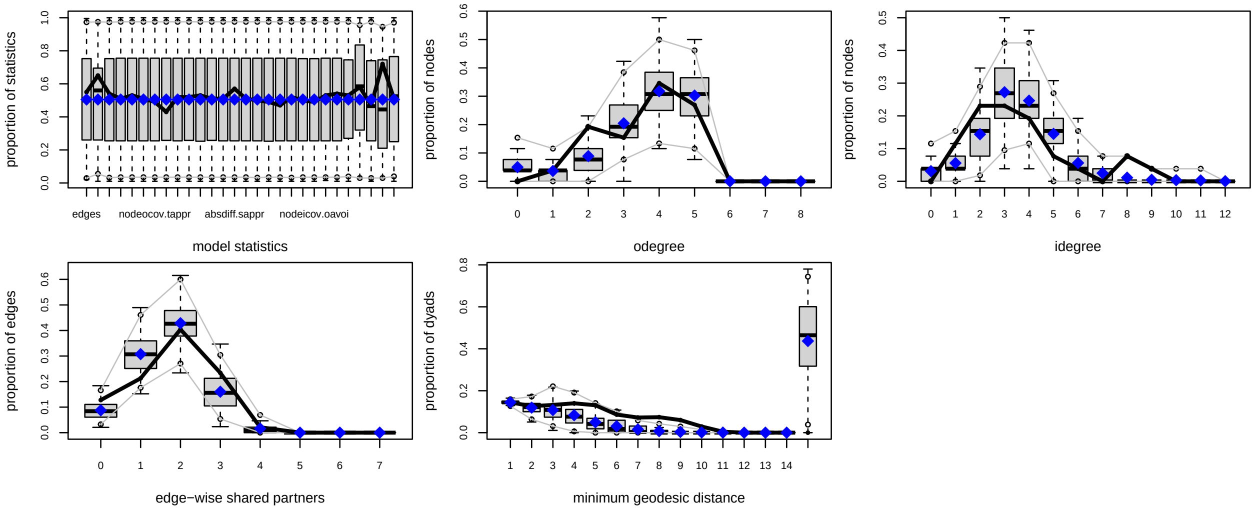


```
## [1] "Class 8"
```

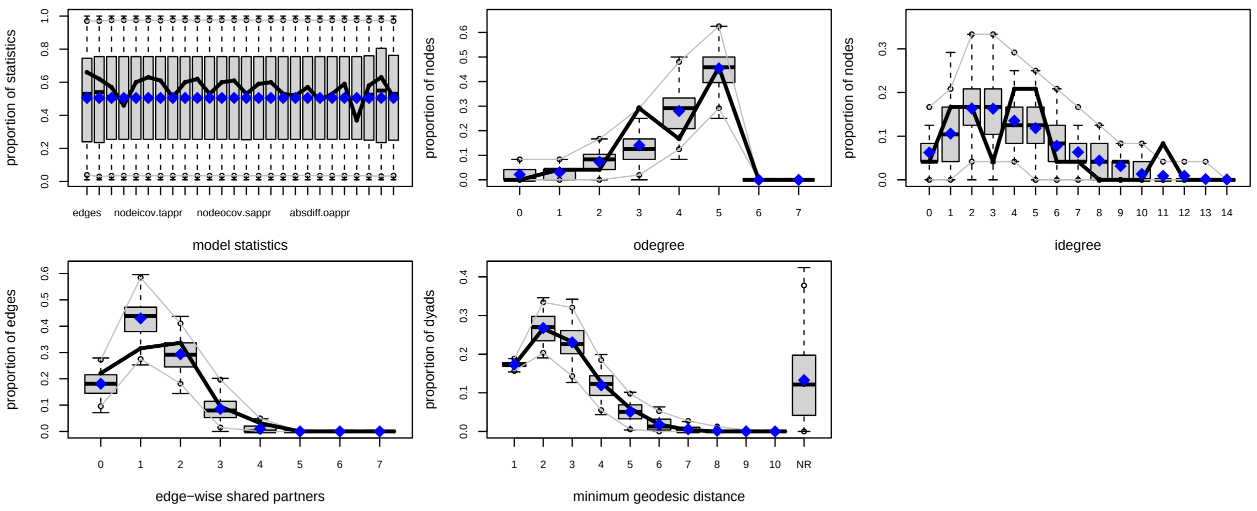


```
## [1] "Class 9"
```

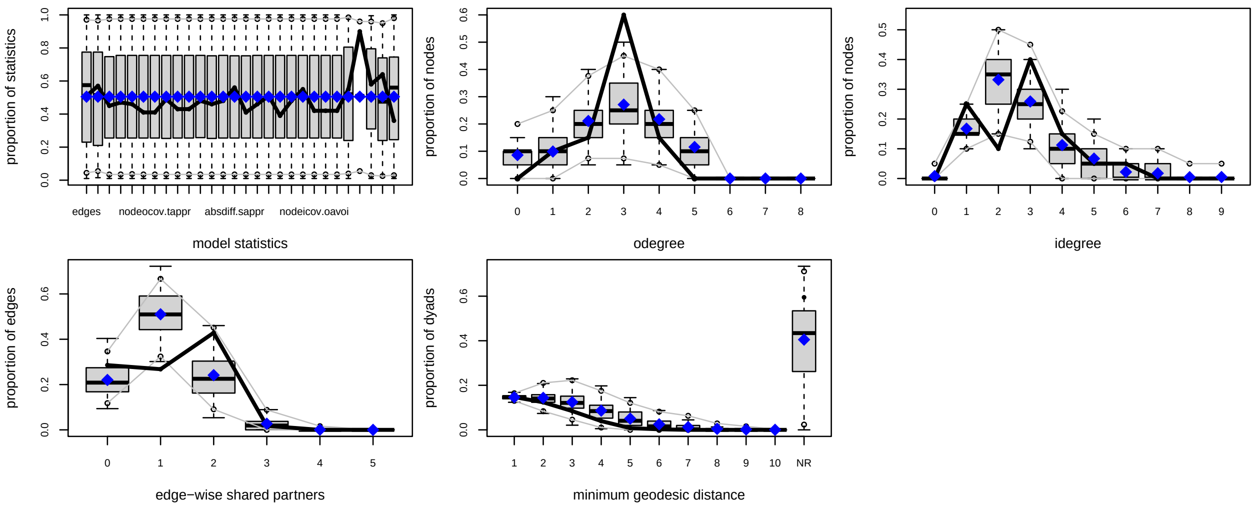

[1] "Class 10"

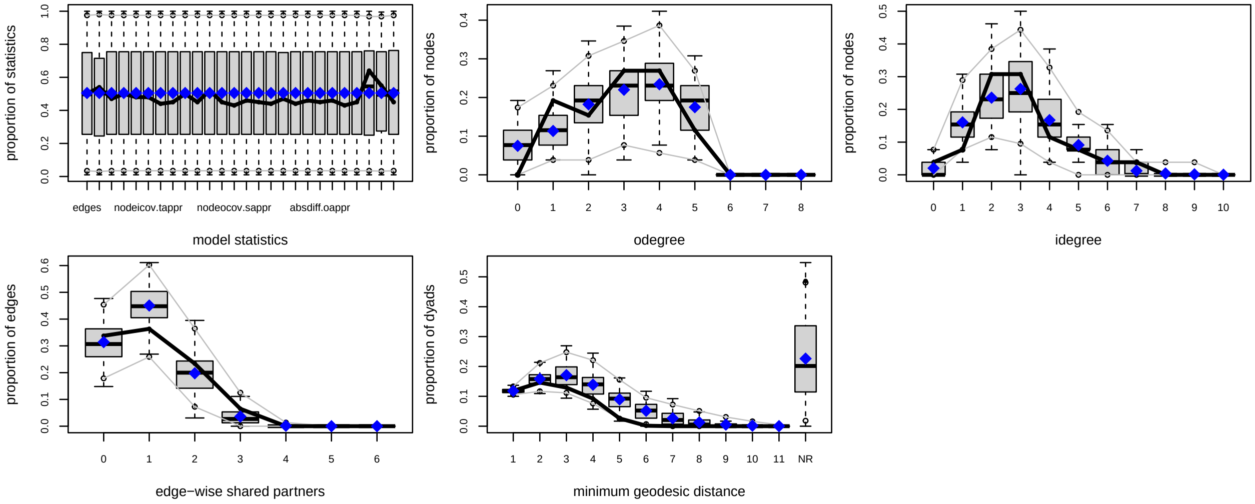

[1] "Class 11"

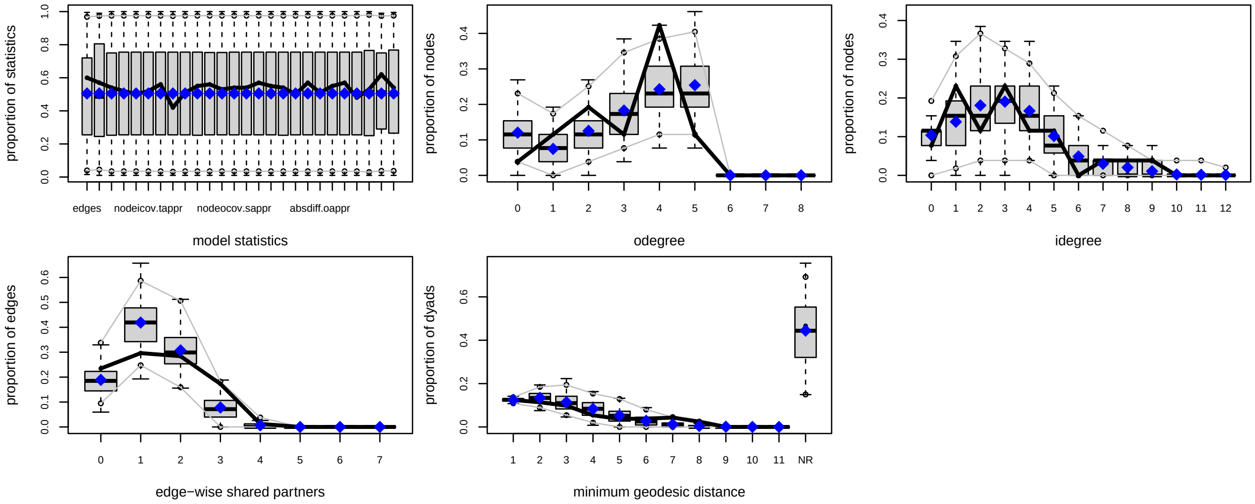

[1] "Class 12"

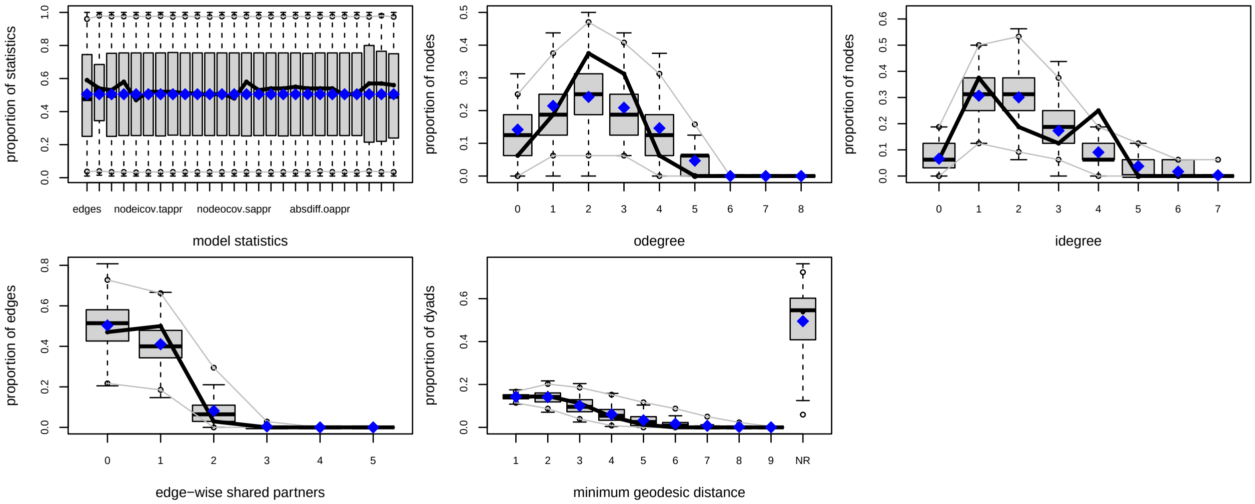

```
## [1] "Class 13"
```

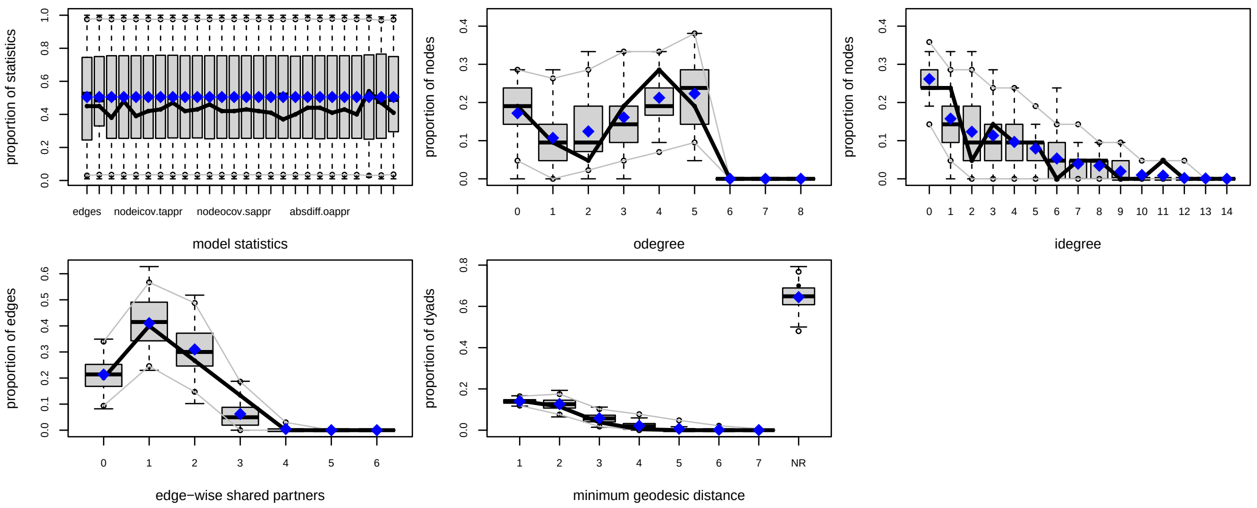


```
## [1] "Class 14"
```

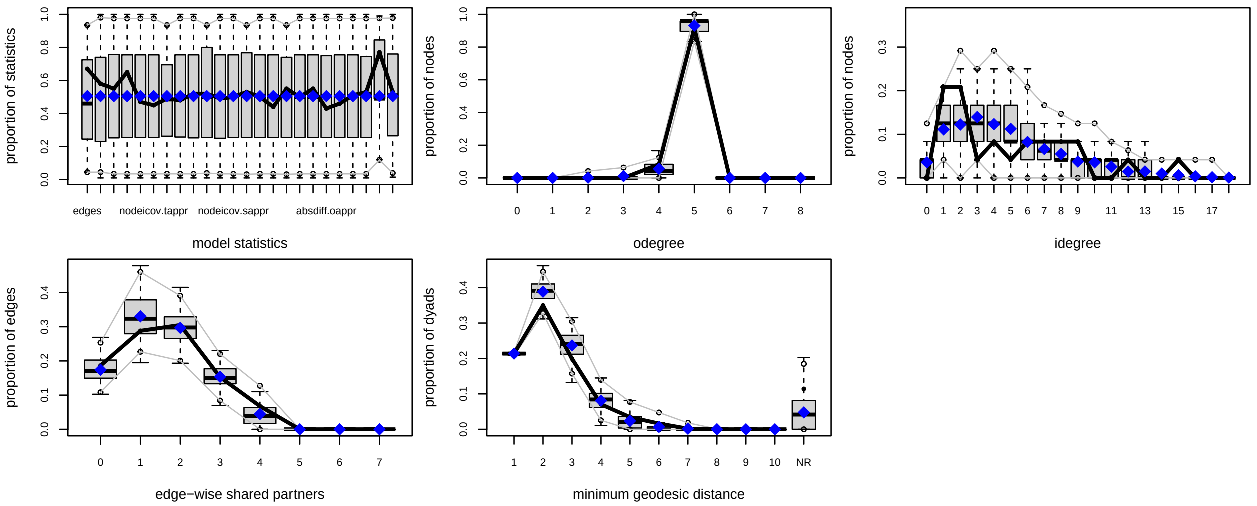


```
## [1] "Class 15"
```

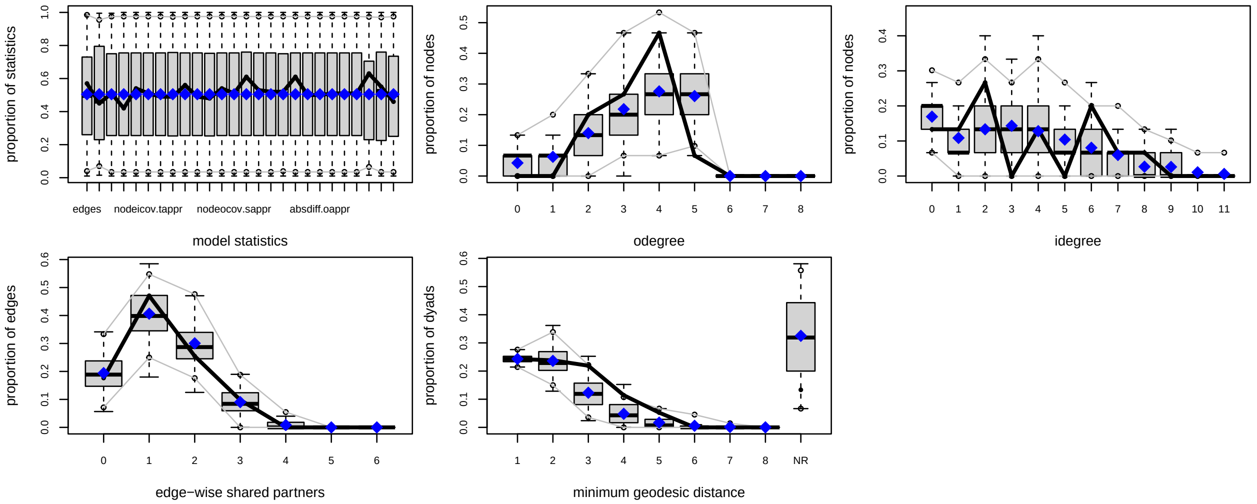

```
## [1] "Class 16"
```

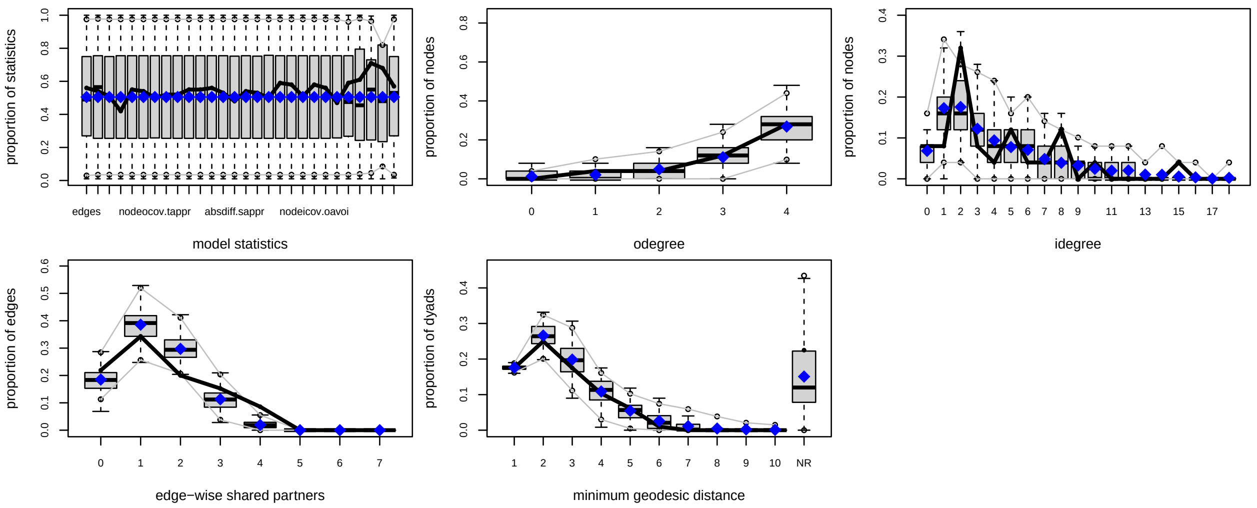
proportion of statistics
edges
nodeocov.tappr
absdiff.sappr
nodeicov.oavoi
model statistics
proportion of nodes
odegree
proportion of nodes
idegree
proportion of edges
edge−wise shared partners
proportion of dyads
minimum geodesic distance
NR

Model 3: Friendship & Gender

R Code

```
#### Model 3: Friendship and Gender ####
ergm_sp_only_friendship_list <- vector(mode = "list", length=length(SP_mn_only_list))
for(i in 1:16){
  # leave out gender sender effect for class 14
  if(i == 14){
    ergm_sp_only_friendship_list[[i]] <- ergm(SP_mn_only_list[[i]] ~
                                              edges +
                                              mutual +
                                              twopath +
                                              gwidegree(0.5,fixed=T) +
                                              gwesp(0.5,fixed=T) +
                                              edgecov(FR_mn_only_list[[i]]) +
                                              nodeifactor("gender") +
                                              # nodeofactor("gender") +
                                              nodematch("gender"),
                                            control = control.ergm(seed = 2),
                                            constraints = ~bd(maxout = 5)
    )
  }else{
    ergm_sp_only_friendship_list[[i]] <- ergm(SP_mn_only_list[[i]] ~
                                              edges +
                                              mutual +
                                              twopath +
                                              gwidegree(0.5,fixed=T) +
                                              gwesp(0.5,fixed=T) +
                                              edgecov(FR_mn_only_list[[i]]) +
                                              nodeifactor("gender") +
                                              nodeofactor("gender") +
                                              nodematch("gender"),
                                            control = control.ergm(seed = 2),
                                            constraints = ~bd(maxout = 5)
    )
  }
}

#### Meta-Analysis ####
ergm_sp_goals_gender_df <- ergm_sp_goals_gender_list %>%
  lapply(broom::tidy) %>%
  purrr::reduce(full_join,by="term") %>%
  .[,which(c(1,rep(c(1,1,0,0,0),times=16))==1)]

par_sp_goals_gender <- ergm_sp_goals_gender_df$term

sp_goals_gender_pre_meta_list <- vector(mode="list",
                                        length=length(par_sp_goals_gender)) %>%
  `names<-`(par_sp_goals_gender)
for(i in 1:length(par_sp_goals_gender)){
  sp_goals_gender_pre_meta_list[[i]] <-
    ergm_sp_goals_gender_df[i,2:ncol(ergm_sp_goals_gender_df)] %>%
    as.numeric %>%
```

```r
    matrix(ncol=2,byrow=TRUE) %>%
    as.data.frame %>%
    `colnames<-`(c("coef","se"))
}

sp_goals_gender_ma_list <- vector(mode = "list",
                                  length=length(par_sp_goals_gender)) %>%
  `names<-`(par_sp_goals_gender)
for(i in 1:length(par_sp_goals_gender)){
  sp_goals_gender_ma_list[[i]] <-
    rma(yi=coef,sei=se,data=sp_goals_gender_pre_meta_list[[i]],
        control = list(stepadj=0.5,maxiter=1000))
}

# summary table
lapply(sp_goals_gender_ma_list,broom::tidy) %>%
  purrr::reduce(rbind) %>%
  cbind(par_sp_goals_gender,.) %>%
  mutate(estimate = round(estimate,4),
         std.error = round(std.error,4),
         statistic = round(statistic,4),
         p.value = round(p.value,4)) %>%
  select(c(1,4,5,7))
```

Goodness of Fit Plots

```
## [1] "Class 1"
```

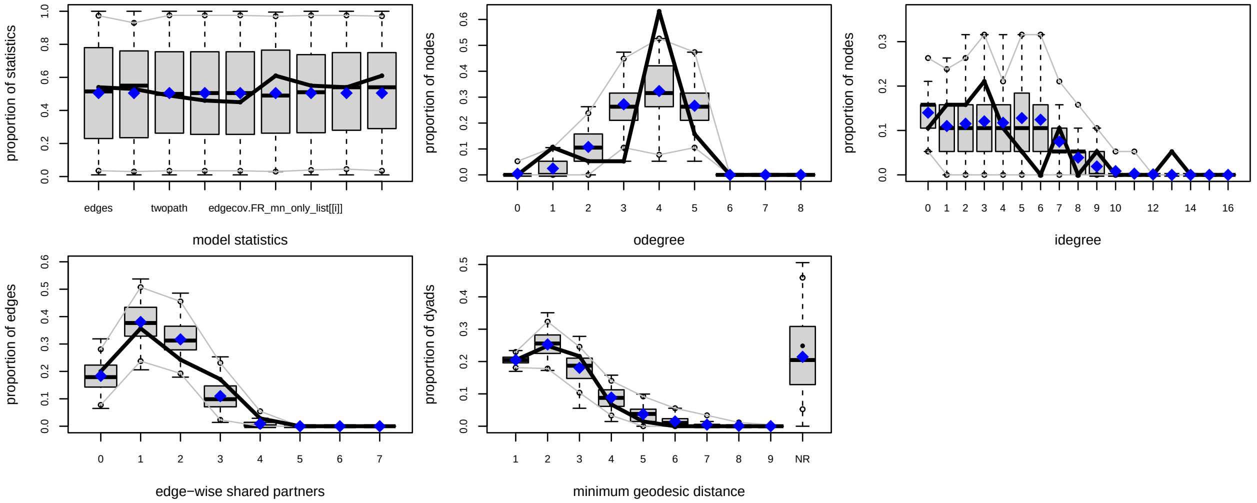


```
## [1] "Class 2"
```

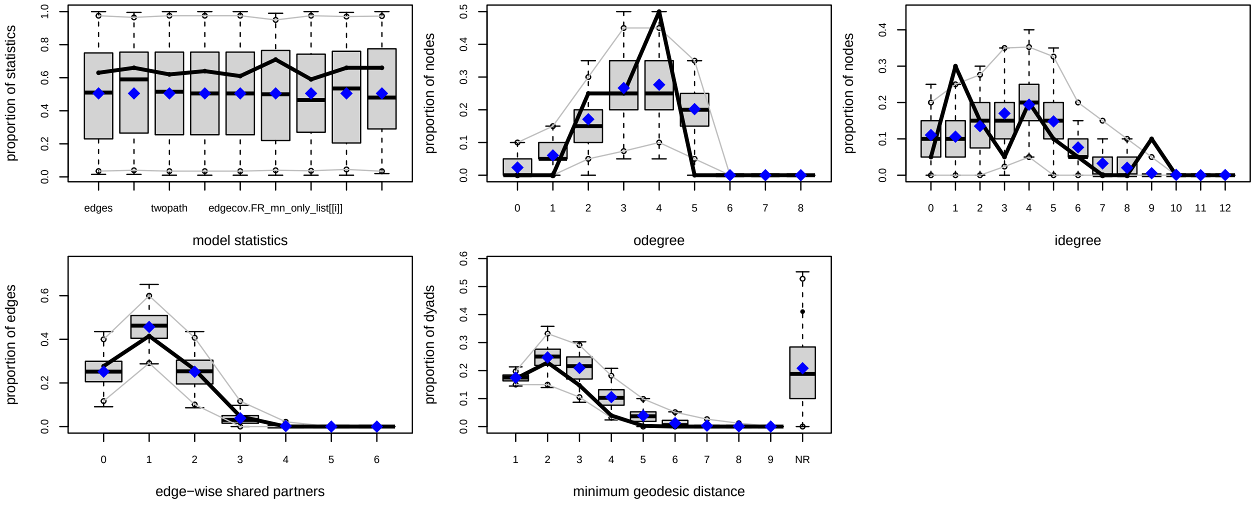


```
## [1] "Class 3"
```

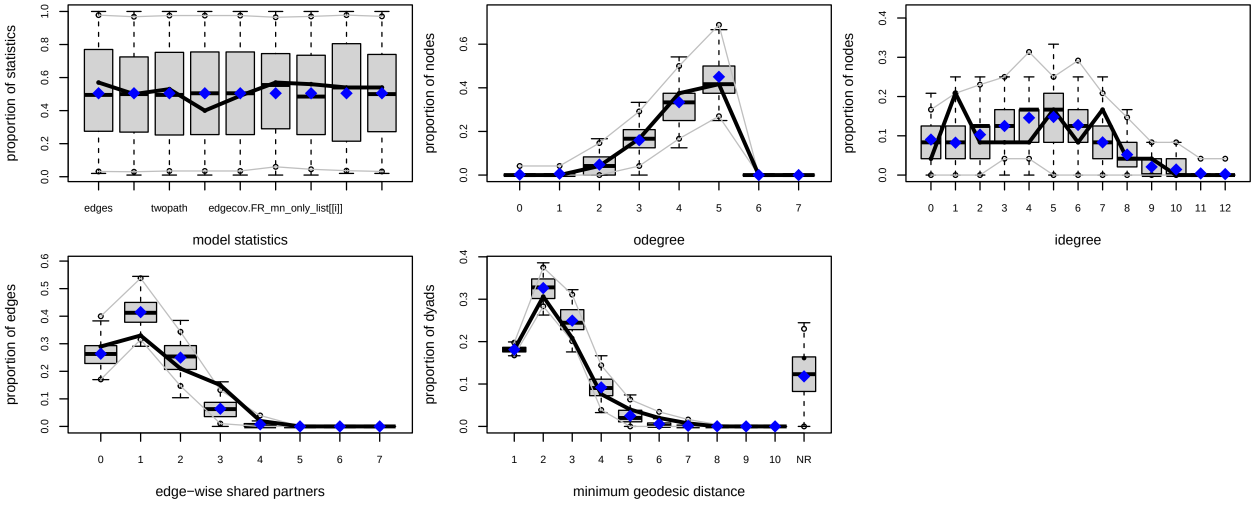

```
## [1] "Class 4"
```

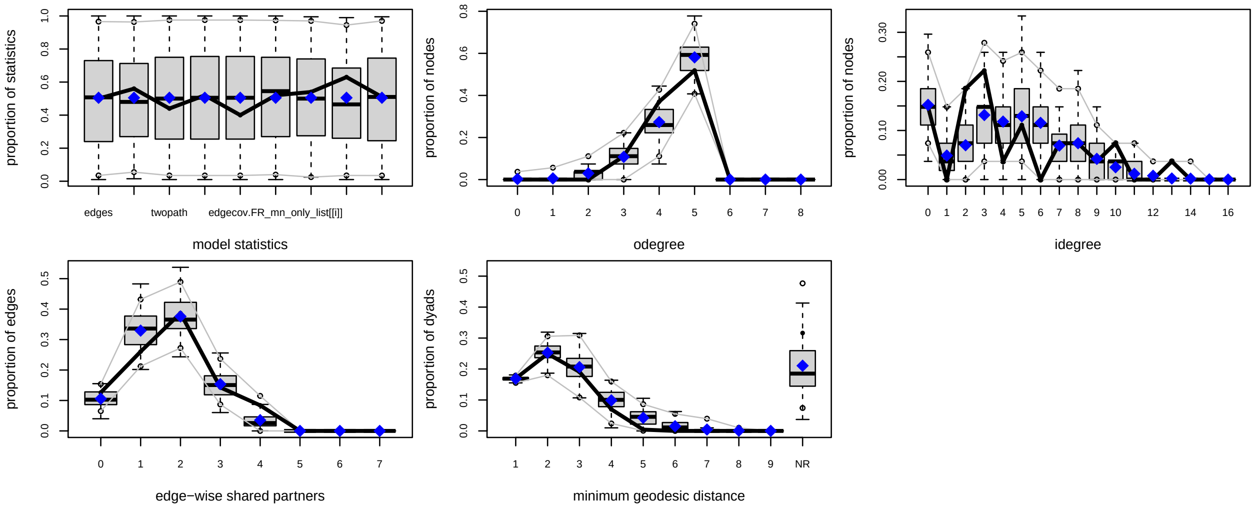


```
## [1] "Class 5"
```

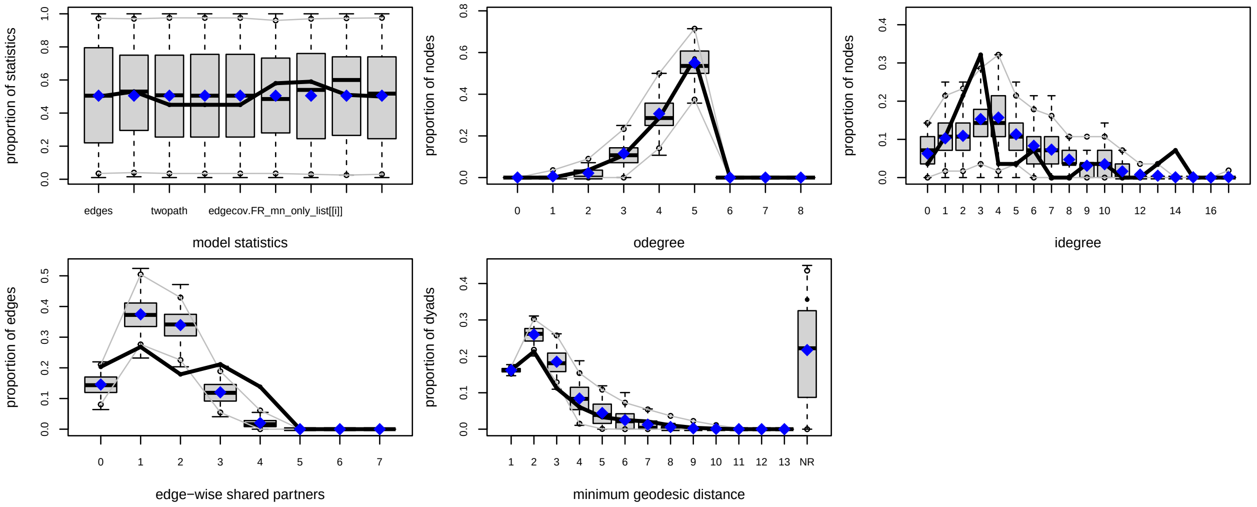


```
## [1] "Class 6"
```

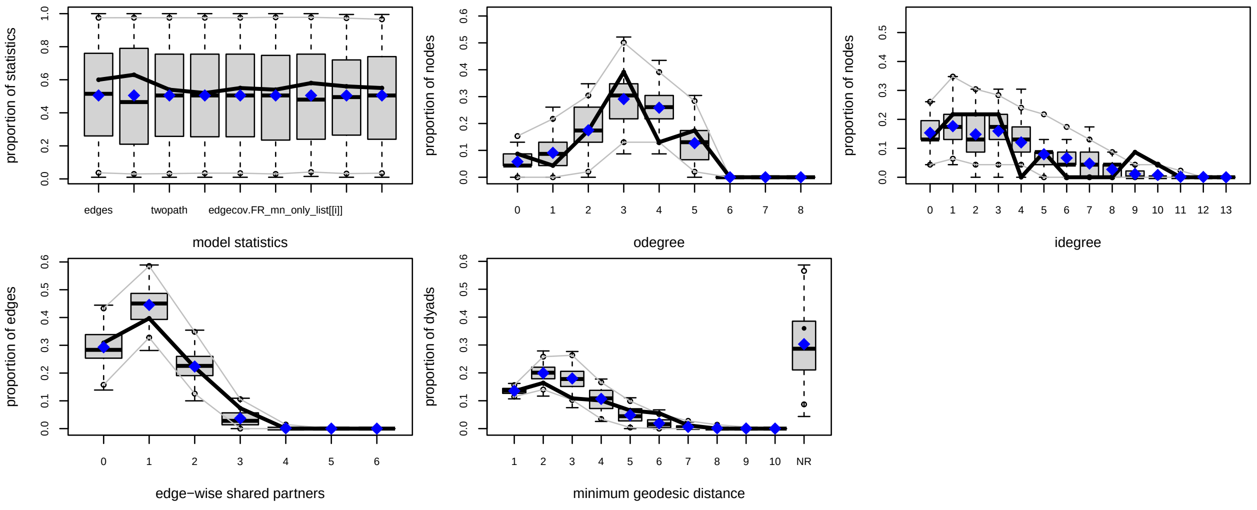

```
## [1] "Class 7"
```

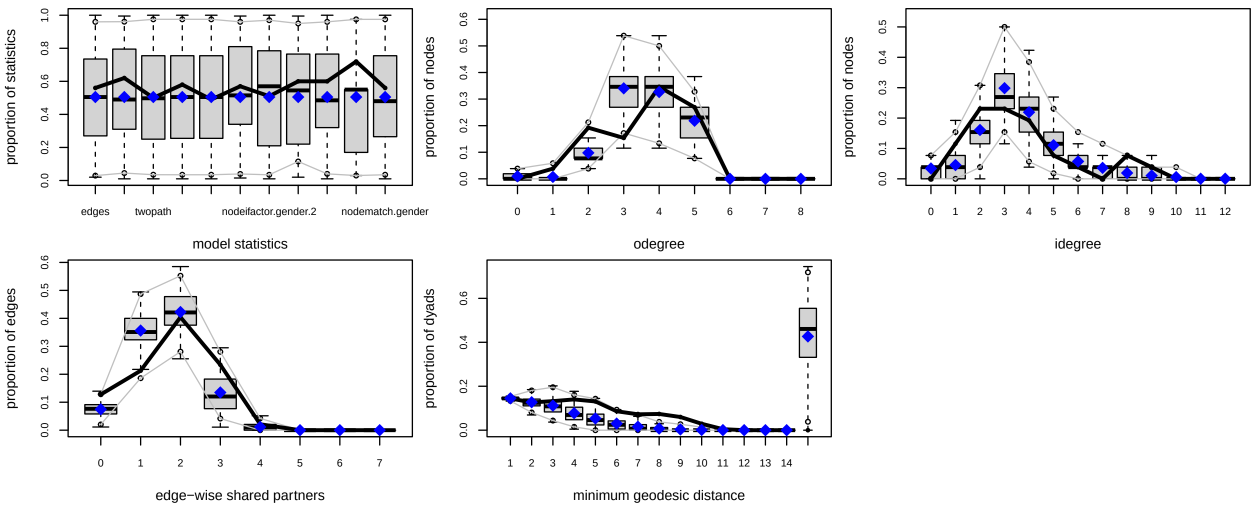


```
## [1] "Class 8"
```

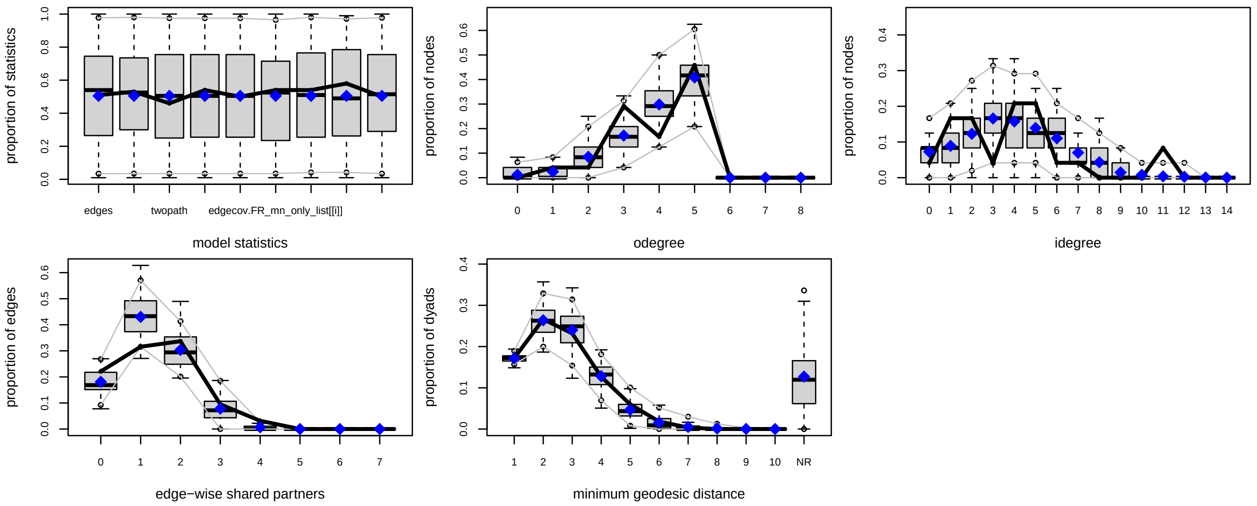


```
## [1] "Class 9"
```

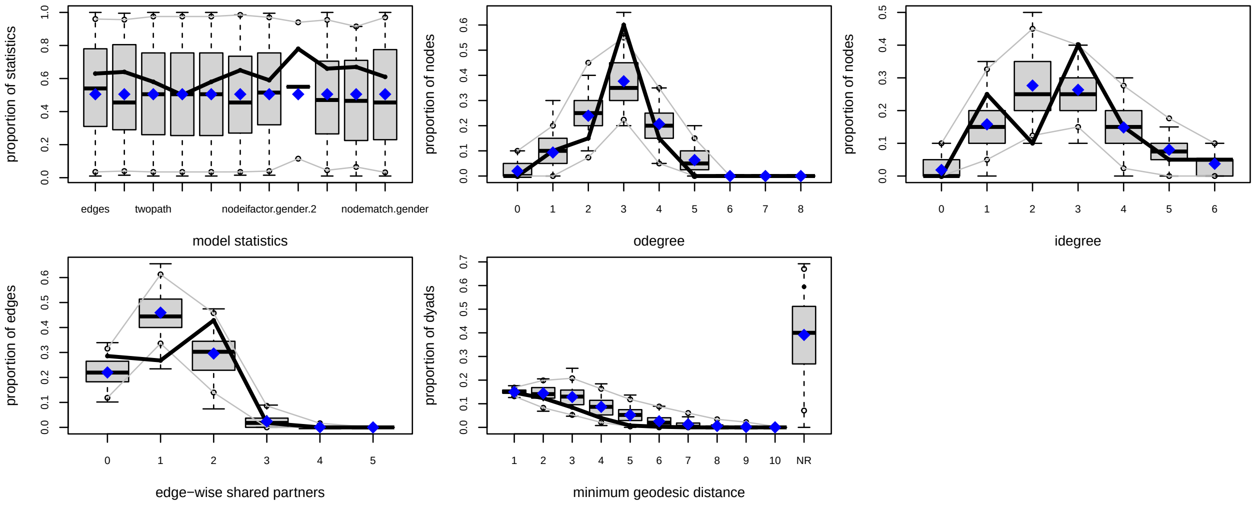

[1] "Class 10"

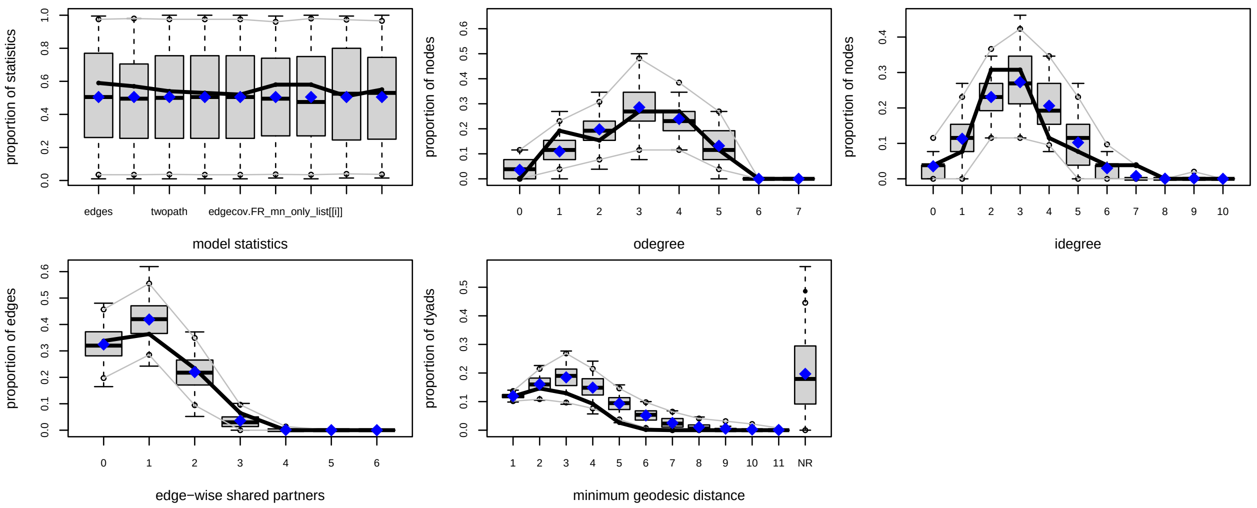

[1] "Class 11"

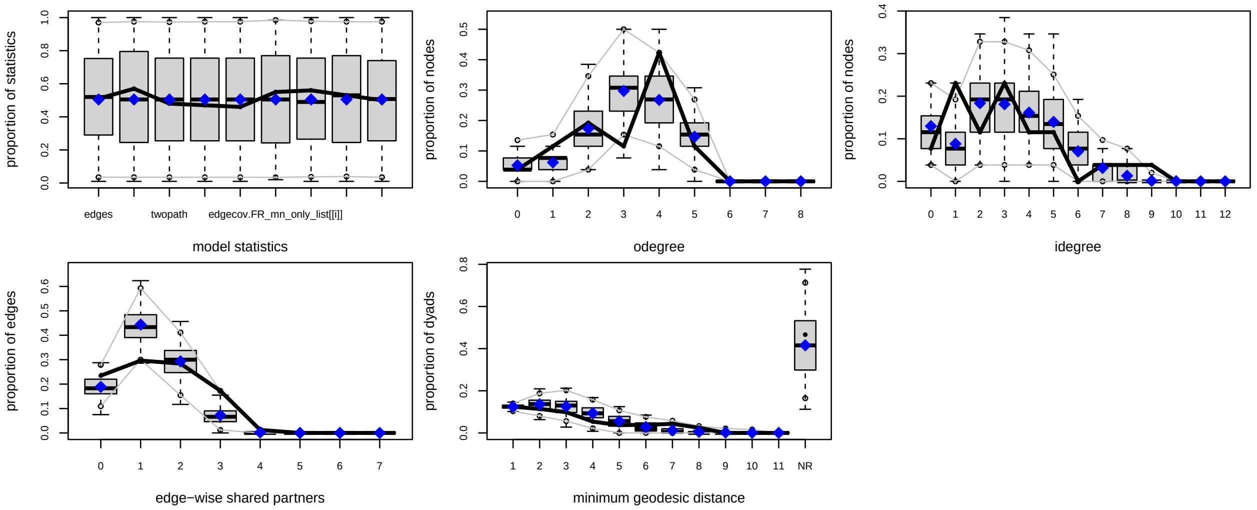

[1] "Class 12"

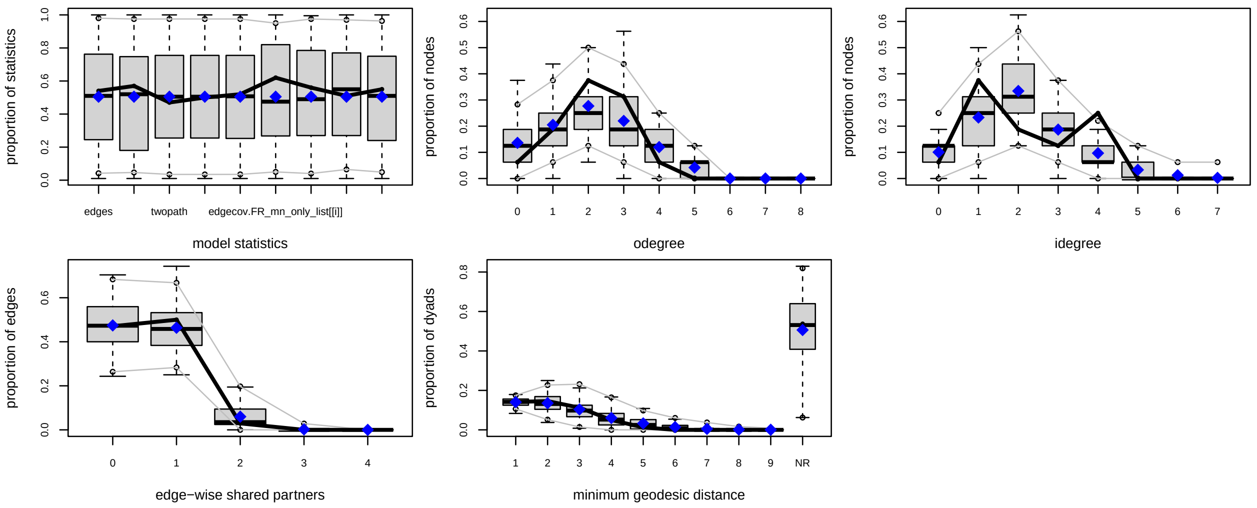

```
## [1] "Class 13"
```

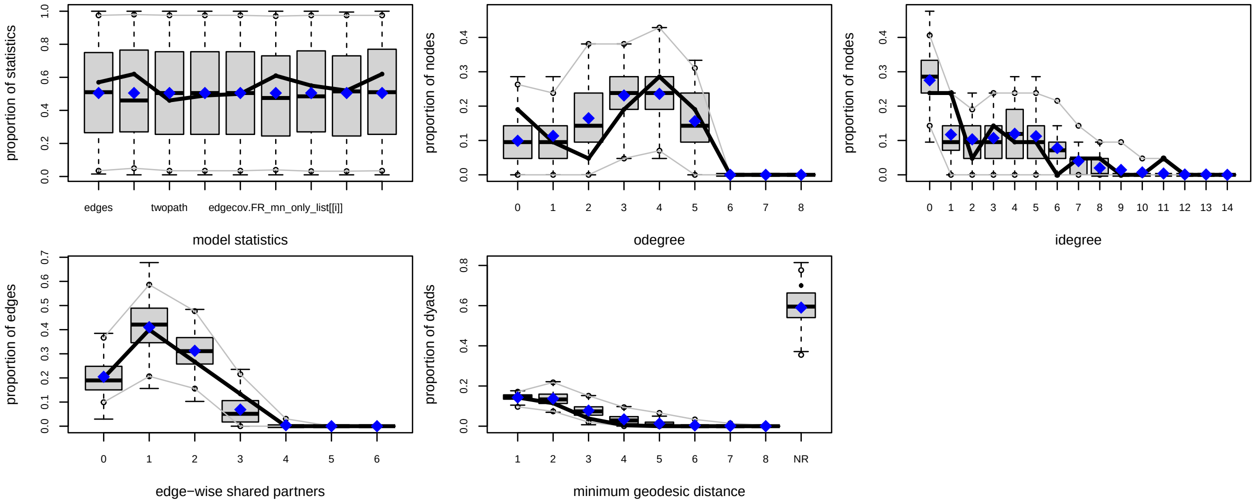


```
## [1] "Class 14"
```

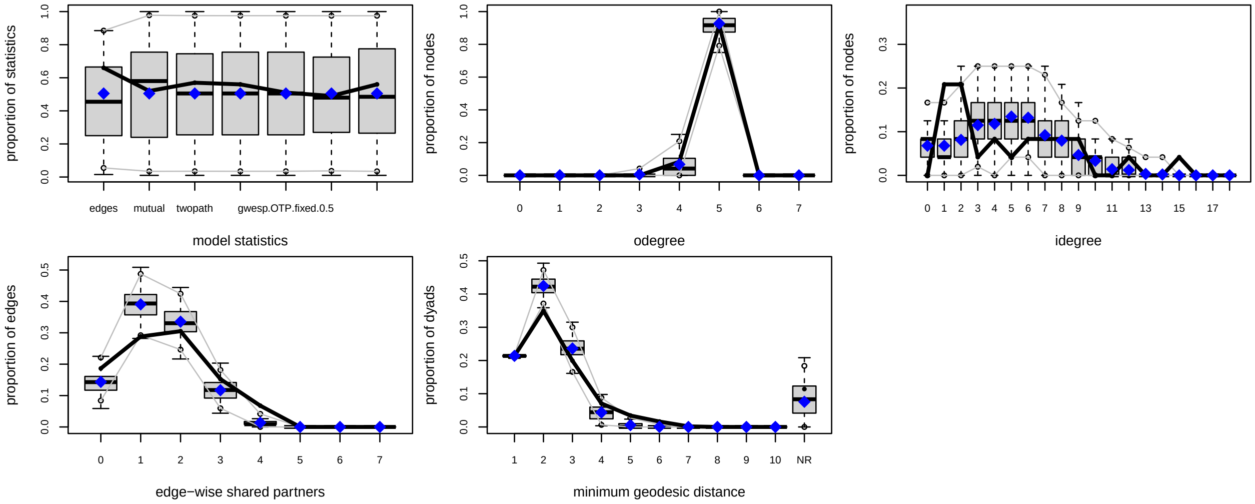


```
## [1] "Class 15"
```

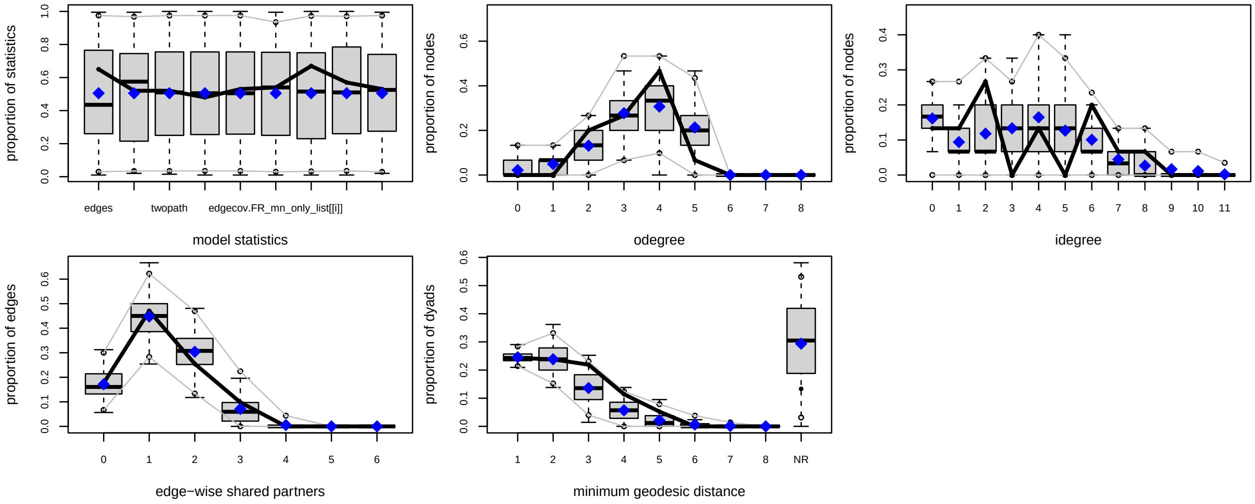

```
## [1] "Class 16"
```

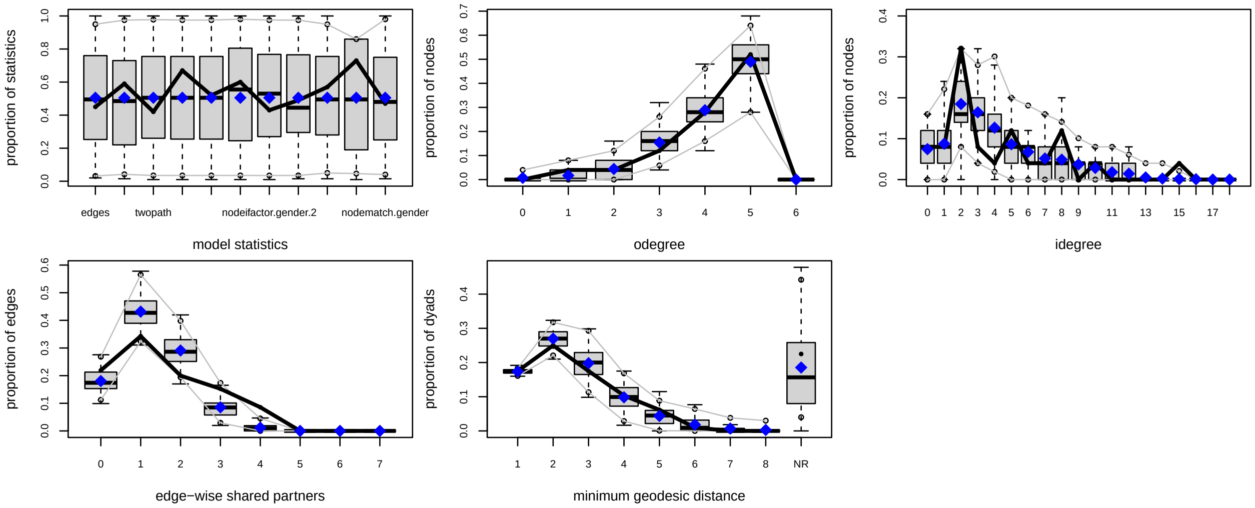

proportion of statistics
edges
twopath
nodeifactor.gender.2
nodematch.gender
model statistics
proportion of nodes
odegree
proportion of nodes
idegree
proportion of edges
edge−wise shared partners
proportion of dyads
minimum geodesic distance
NR

Model 4: Full Model

R Code

```
#### Model 4: Full Model ####
ergm_sp_full_list <- vector(mode = "list", length=length(SP_mn_only_list))
for(i in 1:16){
  # leave out other approach & avoidance as well as gender sender effect for class 14
  if(i == 14){
    ergm_sp_full_list[[i]] <- ergm(SP_mn_only_list[[i]] ~
                                     edges +
                                     mutual +
                                     twopath +
                                     gwidegree(0.5,fixed=T) +
                                     gwesp(0.5,fixed=T) +
                                     nodeicov("tappr") +
                                     nodeocov("tappr") +
                                     absdiff("tappr") +
                                     nodeicov("tavoi") +
                                     nodeocov("tavoi") +
                                     absdiff("tavoi") +
                                     nodeicov("sappr") +
                                     nodeocov("sappr") +
                                     absdiff("sappr") +
                                     nodeicov("savoi") +
                                     nodeocov("savoi") +
                                     absdiff("savoi") +
                                     nodeicov("oappr") +
                                     #nodeocov("oappr") +
                                     absdiff("oappr") +
                                     nodeicov("oavoi") +
                                     #nodeocov("oavoi") +
                                     absdiff("oavoi") +
                                     edgecov(FR_mn_only_list[[i]]) +
                                     nodeifactor("gender") +
                                     #nodeofactor("gender") +
                                     nodematch("gender"),
                                   control = control.ergm(seed = 1),
                                   constraints = ~bd(maxout = 5)
    )
  }else{
    ergm_sp_full_list[[i]] <- ergm(SP_mn_only_list[[i]] ~
                                     edges +
                                     mutual +
                                     twopath +
                                     gwidegree(0.5,fixed=T) +
                                     gwesp(0.5,fixed=T) +
                                     nodeicov("tappr") +
                                     nodeocov("tappr") +
                                     absdiff("tappr") +
                                     nodeicov("tavoi") +
                                     nodeocov("tavoi") +
                                     absdiff("tavoi") +
                                     nodeicov("sappr") +
```

```r
                                          nodeocov("sappr") +
                                          absdiff("sappr") +
                                          nodeicov("savoi") +
                                          nodeocov("savoi") +
                                          absdiff("savoi") +
                                          nodeicov("oappr") +
                                          nodeocov("oappr") +
                                          absdiff("oappr") +
                                          nodeicov("oavoi") +
                                          nodeocov("oavoi") +
                                          absdiff("oavoi") +
                                          edgecov(FR_mn_only_list[[i]]) +
                                          nodeifactor("gender") +
                                          nodeofactor("gender") +
                                          nodematch("gender"),
                                        control = control.ergm(seed = 1),
                                        constraints = ~bd(maxout = 5)
    )
  }
}

#### Meta-Analysis ####
ergm_sp_fullmodel_df <- ergm_sp_full_list %>%
  lapply(broom::tidy) %>%
  purrr::reduce(full_join,by="term") %>%
  .[,which(c(1,rep(c(1,1,0,0,0),times=16))==1)]

par_sp_fullmodel <- ergm_sp_fullmodel_df$term

sp_fullmodel_pre_meta_list <- vector(mode="list",length=length(par_sp_fullmodel)) %>%
  `names<-`(par_sp_fullmodel)
for(i in 1:length(par_sp_fullmodel)){
  sp_fullmodel_pre_meta_list[[i]] <-
    ergm_sp_fullmodel_df[i,2:ncol(ergm_sp_fullmodel_df)] %>%
    as.numeric %>%
    matrix(ncol=2,byrow=TRUE) %>%
    as.data.frame %>%
    `colnames<-`(c("coef","se"))
}

sp_fullmodel_ma_list <- vector(mode = "list", length=length(par_sp_fullmodel)) %>%
  `names<-`(par_sp_fullmodel)
for(i in 1:length(par_sp_fullmodel)){
  sp_fullmodel_ma_list[[i]] <-
    rma(yi=coef,sei=se,data=sp_fullmodel_pre_meta_list[[i]],
        control = list(stepadj=0.5,maxiter=1000))
}

# summary table
lapply(sp_fullmodel_ma_list,broom::tidy) %>%
  purrr::reduce(rbind) %>%
  cbind(par_sp_fullmodel,.) %>%
  mutate(estimate = round(estimate,4),
```

```
         std.error = round(std.error,4),
         statistic = round(statistic,4),
         p.value = round(p.value,4)) %>%
  select(c(1,4,5,7))
```

Goodness of Fit Plots

```
## [1] "Class 1"
```

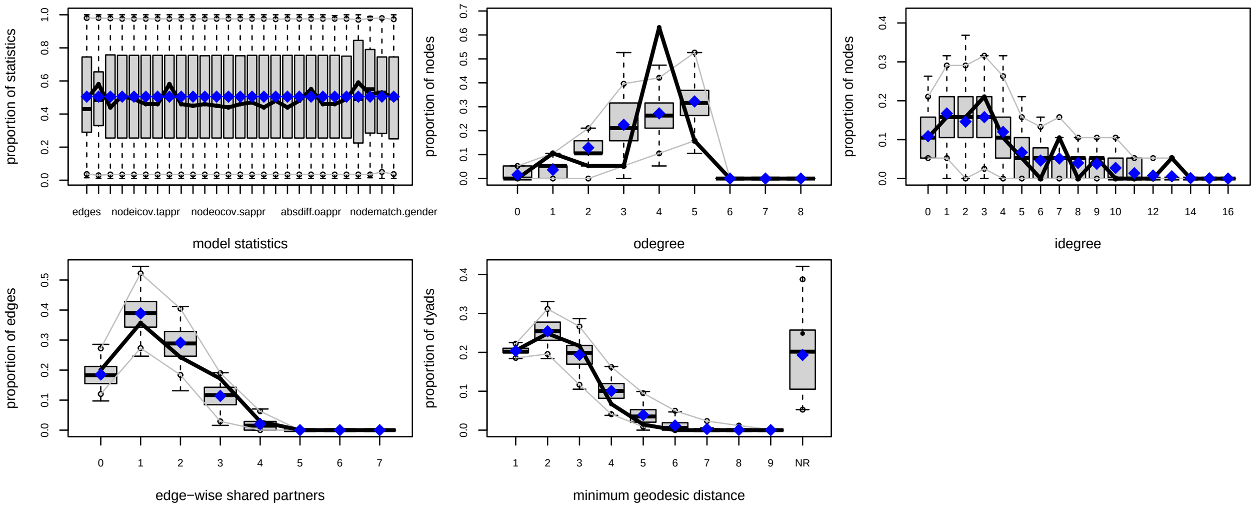


```
## [1] "Class 2"
```

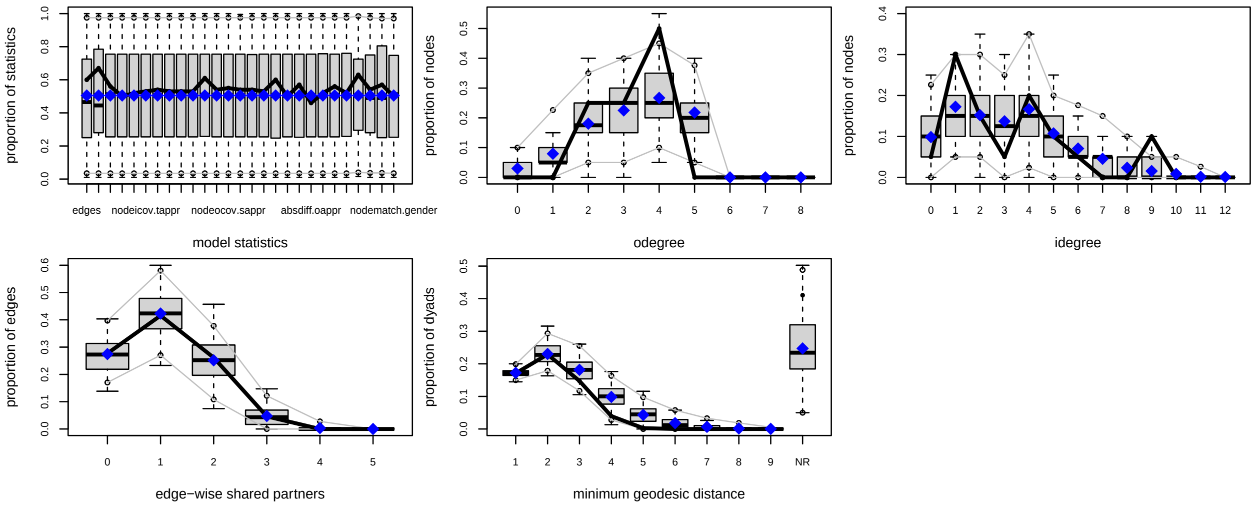


```
## [1] "Class 3"
```

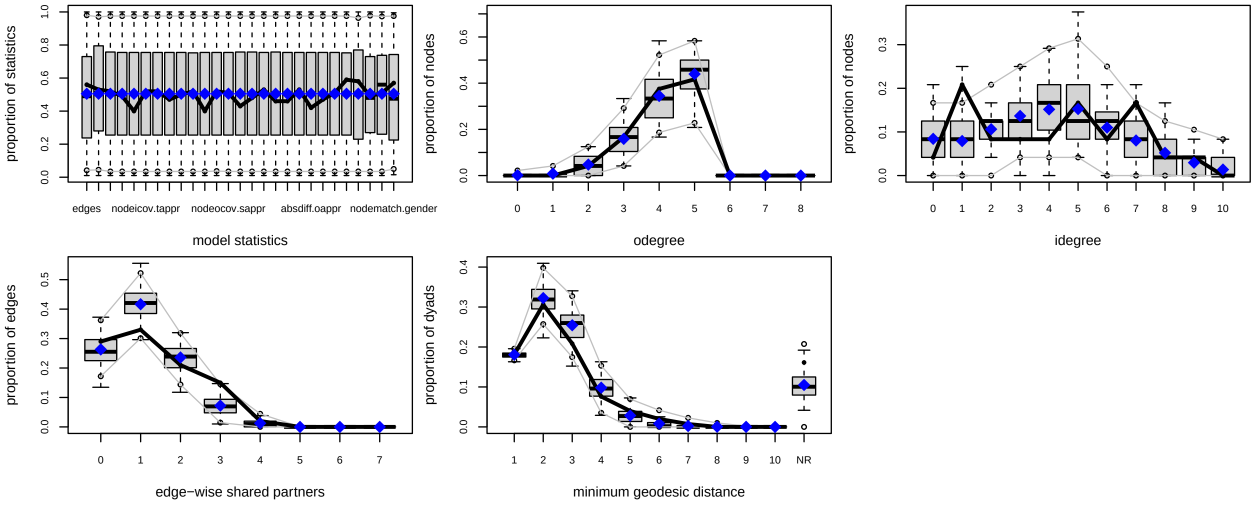

```
## [1] "Class 4"
```

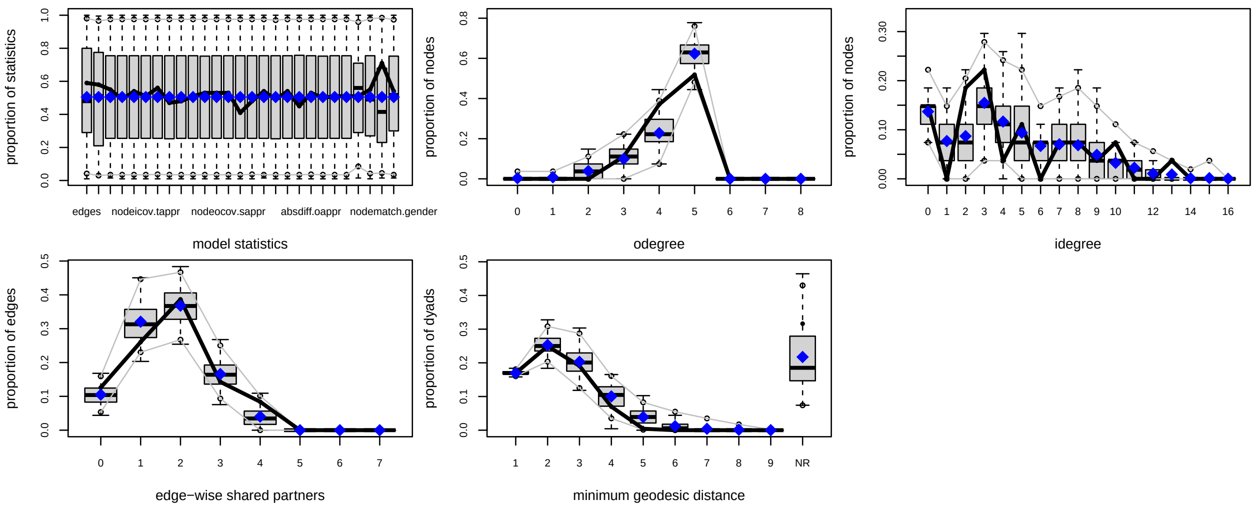


```
## [1] "Class 5"
```

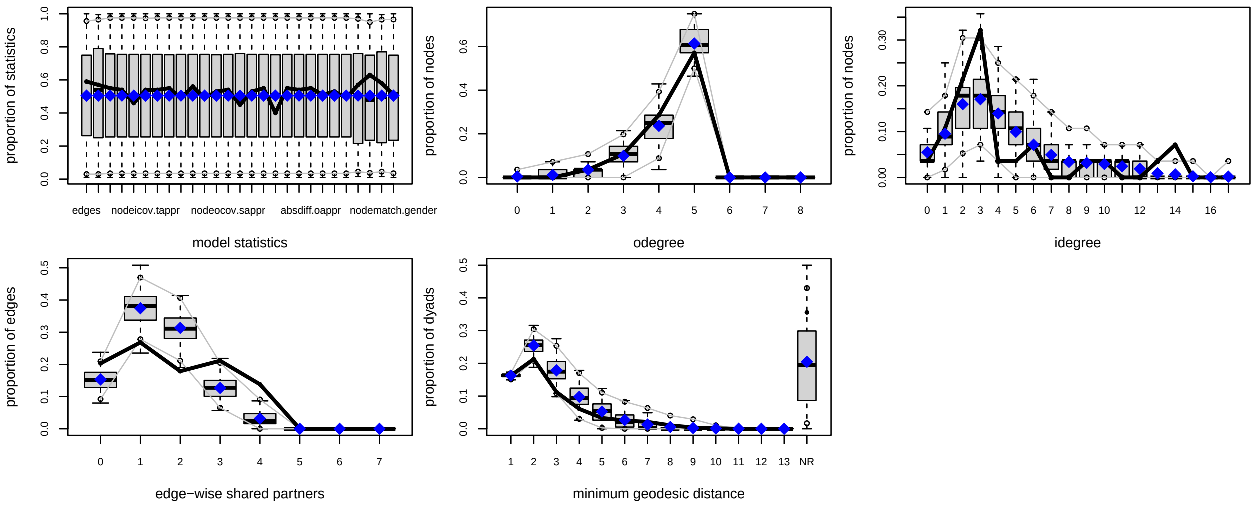


```
## [1] "Class 6"
```

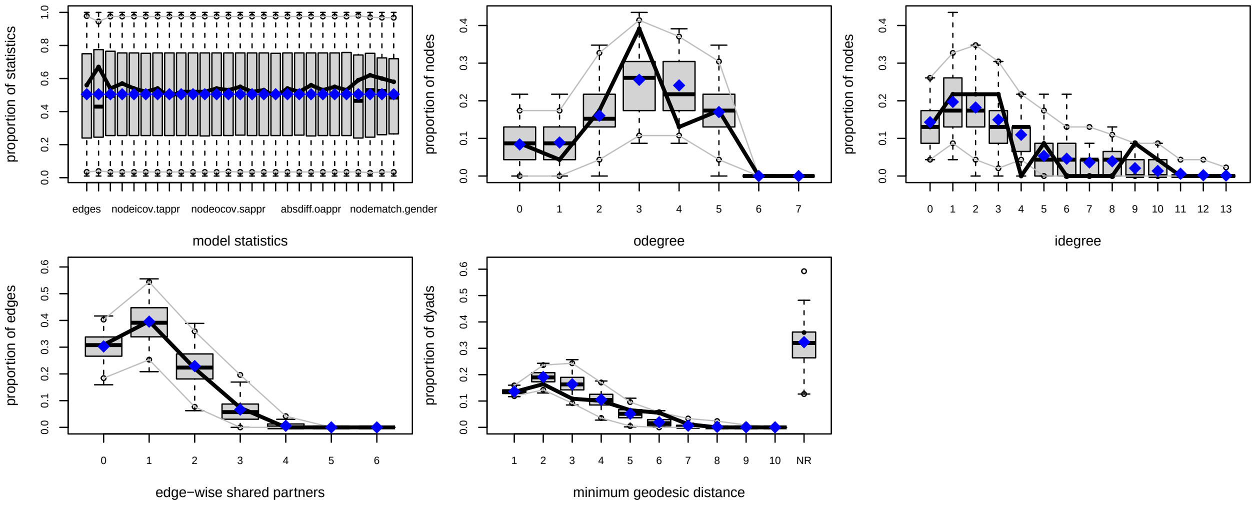

[1] "Class 7"

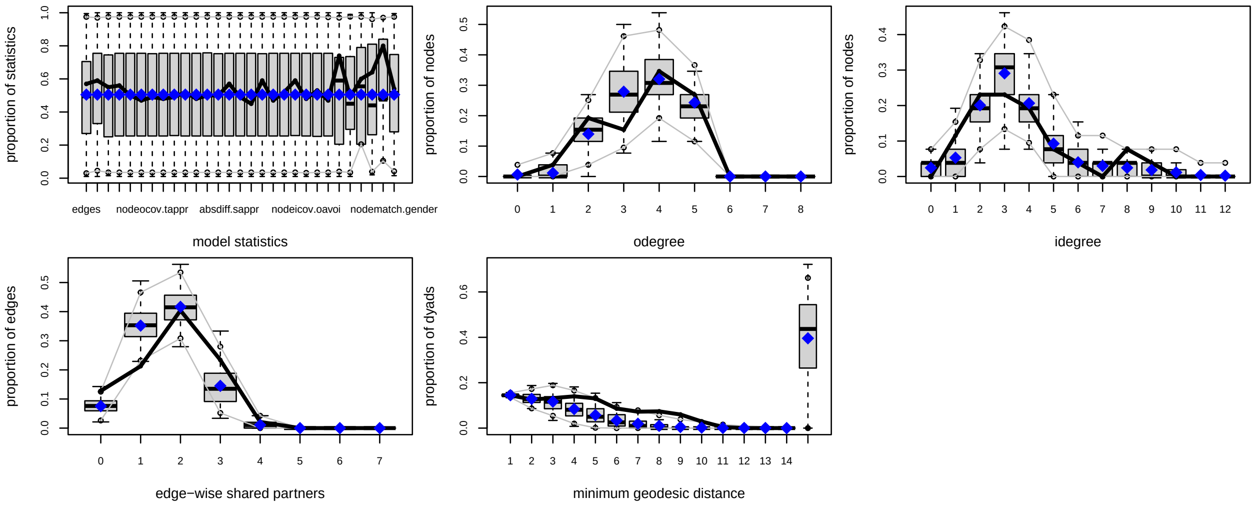

[1] "Class 8"

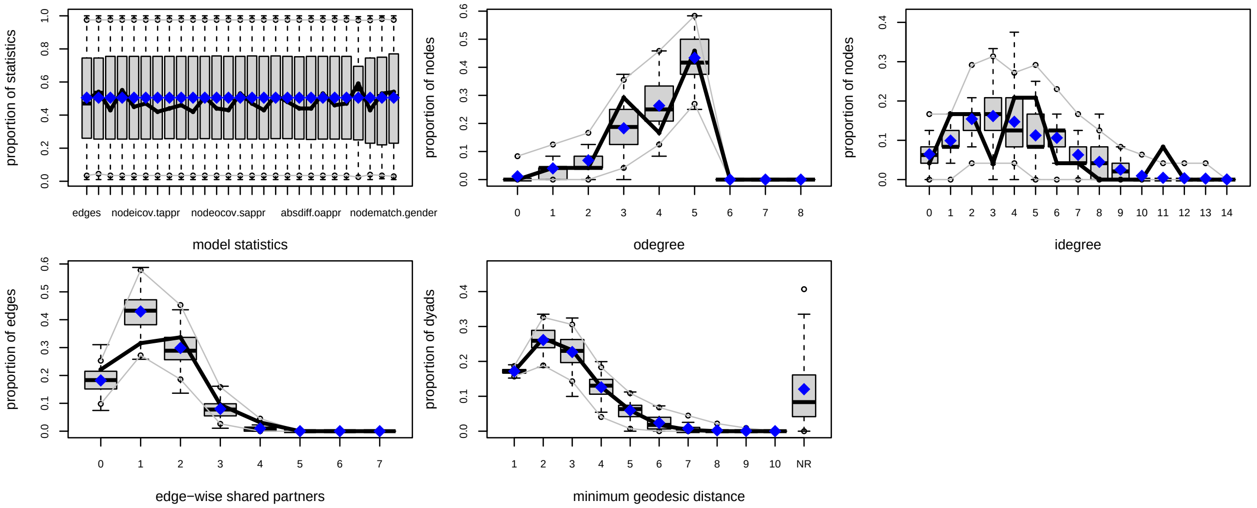

[1] "Class 9"

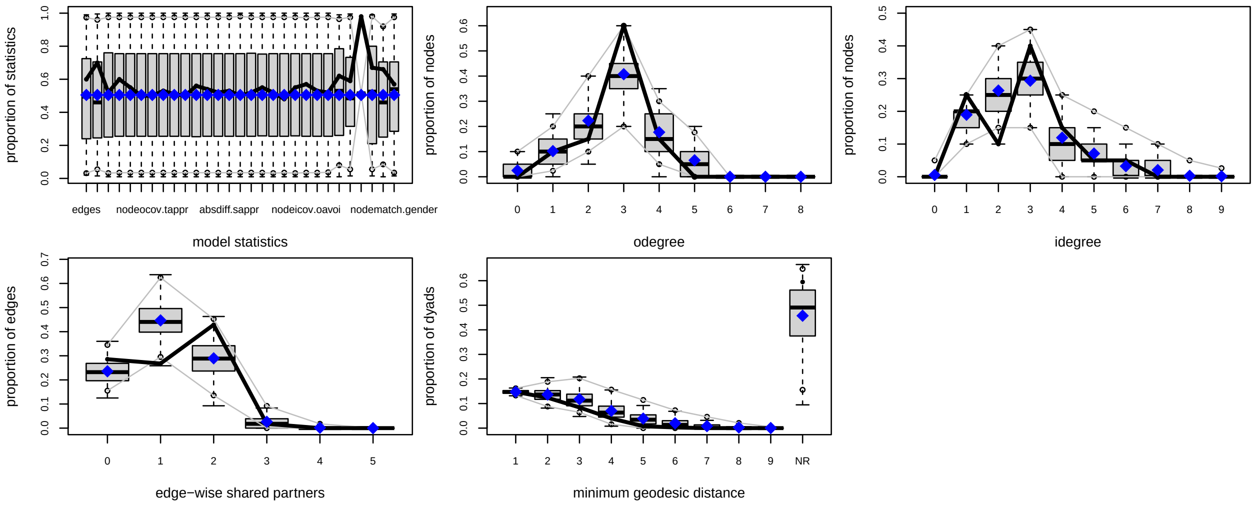

```
## [1] "Class 10"
```

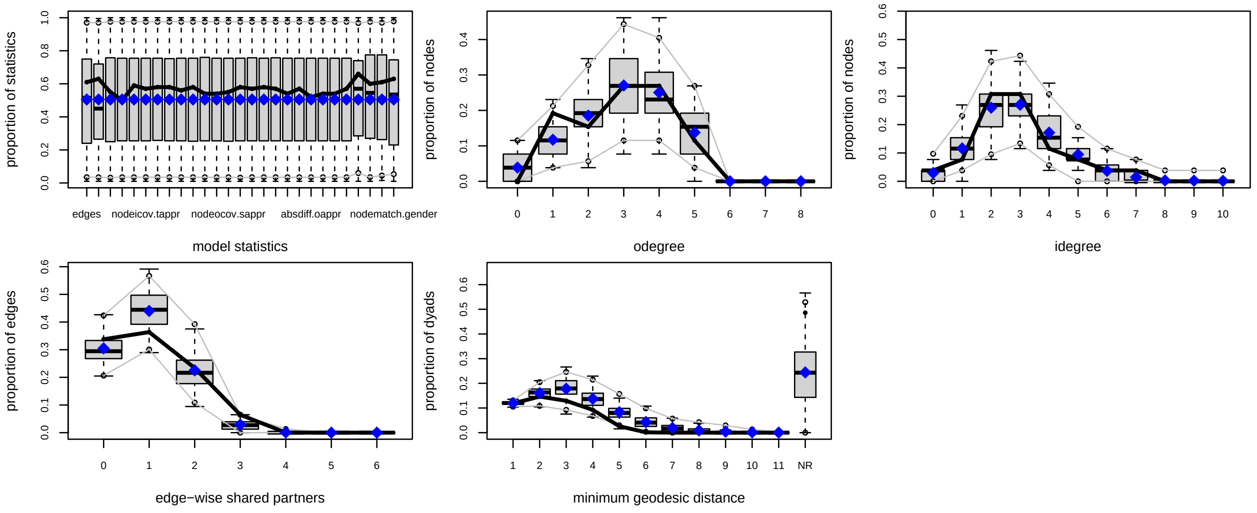


```
## [1] "Class 11"
```

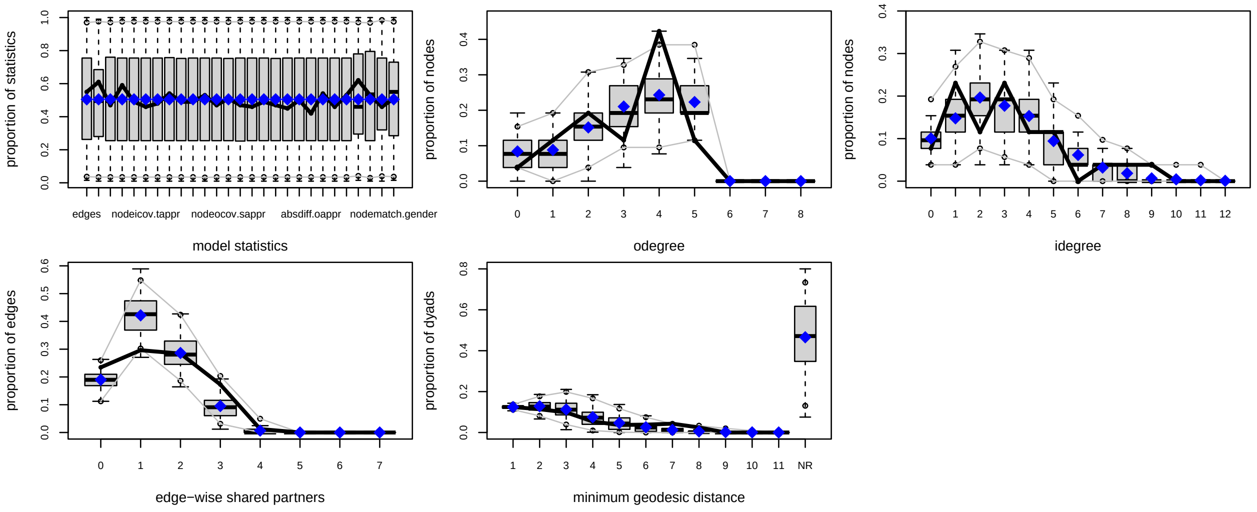


```
## [1] "Class 12"
```

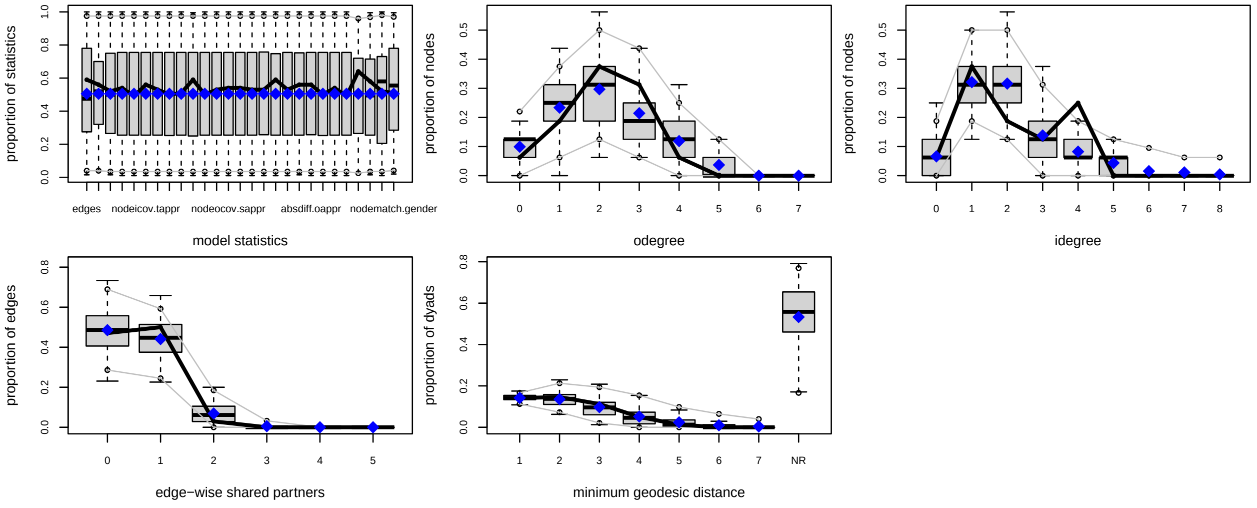

```
## [1] "Class 13"
```

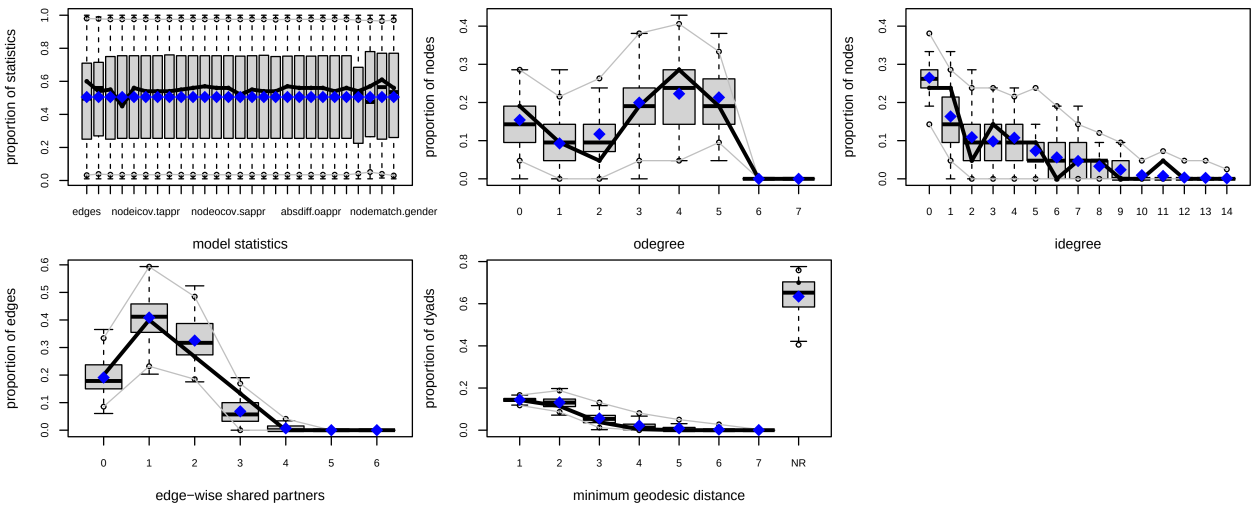


```
## [1] "Class 14"
```

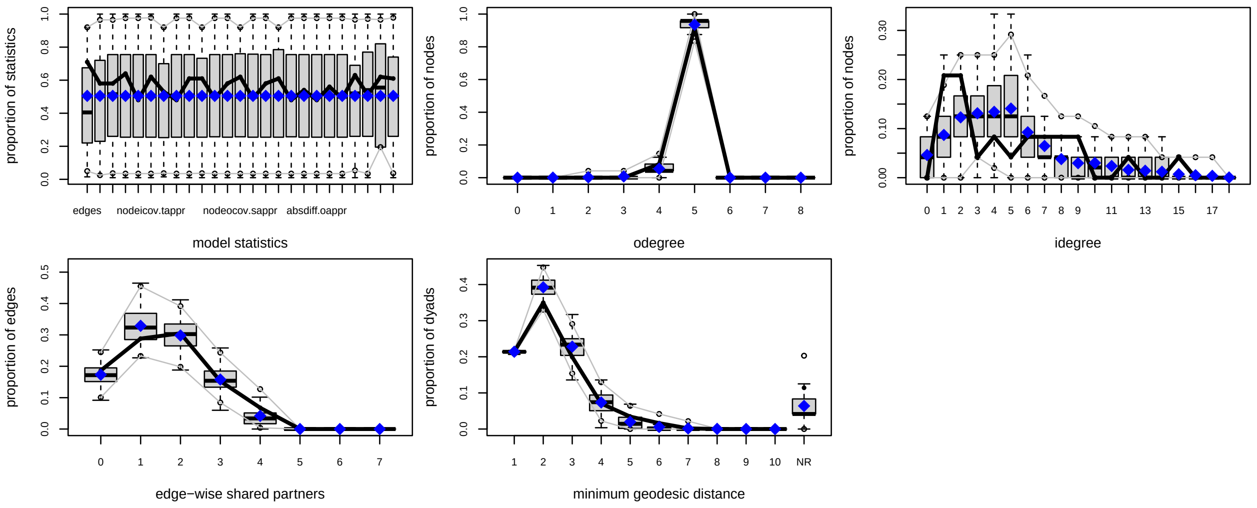


```
## [1] "Class 15"
```

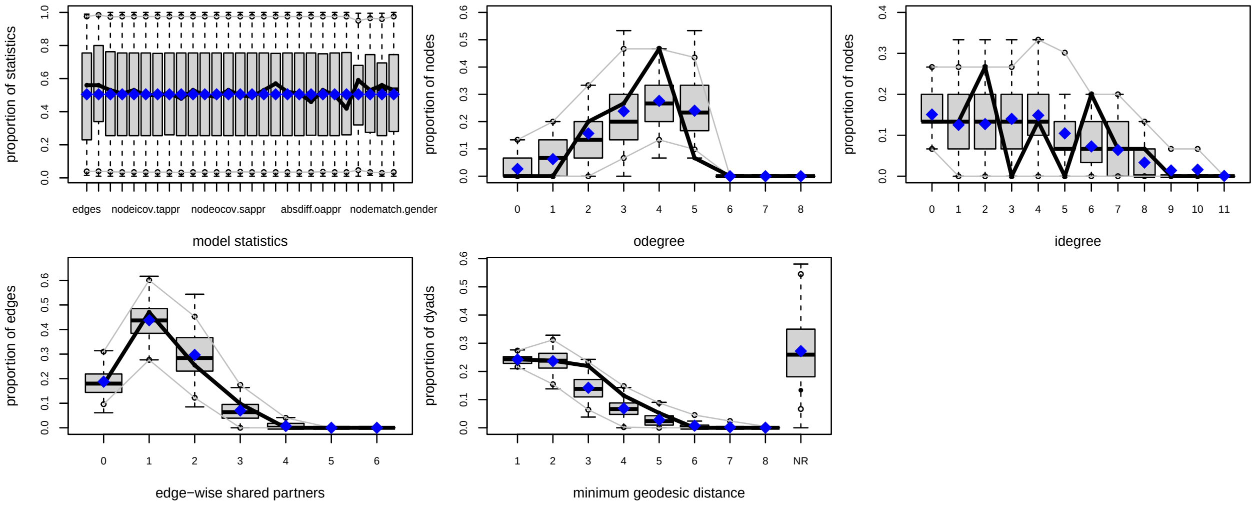

```
## [1] "Class 16"
```

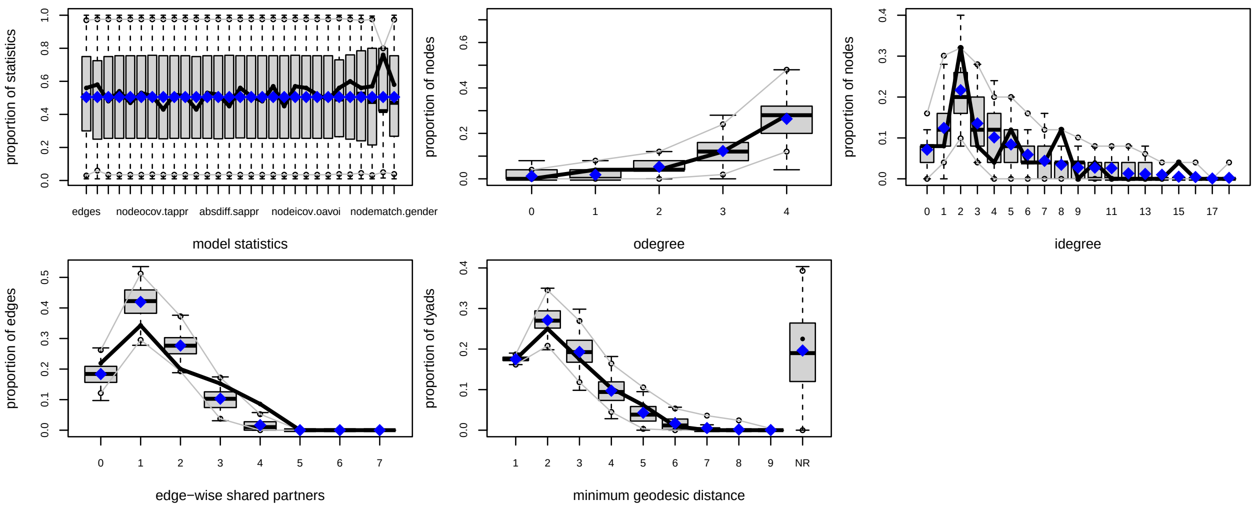
proportion of statistics
edges
nodeocov.tappr
absdiff.sappr
nodeicov.oavoi
nodematch.gender
model statistics
proportion of nodes
odegree
proportion of nodes
idegree
proportion of edges
edge−wise shared partners
proportion of dyads
minimum geodesic distance
NR

Model 5: Interaction Model

R Code

```
#### Model 5: ERGM friendship interaction model ####
ergm_sp_friendship_interaction_list <- vector(mode = "list", length=length(SP_mn_only_list))
for(i in 1:16){
  ergm_sp_friendship_interaction_list[[i]] <-
    ergm(SP_mn_only_list[[i]] ~
           edges +
           mutual +
           twopath +
           gwidegree(0.5,fixed=T) +
           gwesp(0.5,fixed=T) +
           nodeicov("oappr") +
           nodeicov("oappr") : edgecov(FR_mn_only_list[[i]]) +
           nodeocov("oappr") +
           absdiff("oappr") +
           absdiff("oappr") : edgecov(FR_mn_only_list[[i]]) +
           nodeicov("oavoi") +
           nodeicov("oavoi") : edgecov(FR_mn_only_list[[i]]) +
           nodeocov("oavoi") +
           absdiff("oavoi") +
           absdiff("oavoi") : edgecov(FR_mn_only_list[[i]]) +
           edgecov(FR_mn_only_list[[i]]) +
           nodeifactor("gender") +
           nodeofactor("gender") +
           nodematch("gender"),
         control = control.ergm(seed = 1),
         constraints = ~bd(maxout = 5)
    )
}

#### Meta-Analysis ####
ergm_sp_friendship_interaction_df <- ergm_sp_friendship_interaction_list %>%
  lapply(broom::tidy) %>%
  purrr::reduce(full_join,by="term") %>%
  .[,which(c(1,rep(c(1,1,0,0,0),times=16))==1)]

ergm_sp_friendship_interaction_list <-
  list(ergm_sp_friendship_interaction_edges =
         matrix(unlist(ergm_sp_friendship_interaction_df[1,-1]),ncol=2,byrow=T)  %>%
         as.data.frame %>%
         `colnames<-`(c("coef","se")),
       ergm_sp_friendship_interaction_mutual =
         matrix(unlist(ergm_sp_friendship_interaction_df[2,-1]),ncol=2,byrow=T) %>%
         as.data.frame %>%
         `colnames<-`(c("coef","se")),
       ergm_sp_friendship_interaction_twopath =
         matrix(unlist(ergm_sp_friendship_interaction_df[3,-1]),ncol=2,byrow=T) %>%
         as.data.frame %>%
         `colnames<-`(c("coef","se")),
       ergm_sp_friendship_interaction_gwideg =
         matrix(unlist(ergm_sp_friendship_interaction_df[4,-1]),ncol=2,byrow=T) %>%
```

```
      as.data.frame %>%
      `colnames<-`(c("coef","se")),
    ergm_sp_friendship_interaction_gwesp =
      matrix(unlist(ergm_sp_friendship_interaction_df[5,-1]),ncol=2,byrow=T) %>%
      as.data.frame %>%
      `colnames<-`(c("coef","se")),
    ergm_sp_friendship_interaction_nodeicov.oappr =
      matrix(unlist(ergm_sp_friendship_interaction_df[6,-1]),ncol=2,byrow=T) %>%
      as.data.frame %>%
      `colnames<-`(c("coef","se")),
    ergm_sp_friendship_interaction_nodeicov.oappr.FR =
      matrix(unlist(ergm_sp_friendship_interaction_df[7,-1]),ncol=2,byrow=T) %>%
      as.data.frame %>%
      `colnames<-`(c("coef","se")),
    ergm_sp_friendship_interaction_nodeocov.oappr =
      matrix(unlist(ergm_sp_friendship_interaction_df[8,-1]),ncol=2,byrow=T) %>%
      as.data.frame %>%
      `colnames<-`(c("coef","se")),
    ergm_sp_friendship_interaction_absdiff.oappr =
      matrix(unlist(ergm_sp_friendship_interaction_df[9,-1]),ncol=2,byrow=T) %>%
      as.data.frame %>%
      `colnames<-`(c("coef","se")),
    ergm_sp_friendship_interaction_absdiff.oappr.FR =
      matrix(unlist(ergm_sp_friendship_interaction_df[10,-1]),ncol=2,byrow=T) %>%
      as.data.frame %>%
      `colnames<-`(c("coef","se")),
    ergm_sp_friendship_interaction_nodeicov.oavoi =
      matrix(unlist(ergm_sp_friendship_interaction_df[11,-1]),ncol=2,byrow=T) %>%
      as.data.frame %>%
      `colnames<-`(c("coef","se")),
    ergm_sp_friendship_interaction_nodeicov.oavoi.FR =
      matrix(unlist(ergm_sp_friendship_interaction_df[12,-1]),ncol=2,byrow=T) %>%
      as.data.frame %>%
      `colnames<-`(c("coef","se")),
    ergm_sp_friendship_interaction_nodeocov.oavoi =
      matrix(unlist(ergm_sp_friendship_interaction_df[13,-1]),ncol=2,byrow=T) %>%
      as.data.frame %>%
      `colnames<-`(c("coef","se")),
    ergm_sp_friendship_interaction_absdiff.oavoi =
      matrix(unlist(ergm_sp_friendship_interaction_df[14,-1]),ncol=2,byrow=T) %>%
      as.data.frame %>%
      `colnames<-`(c("coef","se")),
    ergm_sp_friendship_interaction_absdiff.oavoi.FR =
      matrix(unlist(ergm_sp_friendship_interaction_df[15,-1]),ncol=2,byrow=T) %>%
      as.data.frame %>%
      `colnames<-`(c("coef","se")),
    ergm_sp_friendship_interaction_edgecov.FR =
      matrix(unlist(ergm_sp_friendship_interaction_df[16,-1]),ncol=2,byrow=T) %>%
      as.data.frame %>%
      `colnames<-`(c("coef","se")),
    ergm_sp_friendship_interaction_nodeifactor.gender.2 =
      matrix(unlist(ergm_sp_friendship_interaction_df[17,-1]),ncol=2,byrow=T) %>%
      as.data.frame %>%
```

```r
          `colnames<-`(c("coef","se")),
        ergm_sp_friendship_interaction_nodeofactor.gender.2 =
          matrix(unlist(ergm_sp_friendship_interaction_df[18,-1]),ncol=2,byrow=T) %>%
          as.data.frame %>%
          `colnames<-`(c("coef","se")),
        ergm_sp_friendship_interaction_nodematch.gender =
          matrix(unlist(ergm_sp_friendship_interaction_df[19,-1]),ncol=2,byrow=T) %>%
          as.data.frame %>%
          `colnames<-`(c("coef","se")),
        ergm_sp_friendship_interaction_nodeifactor.gender.3 =
          matrix(unlist(ergm_sp_friendship_interaction_df[20,-1]),ncol=2,byrow=T) %>%
          as.data.frame %>%
          `colnames<-`(c("coef","se")),
        ergm_sp_friendship_interaction_nodeofactor.gender.3 =
          matrix(unlist(ergm_sp_friendship_interaction_df[21,-1]),ncol=2,byrow=T) %>%
          as.data.frame %>%
          `colnames<-`(c("coef","se")))



par_sp_friendship_interaction <-
  c("edges","mutual","twopath","gwid","gwesp",
    "nodeicov.oappr","nodeicov.oappr:FR","nodeocov.oappr","absdiff.oappr","absdiff.oappr:FR",
    "nodeicov.oavoi","nodeicov.oavoi:FR","nodeocov.oavoi","absdiff.oavoi","absdiff.oavoi:FR",
    "edgecov.FR","nodeifactor.gender.2","nodeofactor.gender.2",
    "nodematch.gender","nodeifactor.gender.3","nodeofactor.gender.3")
sp_friendship_interaction_ma_list <-
  vector(mode = "list", length=length(par_sp_friendship_interaction)) %>%
  `names<-`(par_sp_friendship_interaction)
for(i in 1:length(par_sp_friendship_interaction)){
  sp_friendship_interaction_ma_list[[i]] <-
    rma(yi=coef,sei=se,data=ergm_sp_friendship_interaction_list[[i]],
        control = list(stepadj=0.5,maxiter=1000))
}

# summary table
lapply(sp_friendship_interaction_ma_list,broom::tidy) %>%
  purrr::reduce(rbind) %>%
  cbind(par_sp_friendship_interaction,.) %>%
  mutate(estimate = round(estimate,4),
         std.error = round(std.error,4),
         statistic = round(statistic,4),
         p.value = round(p.value,4)) %>%
  select(c(1,4,5,7))
```

Goodness of Fit Plots

```
## [1] "Class 1"
```

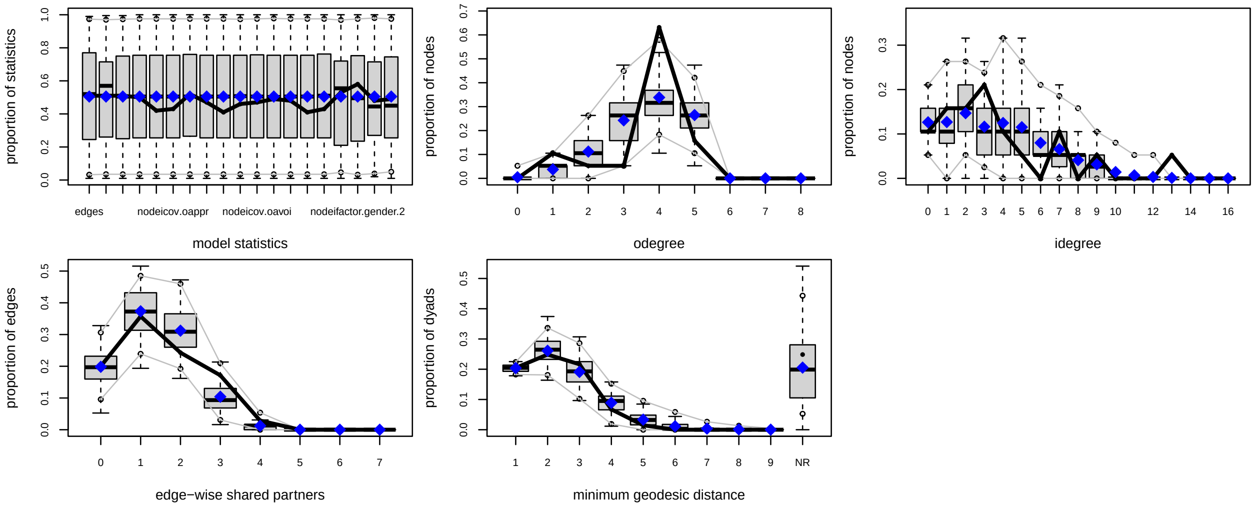


```
## [1] "Class 2"
```

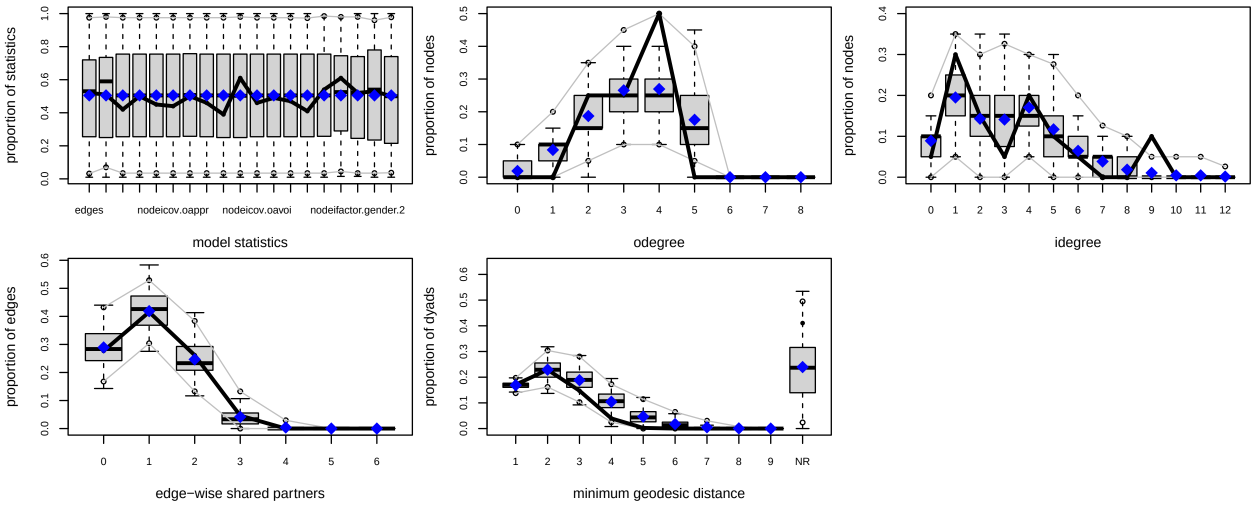


```
## [1] "Class 3"
```

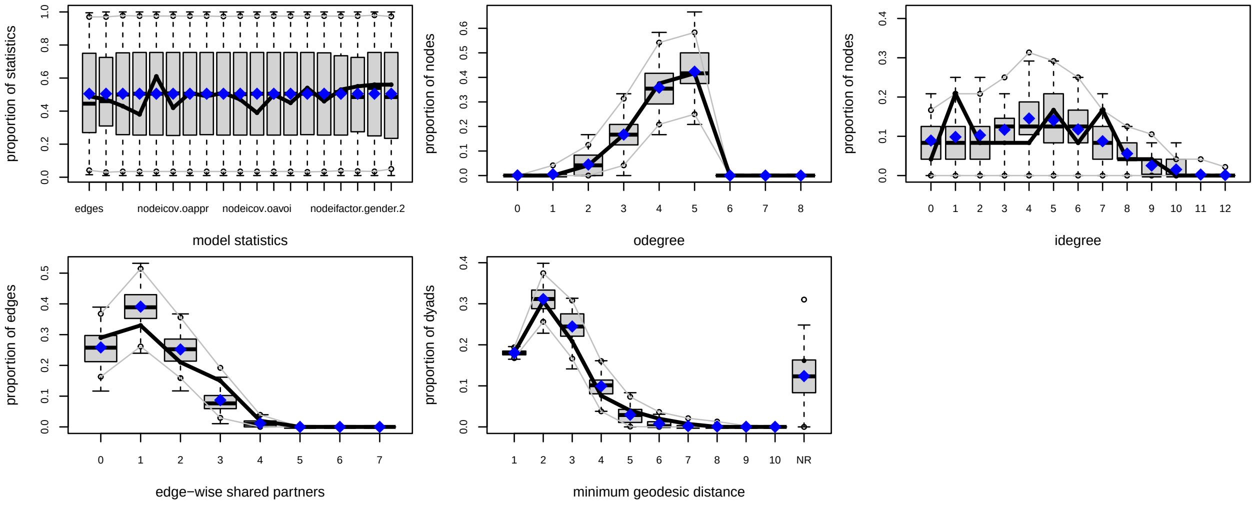

```
## [1] "Class 4"
```

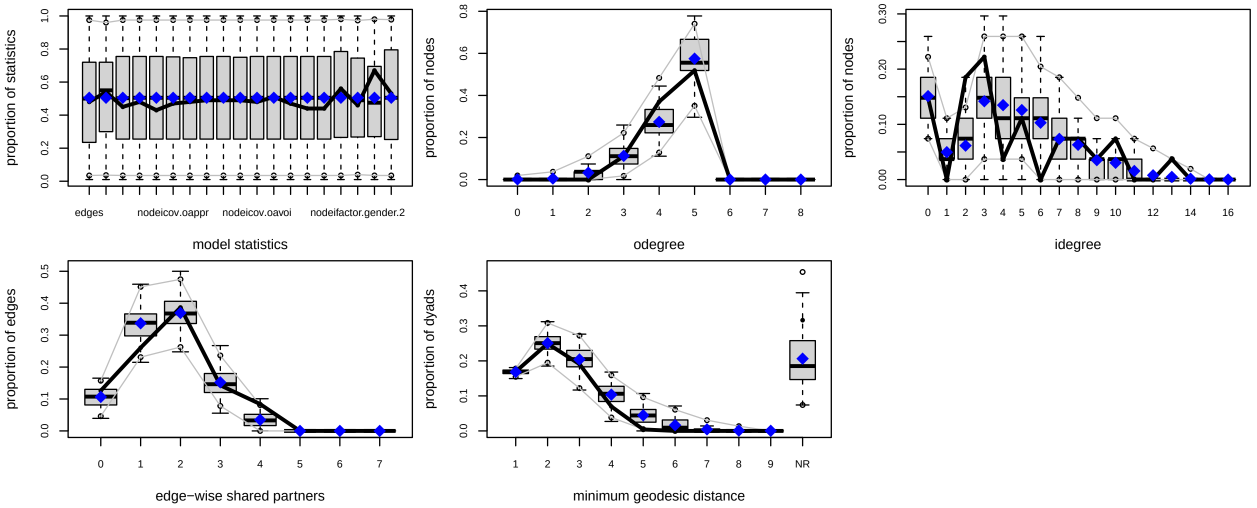


```
## [1] "Class 5"
```

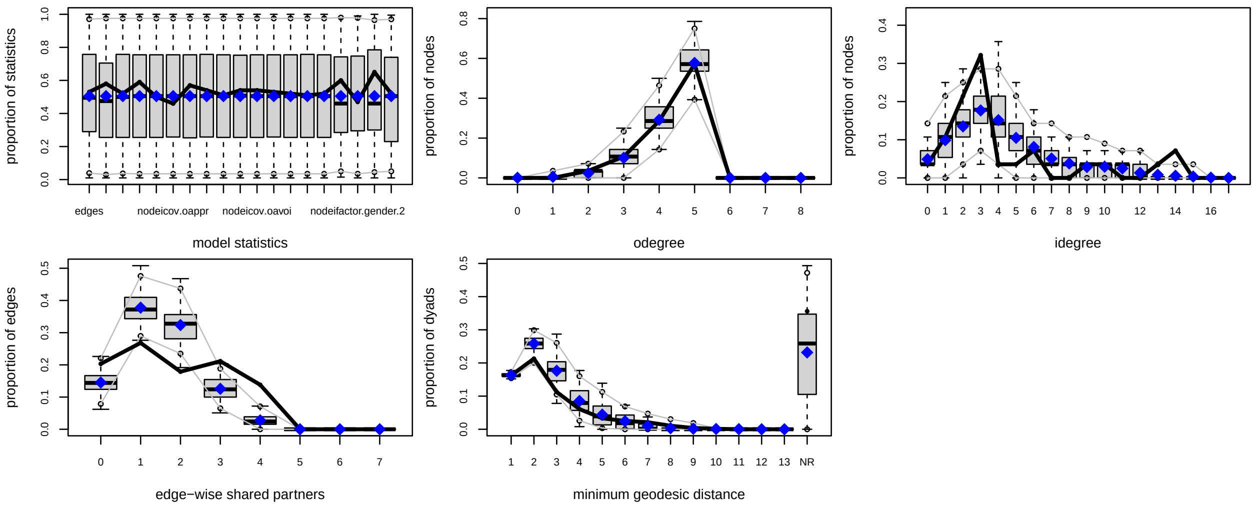


```
## [1] "Class 6"
```

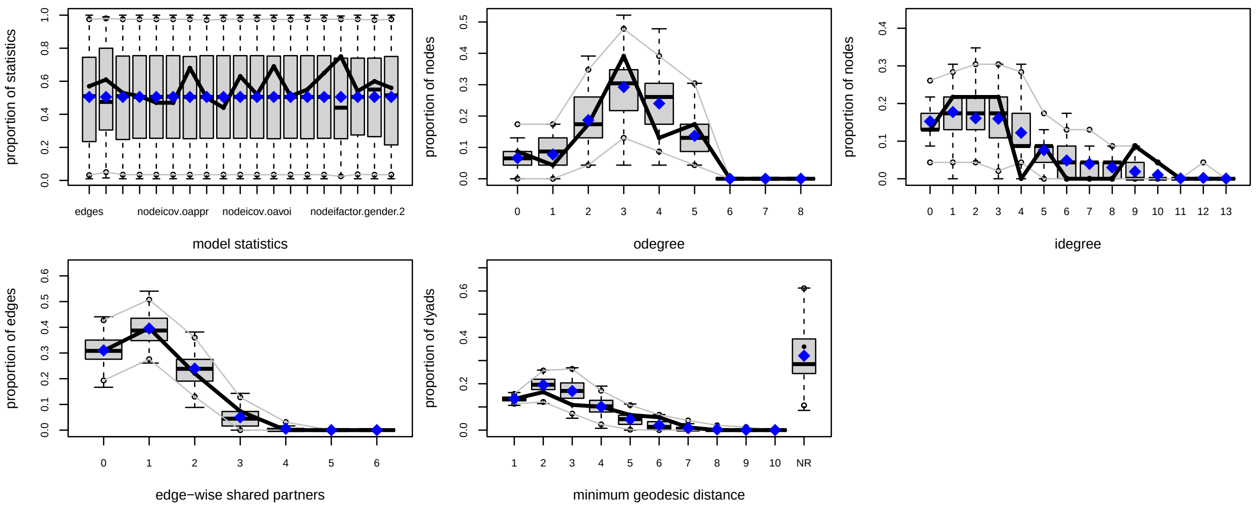

[1] "Class 7"

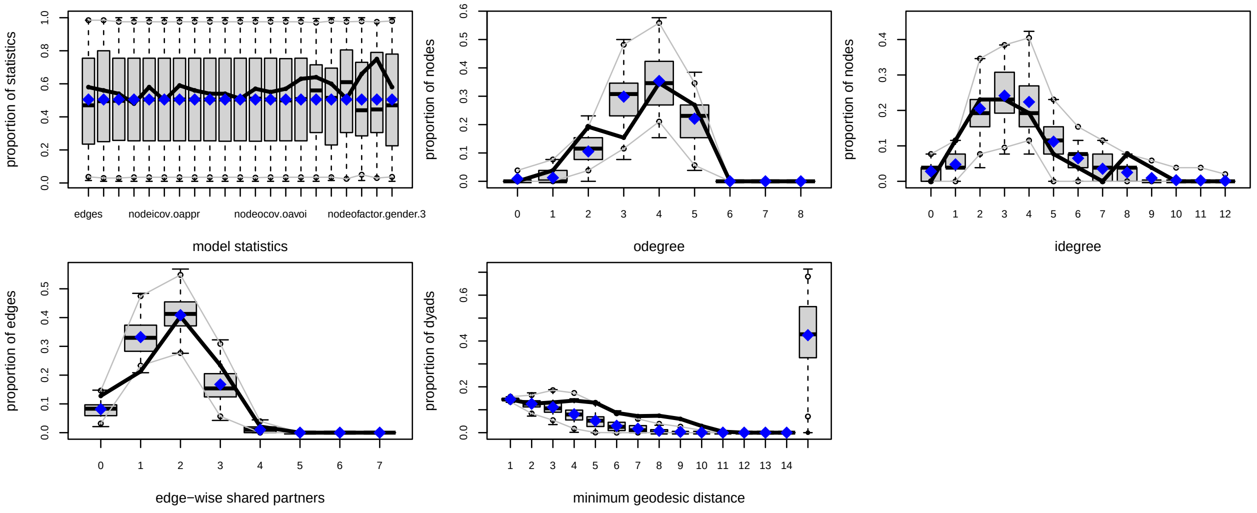

[1] "Class 8"

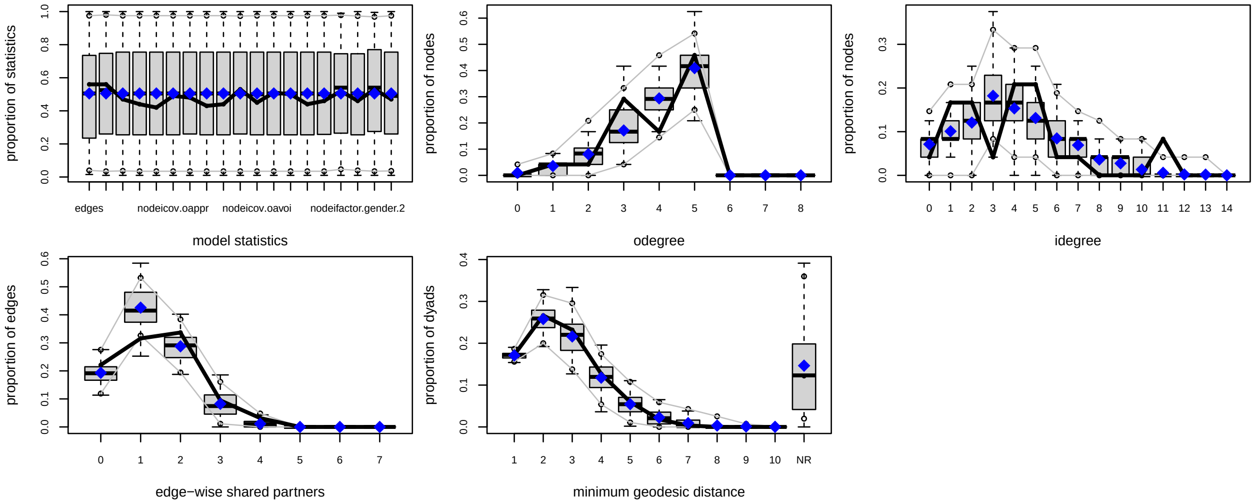

[1] "Class 9"

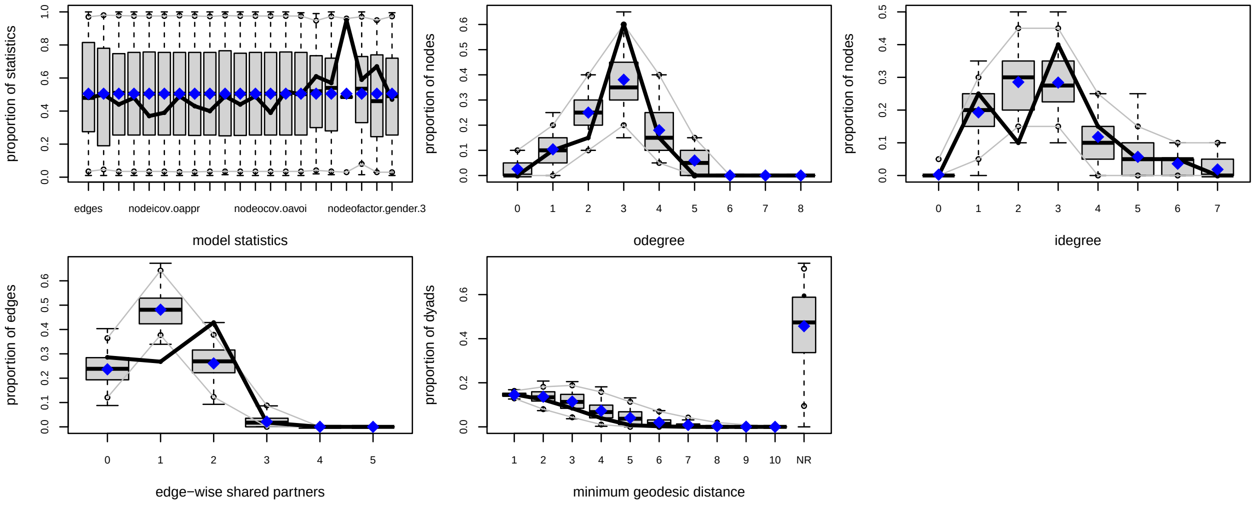

[1] "Class 10"

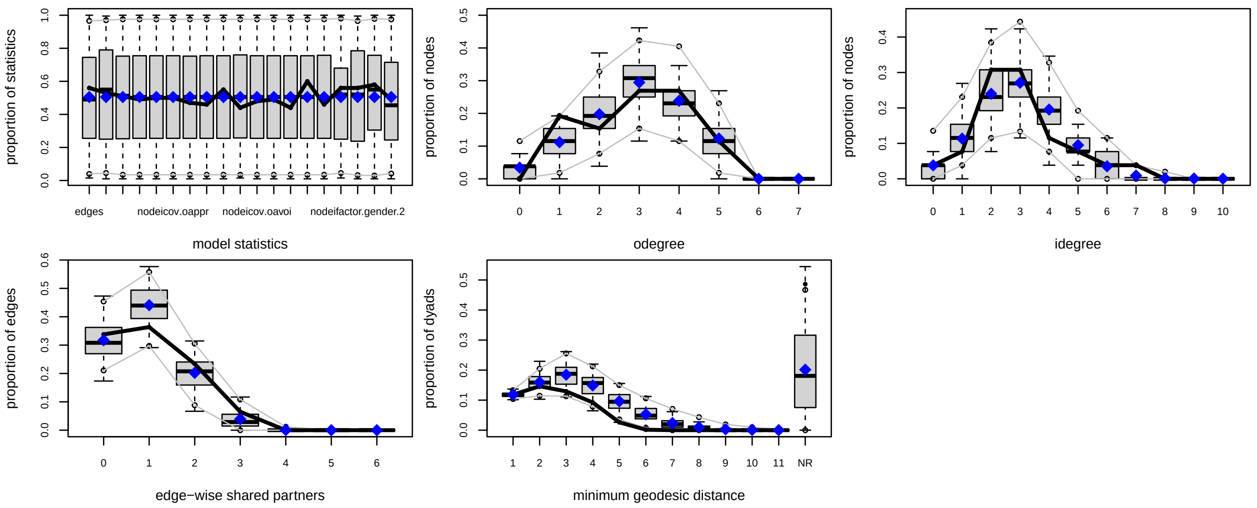

[1] "Class 11"

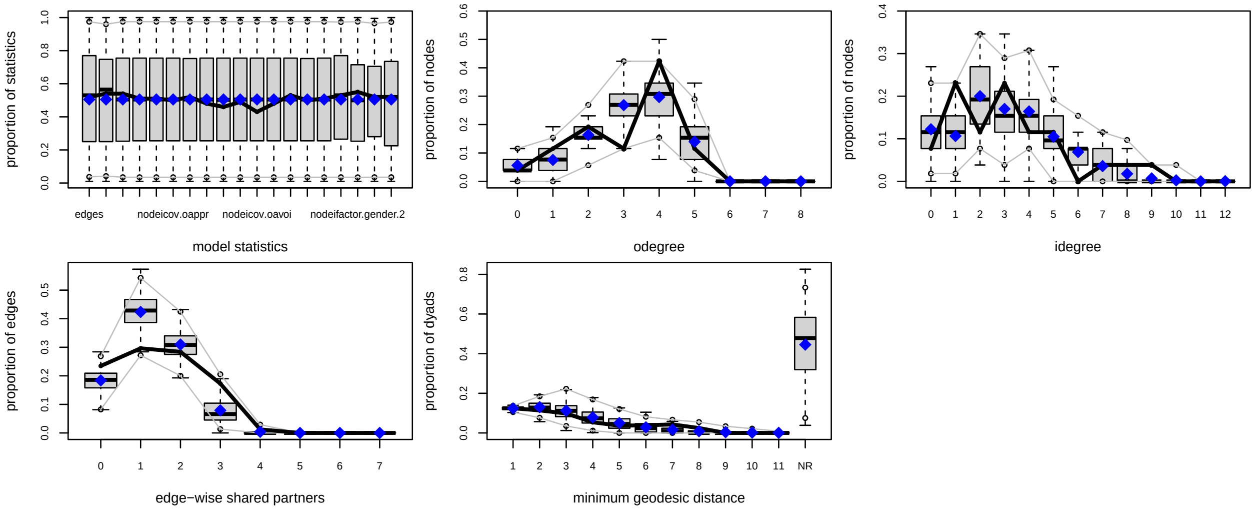

[1] "Class 12"

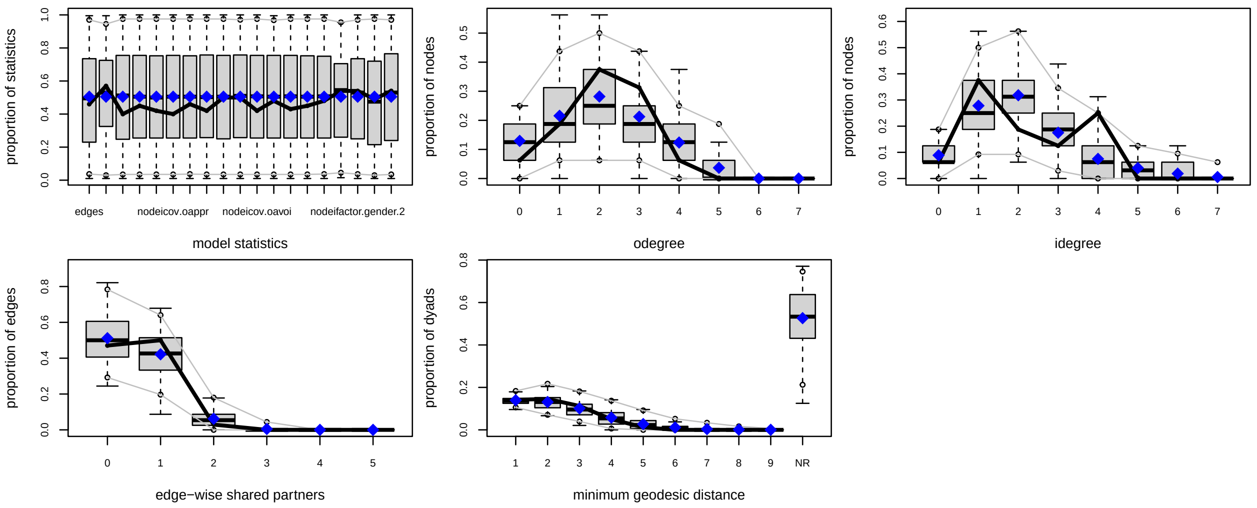

```
## [1] "Class 13"
```

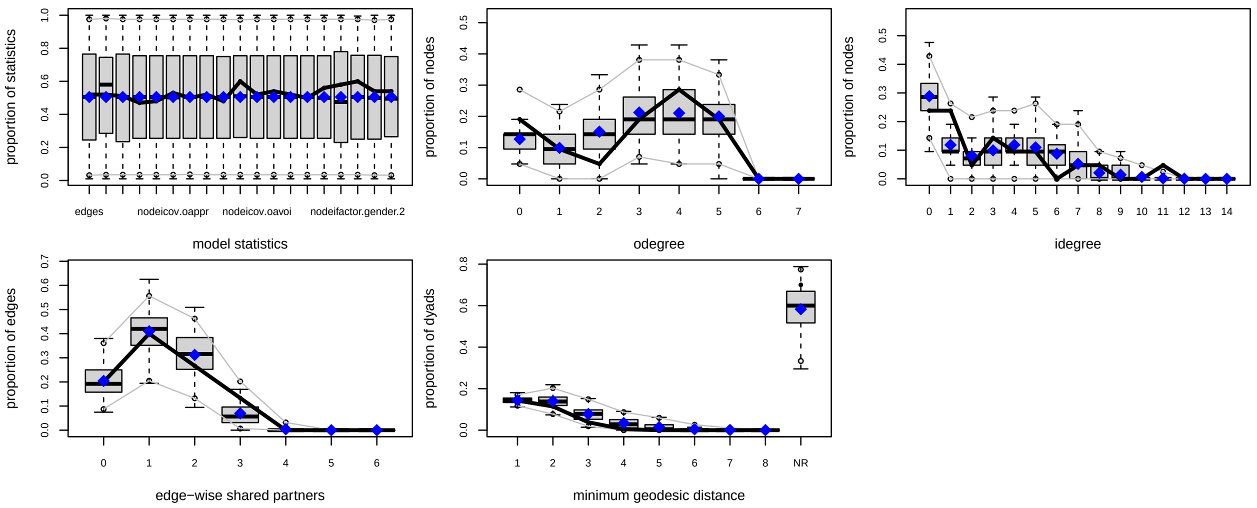


```
## [1] "Class 14"
```

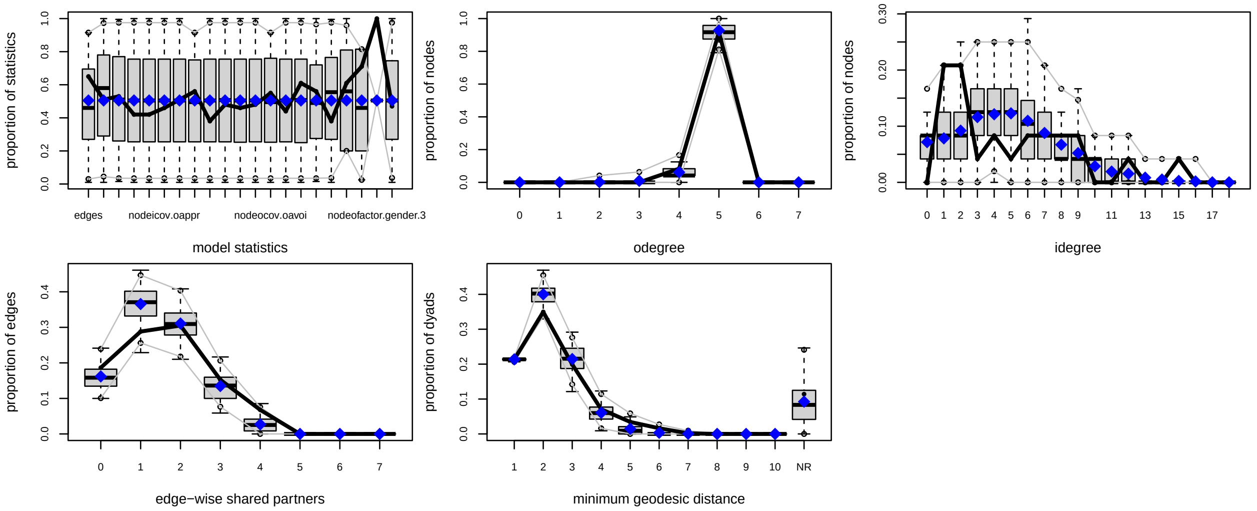


```
## [1] "Class 15"
```

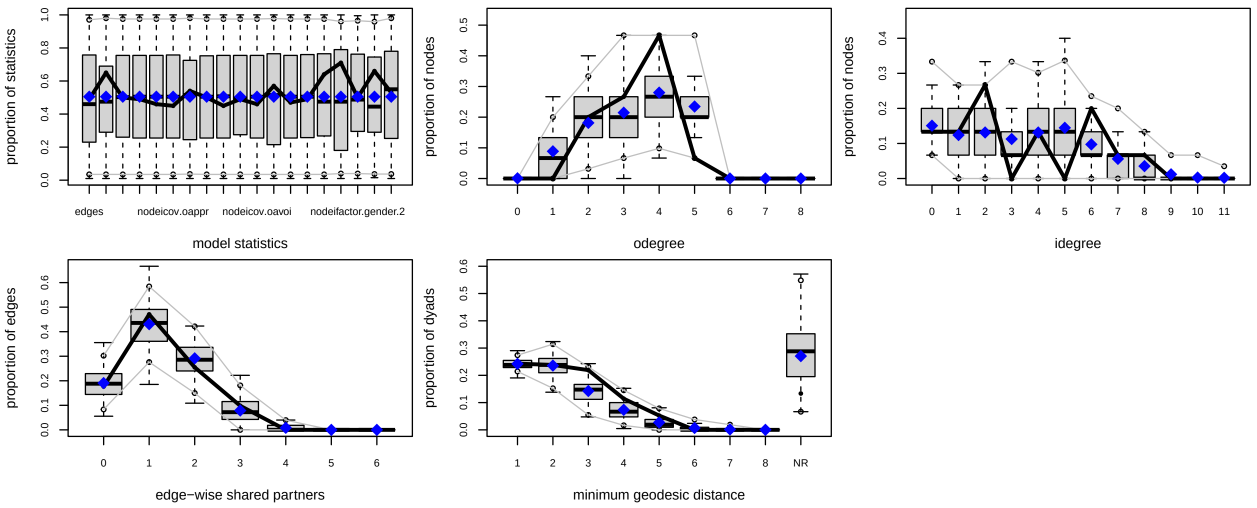

```
## [1] "Class 16"
```

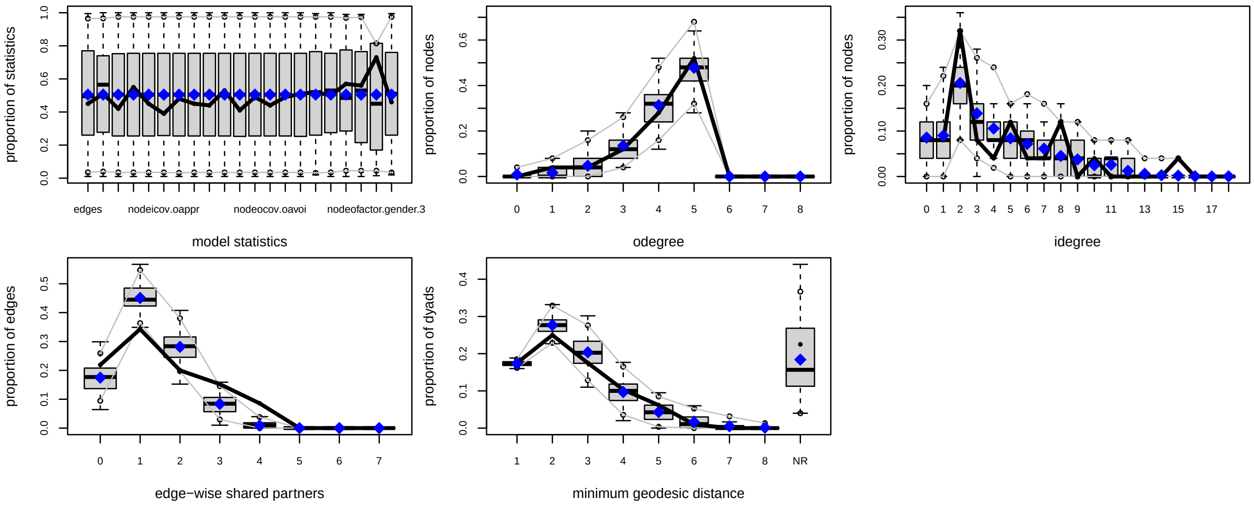
proportion of statistics
edges
nodeicov.oappr
nodeocov.oavoi
nodeofactor.gender.3
model statistics
proportion of nodes
odegree
proportion of nodes
idegree
proportion of edges
edge−wise shared partners
proportion of dyads
NR
minimum geodesic distance

Appendix 5: Interpretation of Network Endogenous Effects

We found negative arc effects, suggesting that sports game ties occur relatively rarely. Furthermore, our analysis revealed a positive effect for reciprocity. The two-paths showed a negative effect, while triadic closure emerges as a positive effect, suggesting that students in sports teams in our sample tend to triadic relations. The popularity effect, that describes the general tendency for centralization when controlling for all other predictors shows mixed results. In models 1 and 2, there is a positive and significant popularity effect, indicating that students with high and low indegree receive similar amounts of nominations (Levy, 2016). In model 3, the effect is negative, showing that there are some students who receive a lot of nominations compared to others. This may be linked to the omission of goal orientation, as without the inclusion of goal orientation in the models, there are unaccounted differences in indegree between students. In model 4, when including goal orientation, gender and friendship, the effect is non-significant. This indicates that the differences in indegree between students can be fully explained by the predictor variables and there is no additional general preference to nominate students because of popularity. In total, these findings align with common dynamics of network-self-organizations seen in school-based peer group formation (Lusher & Robins, 2013a, 2013c).

References

Block, P. (2015). Reciprocity, transitivity, and the mysterious three-cycle. *Social Networks, 40*, 163-173. https://doi.org/10.1016/j.socnet.2014.10.005

Levy, M. A. (2016). gwdegree: Improving interpretation of geometrically-weighted degree estimates in exponential random graph models. *The Journal of Open Source Software, 1*(3), 36. https://doi.org/10.21105/joss.00036

Lusher, D., & Robins, G. (2013a). Example exponential random graph model analysis. In D. Lusher, J. Koskinen, & G. Robins (Eds.), *Exponential Random Graph Models for Social Networks: Theory, Methods, and Applications* (pp. 37-46). Cambridge University Press. https://doi.org/10.1017/CBO9780511894701.006

Lusher, D., & Robins, G. (2013b). Personal Attitudes, Perceived Attitudes, and Social Structures: A Social Selection Model. In D. Lusher, J. Koskinen, & G. Robins (Eds.), *Exponential Random Graph Models for Social Networks: Theory, Methods, and Applications* (pp. 189–201). Cambridge University Press. https://doi.org/10.1017/CBO9780511894701.017

Robins, G., Pattison, P., & Wang, P. (2009). Closure, connectivity and degree distributions: Exponential random graph (p) models for directed social networks. *Social Networks, 31*(2), 105-117. https://doi.org/10.1016/j.socnet.2008.10.006

Stadtfeld, C., & Amati, V. (2021). Network mechanism and network models. In G. Manzo (Eds.), *Research handbook on analytical sociology* (pp. 432-452). Edward Elgar Publishing https://doi.org/10.4337/9781789906851.00032
